# Synthesis, Characterization and Biological Evaluation of New 3,5-Disubstituted-Pyrazoline Derivatives as Potential Anti-*Mycobacterium tuberculosis* H37Ra Compounds

**DOI:** 10.3390/molecules26072081

**Published:** 2021-04-05

**Authors:** Kok Tong Wong, Hasnah Osman, Thaigarajan Parumasivam, Unang Supratman, Mohammad Tasyriq Che Omar, Mohamad Nurul Azmi

**Affiliations:** 1 School of Chemical Sciences, Universiti Sains Malaysia, Minden, Penang 11800, Malaysia; koktong_850820@hotmail.com (K.T.W.); ohasnah@usm.my (H.O.); 2 School of Pharmaceutical Sciences, Universiti Sains Malaysia, Minden, Penang 11800, Malaysia; thaigarp@usm.my; 3 Department of Chemistry, Faculty of Mathematics and Natural Sciences, Universitas Padjadjaran, Jatinangor 45363, Indonesia; unang.supratman@unpad.ac.id; 4 Biological Section, School of Distance Education, Universiti Sains Malaysia, Penang 11800, Malaysia; mtasyriq@usm.my

**Keywords:** pyrazolines, *Mycobacterium tuberculosis*, antitubercular agents, molecular docking, alpha-sterol demethylase

## Abstract

A total of fourteen pyrazoline derivatives were synthesized through cyclo-condensation reactions by chalcone derivatives with different types of semicarbazide. These compounds were characterized by IR, 1D-NMR (^1^H, ^13^C and Distortionless Enhancement by Polarization Transfer - DEPT-135) and 2D-NMR (COSY, HSQC and HMBC) as well as mass spectroscopy analysis (HRMS). The synthesized compounds were tested for their antituberculosis activity against *Mycobacterium tuberculosis* H37Ra in vitro. Based on this activity, compound **4a** showed the most potent inhibitory activity, with a minimum inhibitory concentration (MIC) value of 17 μM. In addition, six other synthesized compounds, **5a** and **5c–5g**, exhibited moderate activity, with MIC ranges between 60 μM to 140 μM. Compound **4a** showed good bactericidal activity with a minimum bactericidal concentration (MBC) value of 34 μM against *Mycobacterium tuberculosis* H37Ra. Molecular docking studies for compound **4a** on alpha-sterol demethylase was done to understand and explore ligand–receptor interactions, and to hypothesize potential refinements for the compound.

## 1. Introduction

**Figure 1:**
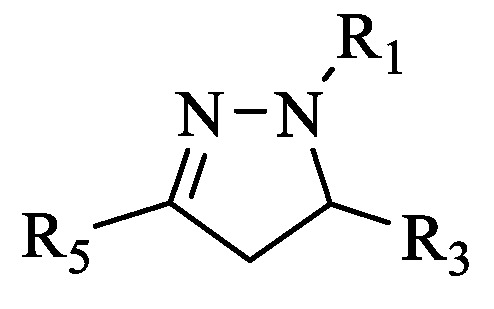
The general structure of the pyrazoline at position 1-, 3- and 5-.

Pyrazoline is a five-membered heterocyclic ring that has an endocyclic double bond with two adjacent nitrogen atoms at position 1- and 2-. The first pyrazoline (4,5-dihydro-*1H*-pyrazole) was synthesized by Knorr in 1883 [1]. Pyrazolines are noted for the stability of their ring system and the reactivity of several sites that permit a series of substitution reactions to take place. Structural modifications of pyrazolines can be achieved by decorating the stable fragments with different functional groups and aromatic scaffolds to create benzylhydrazine moieties for the development of new potent compounds possessing biological activities. Previous studies have reported considerable biological activities when substitution occurs at position 1-, 3- and 5- of the pyrazoline (Figure 1), such as anticonvulsant, antimalarial [2], anti-cardiovascular [3], anticancer [4], anti-amoebic [5], antimicrobial [6,7] and anti-tumor activities [8]. Meanwhile, carbothioamide derivatives were found to have significant pharmacological activities, such as monoamine oxidase (MAO) inhibitors [9,10], antituberculosis [11,12,13] and anticonvulsant activities [14,15,16].

Tuberculosis (TB) is an infectious disease caused by *Mycobacterium tuberculosis* (MTB) that affects the lungs [17]. According to the World Health Organization (WHO) 2019 Global Tuberculosis Report, about 1.5 million people from a total of 10 million people with TB died in 2018 [18]. In addition, WHO has declared that the South-East Asian region is the most affected with TB cases (44%), followed by Africa (24%), Western Pacific (18%), Eastern Mediterranean (8%), Americas (3%) and Europe (3%). In Malaysia, the Health Ministry (2019) has reported about 1500 to 2000 deaths from TB per year, with an average of six deaths a day in 2018 [19]. The increase in cases is mainly due to delays in seeking medical treatment and low TB awareness. In addition, the current lengthy treatment duration impairs patient adherence to the TB medications, which in turn fuels the spread of highly concerning multi-drug and extensively-drug resistant *M. tuberculosis*. Hence, this underscores the importance of developing a new effective anti-TB regimen.

In continuation of our efforts to develop antitubercular agents, here we report the synthesis of new pyrazoline derivatives, along with their potential anti-TB activity against *M. tuberculosis* H37Ra. The attenuated *Mycobacterium tuberculosis* H37Ra strain was used due to biosafety constraints with using virulent *M. tuberculosis* H37Rv and clinical strains. However, Heinrichs et al. showed that the H37Ra strain is able to equally predict susceptibility to antibiotics as the H37Rv strain and clinical isolates [20]. This justifies the selection of avirulent *M. tuberculosis* for this study. Molecular docking at the active site of cytochrome P450 14 alpha-sterol demethylase (CYP51) was performed to rationalize the inhibitory property of potent compounds within the active pocket.

## 2. Results and Discussion

### 2.1. Chemistry

**Scheme 1:**
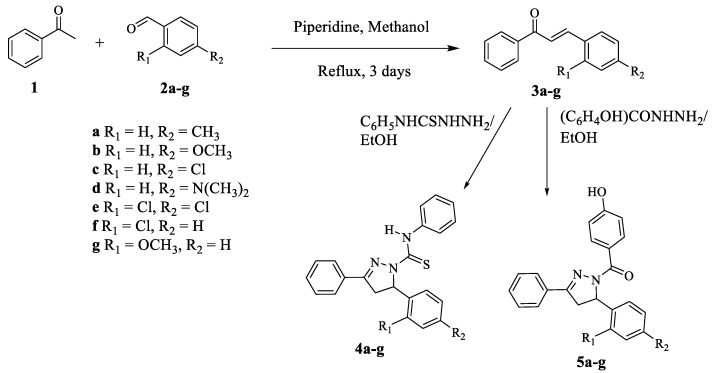
Synthesis of chalcone and pyrazoline derivatives.

The synthesis of the first series of 3,5-disubstituted-4,5-dihydro-*N*-phenyl-1*H*-pyrazole-1-carbothioamide (**4a**–**g**) was synthesized by the cyclo-condensation of chalcone derivatives (**3a**–**g**) with 4-phenyl-3-thiosemicarbazide in the presence of sodium hydroxide as a base (Scheme 1). 

Compound **4a** is the representative compound from the first series. This new compound was synthesized by the cyclo-condensation of 3-(4-methylphenyl)-1-phenyl-2-propen-1-one (**3a**) with 4-phenyl-3-thiosemicarbazide in the presence of sodium hydroxide as a base. Compound **4a** formed as a cream-colored precipitate with 26% yield and a melting point of 133–136 °C. Compound **4a** was characterized by various spectroscopic techniques including IR, 1D-NMR, 2D-NMR and HRMS. The important absorption bands in the IR spectrum of compound **4a** were observed at 3291 cm^−1^ (N–H stretching), 1594 cm^−1^ (C=N stretching), 1520 cm^−1^ and 1449 cm^−1^ (C=C stretching of the aromatic), and 1399 cm^−1^ (C=S stretching). The absence of C=O and C=C bands, as well as the appearance of new C=N and C=S bands in the IR spectra of compound **4a** suggests the complete formation of compound **4a** via the cyclo-condensation reaction between chalcone **3a** and 4-phenyl-3-thiosemicarbazide (Scheme 1).

**Figure 2:**
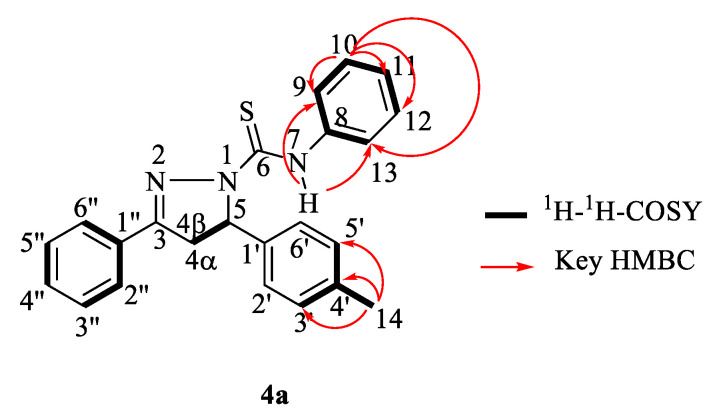
Key COSY/HMBC correlations of compound **4a**.

The ^1^H-NMR spectrum of compound **4a** showed that two germinal protons, H-4α and H-4β, of the methylene group resonated as a doublet of doublet at δ_H_ 3.26 and δ_H_ 4.05, respectively. The appearance of these two signals could be attributed to the non-equivalent nature of the two germinal protons with a *J* coupling constant of *J_4α4β_* = 18.0 Hz, *J_4α5_* = 3.5 Hz and *J_4β5_* = 11.5 Hz. Meanwhile, the vicinal proton (H-5) also appeared as a doublet of doublet at a slightly downfield region, δ_H_ 6.14, due to vicinal coupling with the two neighboring germinal protons of the methylene group at position 4- of the pyrazoline ring. A singlet at δ_H_ 9.93 in the downfield region indicated the presence of a thioamide proton. In compound **4a**, some aromatic protons such as H-3″, H-4″ and H-5″ were observed at the same chemical shift, δ_H_ 7.49, due to the overlapping of peaks in the similar environment. The ^1^H-NMR spectrum also showed three signals for ring C. A triplet at δ_H_ 7.33 (2H, *J* = 7.5 Hz) was attributed to H-10 and H-12. In addition, two multiplets at δ_H_ 7.72 (2H) and δ_H_ 7.16 were assigned to H-9 with H-13 and H-11. The ^13^C-NMR spectrum of carbothioamide compound **4a** showed seventeen carbon signals. One carbon signal each at δ_C_ 42.3 and δ_C_ 62.3 suggested the presence of pyrazoline ring carbons, which were assigned to 4-CH_2_ and 5-CH, respectively. Also, a signal at δ_C_ 155.3 was attributed to 3-C=N in the pyrazoline ring, which is a carbon attached to an electronegative nitrogen by a double bond and also to a benzene ring. Besides, a signal at δ_C_ 174.2 was attributed to 6-C=S. The 2D-NMR correlation of ^1^H-^1^H COSY and ^1^H-^13^C HMBC spectra were used to assign the aromatic signals, especially in the case of the pyrazoline ring. The analysis of ^1^H-^1^H COSY showed the correlation between of 14-CH_3_-H-4′-H-3′ and H-5′, 4α-CH with 4β-CH and 5-CH, H-8 with H-9-H-10-H-11-H-12 and H-2″ with H-1″-H-6″-H-3″, H-4″ with H-5″ (Figure 2). The ^1^H-^13^C HMBC spectrum of **4a** shows the cross-correlations of 7-NH with carbons C-9 and C-13, and H-10 with C-9, C-13, C-11, C-12 and C-8 (Figure 2), and the cross-correlation of 14-CH_3_ with C-3′, C-5′ and C-4′. The HRMS spectrum of **4a** revealed the molecular ion peak [M + Na]^+^ at m/z 394.1156, which is consistent with the molecular formula C_23_H_21_N_3_S (calcd. 394.1354). According to the above spectra data analysis, compound **4a** was identified as a new 2-pyrazoline compound and named 5-(4-methylphenyl)-*N*,3-diphenyl-4,5-dihydro-1*H*-pyrazole-1-carbothioamide.

The second series of new (3,5-disubstituted- 4,5-dihydro-1*H*-pyrazol-yl) (4-hydroxyphenyl)methanone (**5a–g**) was synthesized by the cyclo-condensation of chalcone derivatives (**3a–g**) with 4-hydroxybenzhydrazide in the presence of sodium hydroxide as a base. 

Compound **5e** was taken as the representative compound from the fourth series, which formed a pale-yellow powder with 17% yield and a melting point of 237–241 °C. Compound **5e** was characterized by multiple spectroscopic techniques including IR, 1D-NMR, 2D-NMR, and HRMS. The important absorption bands in the IR spectrum of compound **5e** were observed at 3137 cm^−1^ (O–H stretching), 3066 cm^−1^ (C_sp_^2^–H), 2926 cm^−1^ (C_sp_^3^–H), 1737 cm^−1^ (C=O stretching), 1618 cm^−1^ (C=N stretching), 1573 cm^−1^ and 1427 cm^−1^ (C=C stretching of the aromatic). Moreover, the hydroxy group of phenol in the *para* position is a good activating group [21]. The absence of C=C bands, as well as the appearance of new C=N and C–O bands in the IR spectrum of compound **5e** suggests the complete formation of the compound **5e** via a cyclo-condensation reaction between chalcone **3e** and 4-hydroxybenzhydrazide (Scheme 1).

**Figure 3:**
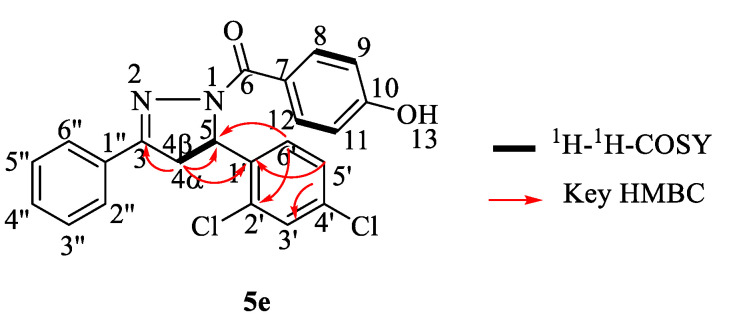
Key COSY/HMBC correlations of compound **5e**.

The ^1^H-NMR spectrum of compound **5e** revealed the presence of one, mostly downfield, sharp singlet at δ_H_ 10.11 for the hydroxy proton (13-OH). One doublet of doublet at δ_H_ 7.40 (*J* = 2.5 and 8.5 Hz) was assigned to H-5′ due to its *meta* coupling with H-3′ and *ortho* coupling with H-6′ in ring B. A doublet at δ_H_ 7.22 (*J* = 8.0 Hz) was attributed to H-6′ due to its *ortho* coupling to H-5′. In addition, a doublet at δ_H_ 7.69 was attributed to H-3 due to its *meta* coupling to H-5. On the other hand, a doublet at δ_H_ 7.91 (*J* = 8.0 Hz) integrating to two protons was assigned to H-9 and H-11, which occurred at the same position and can be considered chemically equivalent due to symmetry in the structure. The ^13^C-NMR spectrum of compound **5e** showed that signals corresponded to all twenty-two carbons in the compound. Signals at δ_C_ 58.8, 40.1 and 155.3 were assigned to 5-CH, 4-CH_2_ and 3-C=N of the pyrazoline ring, respectively, and the presence of the pyrazoline ring was further confirmed by 2D-NMR spectroscopy. The ^1^H-^1^H COSY spectrum of compound **5e** showed a very clear correlation between 5-CH (δ_C_ 5.95), 4α-CH (δ_C_ 3.13) and 4β -CH (δ_C_ 3.96). A strong correlation between H-8/H-12 (δ_C_ 114.9) and H-9/H-11 (δ_C_ 132.6) was also observed (Figure 3). The ^1^H-^13^C HMBC spectrum of **5e** shows that 4α-CH (δ_C_ 3.13) and 4β-CH (δ_C_ 3.96) were correlated with 5-CH (δ_C_ 58.8), C-1′ (δ_C_ 138.9) and 6-C=O (δ_C_ 165.2), thus confirming the formation of a pyrazoline ring (Figure 3). The cross-correlation of H-5′ with C-3′ and C-1′ was found, while H-6′ was found to correlate with 5-CH and C-2′. The HRMS of compound **5e** showed a molecular ion peak [M + H]^+^ at *m/z* 411.0694 (calcd. 411.0667) which corresponded to the molecular formula C_22_H_17_Cl_2_N_2_O_2_. In conclusion, based on the spectral data, it is proven that the compound **5e**, *N*-(4-hydroxyphenyl)(5-(2,4-dichlorolphenyl)-3-phenyl-4,5-dihydro-1*H*-pyrazol-1-yl)methanone, was successfully synthesized.

### 2.2. Anti-Tuberculosis Activity

Two series of fourteen synthesized pyrazoline derivatives (**4a**–**g** and **5a**–**g**) were screened for antituberculosis activity against *M. tuberculosis* H37Ra using the Tetrazolium bromide microplate assay (TEMA) method. Isoniazid was used as a positive control. Table 1 depicts the results of the antituberculosis activity of these compounds based on their minimum inhibitory concentration (MIC) and minimum bactericidal concentration (MBC) values.

Out of fourteen synthesized compounds, ten compounds exhibited promising activity against *M. tuberculosis,* with MIC values in the range of 17 μM to 511 μM. Compound **4a** was the most active compound, with the lowest MIC value of 17 μM (~6.25 μg/mL). In the first series **4a**–**g**, compounds **4a**, **4c**, **4e** and **4f** showed MICs of 17 μM (~6.25 μg/mL), 511 μM (~200 μg/mL), 471 μM (~200 μg/mL) and 511 μM (~200 μg/mL), respectively. Compound **4a** with the *para*-methyl substitute showed the most activity compared to other compounds in the same series. In the second series, compounds **5a**, **5c**, **5d**, **5e**, **5f** and **5g** exhibited moderate activity, with MICs of 70 μM (~25 μg/mL), 66 μM (~25 μg/mL), 65 μM (~25 μg/mL), 61 μM (~25 μg/mL), 66 μM (~25 μg/mL), and 134 μM (~50 μg/mL), respectively. Meanwhile, compound **5b** with the *para*-methoxy group showed no inhibition against *M. tuberculosis*.

These ten compounds (**4a**, **4c**, **4e**, **4f**, **5a**, and **5c–g**) were further evaluated for their bactericidal activity against *M. tuberculosis* H37Ra. Only seven compounds (**4a**, **5a** and **5c–g**) exhibited bactericidal activity against *M. tuberculosis* at the tested concentration, with MBCs of 34 μM (~12.5 μg/mL), 562 μM (~200 μg/mL), 266 μM (~100 μg/mL), 519 μM (~200 μg/mL), 488 μM (~200 μg/mL), 532 μM (~200 μg/mL) and 537 μM (~200 μg/mL), respectively. The remaining compounds did not show any bactericidal effects against *M. tuberculosis* H37Ra, even at the highest test concentration of 200 μg/mL.

The in vitro antituberculosis evaluation revealed that the compounds which contained substituted *para* methyl (**4a** and **5a**) in two series exhibited activity against *M. tuberculosis*. The result confirmed that methyl group substitution at the phenyl ring in pyrazoline analogues has a favorable effect, and a prominent improvement in inhibition was noticed [22]. However, it was found that compounds with a substituted *para* methoxy (**4b** and **5b**) in both series showed no inhibition against *M. tuberculosis* H37Ra, even at the highest tested concentration of 200 μg/mL. This is not surprising, as previous studies have reported that (OCH_3_) group substitution at the phenyl ring in pyrazoline analogues worsens the antituberculosis activity [23]. Moreover, it was discovered that all compounds (**5a** and **5c–g**) from the second series (2-(4-hydroxyphenyl)-2-oxoethan-1-ide derivatives), except for compound **5b**, exhibited moderate activity against *M. tuberculosis* H37Ra. These results confirmed that hydroxylphenyl substitution is necessary for antituberculosis activity [24].

### 2.3. Molecular Docking

**Figure 4:**
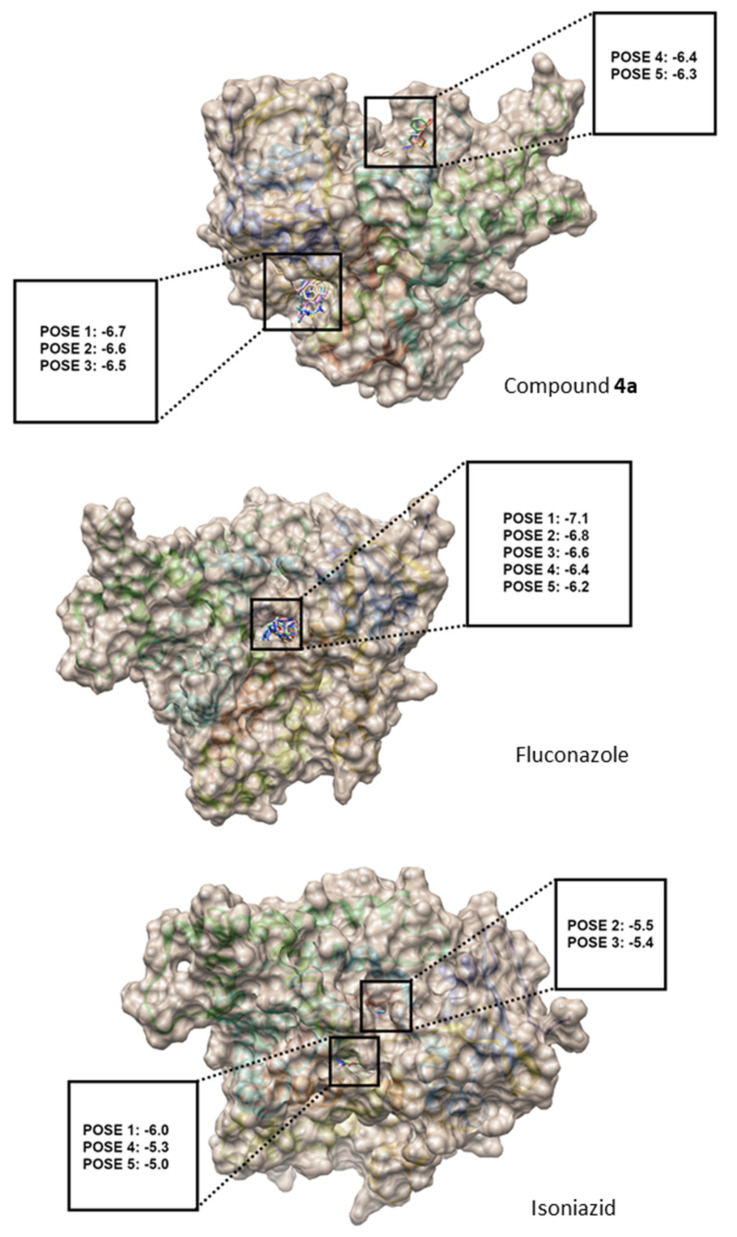
Active sites of CYP51 for binding of compound **4a** and controls (Fluconazole & Isonizid).

The free binding energy and interaction modes between compound **4a** and residues in the active site of cytochrome P450 14 alpha-sterol demethylase (CYP51) were identified by molecular docking studies using AutoDock Vina version 1.14. The crystal structure of CYP51 complexed with fluconazole (Protein Data Bank ID - PDB ID 1EA1) was used as a reference structure for the docking process [25]. The structure of isoniazid (PDB ID: 2VCF) was used as an experimental control to investigate molecular docking with CYP51. The docking results indicated binding energy between CYP51 and the control inhibitor (fluconazole) or the experimental control (isoniazid) or compound **4a**, ranging between −6.2 to −7.1, −6.0 to −5.0 and −6.3 to −6.7 respectively (Table 2). The active site(s) of CYP51 responsible for the binding of compound **4a** and the controls (isoniazid and fluconazole) is shown in Figure 4.

**Figure 5:**
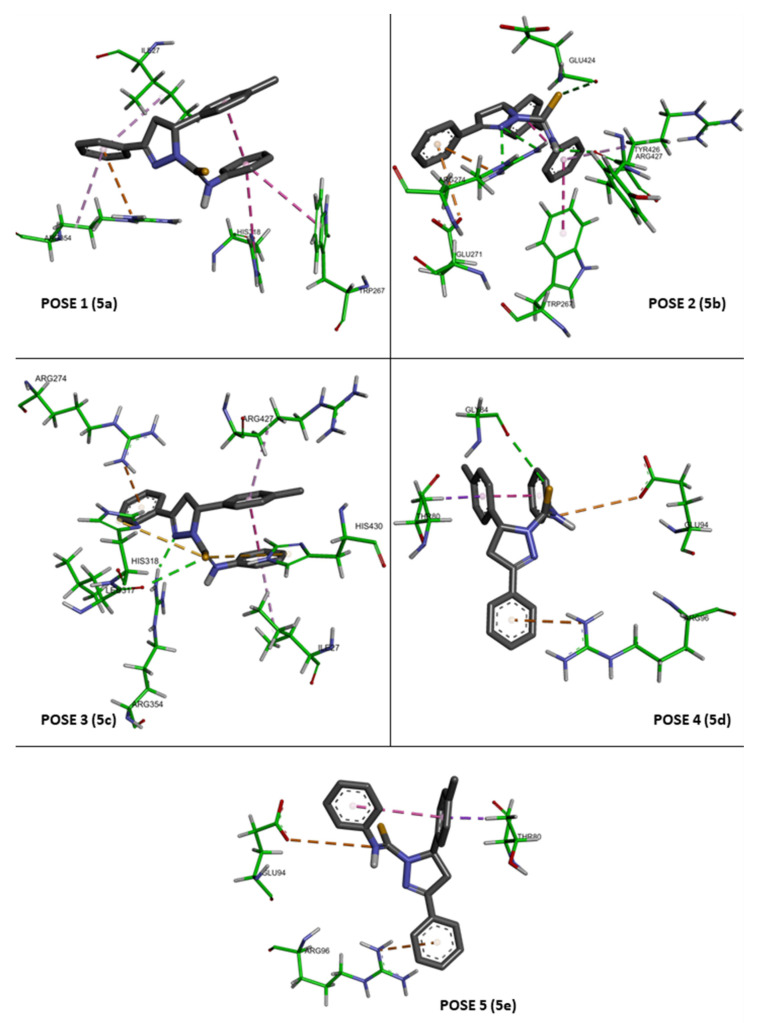
Three-dimensional binding modes of compound **4a** (pose 1–5) present at the active sites of CYP51. The interaction modes of the most potent pose in CYP51: Figure 5a orientation **4a** in pose 1, Figure 5b orientation **4a** in pose 2, Figure 5c orientation **4a** in pose 3, Figure 5d orientation **4a** in pose 4 and Figure 5e orientation **4a** in pose 5. Pi-cation/Pi-anion/attractive charge interactions (orange), Pi-sigma interaction (purple), Pi-sulfur (light brown), conventional hydrogen bond interaction (green), unfavorable interaction (red), Pi-Pi T-shaped interaction (pink).

The interaction modes of compound **4a** with CYP51 are shown in Figure 5a–d. These structures detail the interactions in the active sites that are listed in Table 3. The interaction modes of the most potent pose in CYP51 (Figure 5a) showed that all phenyl rings at position 1 and 5, including the thioamide group, interacted with ILE27, TRP267, HIS318 and ARG354 via hydrophobic interactions. The second most potent pose (Figure 5b) demonstrated that the pyrazoline ring and thioamide group interacted with ARG274, GLU424 and TYR426, respectively, via non-conventional hydrogen bonding. Five hydrophobic interactions were formed between the thioamide group with ARG274, and at the phenyl rings at position 1 and 3 with TRP267, GLU271, ARG274, and ARG427.

The third pose indicated the compound also docked inside active site 2 (Figure 5c) with residue thioamide and pyrazoline ring interacted with LEU317 and ARG354, respectively. Meanwhile, three hydrophobic interactions were occurred between phenyl rings with ILE27, ARG274, and ARG247. The remaining hydrophobic interactions were established between the thioamide group and HIS318, ARG354, and HIS430. In contrast, poses 4 and 5 showed that compound **4a** preferred to bind at active site 3 of CYP51, with established interactions between residues THR80 and ARG96 with the phenyl group, and GLU94 with the thioamide group (Figure 5d–e). Only one non-conventional hydrogen bond was formed between the thioamide group and GLY84 in pose 4 (Figure 5d).

## 3. Materials and Methods

### 3.1. Experimental

#### 3.1.1. Chemistry

All chemicals and reagents were obtained from Acros Organic (Geel, Belgium), QRec (Penang, Malaysia, Asia), Sigma-Aldrich Chemical Co. or Merck (Darmstadt, Germany). Thin-layer chromatography (TLC) was performed on alumina plates pre-coated with silica gel (Merck 60 F254). The progress of reactions was determined by the appearance of product and disappearance of reactant spots under UV radiation (λ_max_ = 254 nm, Muttenz, Switzerland), respectively. Melting points were determined on open capillary tubes and are uncorrected. All spectral data were obtained on the following instruments: infrared (IR) spectra were recorded on a Perkin-Elmer System FTIR-ATR spectrometer; 1D- and 2D-NMR spectra were recorded on a 500 FT-NMR Bruker Advance spectrometer (Bruker Bioscience, Billerica, MA, USA) in CD_3_COCD_3_, DMSO-d_6_, CDCl_3_ and tetramethylsilane (TMS) as internal standards. Chemical shifts are reported in part per million (δ-scale) and the coupling constants, *J*, are reported in Hertz (Hz). High resolution HRMS mass spectra were obtained from a Waters Xevo QTOF MS system.

#### 3.1.2. General Procedure for the Preparation of Chalcone Derivatives (**3a–g**) by the Claisen–Schmidt Condensation Reaction

Chalcone derivatives (**3a**–**g**) were synthesized following the method described in the literature. A mixture of acetophenone (**1**) (0.01 mol) and substituted benzaldehyde (**2a**–**g**) (0.015 mol) in methanol (10 mL) was refluxed in the presence of few drops of piperidine for 72 h [26,27]. The solution was kept in an ice bath until a solid was obtained, and the chalcone compounds were filtered, washed with cold water, dried and recrystallized from ethanol. The results were compared with the literature [28,29,30,31].

*(E)-3-(4-methylphenyl)-1-phenylprop-2-en-1-one* (**3a**). Pale yellow crystalline solid; yield: 83%; m.p.: 94–96 °C; FTIR (ATR) ν_max_ (cm^−1^): 3025 (C_sp_^2^–H), 2918 (C_sp_^3^–H), 1656 (C=O), 1595 (–CH=CH–), 1514 and 1449 (aromatic C=C); ^1^H-NMR (500MHz, CD_3_COCD_3_): δ_H_ 2.39 (3H, s, 4-CH_3_), 7.30 (2H, d, *J* = 8.0 Hz, H-3′ and H-5′), 7.58 (2H, t, *J =* 7.5 Hz, H-2″ and H-6″), 7.66 (1H, t, *J* = 7.5 Hz, H-4″), 7.75 (2H, t, *J* = 8.0 Hz, H-2′ and H-6′), 7.79 (1H, d, *J* = 15.5 Hz, H-2), 7.85 (1H, d, *J* = 15.5 Hz, H-3) and 8.16 (2H, d, *J* = 7.5 Hz, H-3″ and H-5′’); ^13^C-NMR (125 MHz; CD_3_COCD_3_): δ_C_ 20.6 (4-CH_3_), 121.0 (C-2), 128.4 (C-3″ and C-5″), 128.7 (C-2″ and C-6″), 128.7 (C-2′ and C-6′), 129.6 (C-3′ and C-5′), 132.4 (C-1′), 132.7 (C-4″), 138.4 (C-1″), 140.9 (C-4′), 144.1 (C-3) and 189.1 (1-C=O).

*(E)-3-(4-methoxyphenyl)-1-phenylprop-2-en-1-one* (**3b**). Yellow crystalline solid; yield: 74%; m.p.: 73–75 °C; FTIR (ATR) ν_max_ cm^−1^: 3018 (C_sp_^2^–H), 2842 (C_sp_^3^–H), 1656 (C=O), 1575 (–CH=CH–), 1510, 1445 (aromatic C=C), 1253 (C–O); ^1^H-NMR (500MHz; CD_3_COCD_3_): δ_H_ 3.88 (3H, s, 4-OCH_3_), 7.03 (2H, d, *J* = 8.5 Hz, H-3′ and H-5′), 7.57 (2H, t, *J* = 7.5 Hz, H-2″ and H-6″), 7.65 (1H, t, *J* = 7.5 Hz, H-4″), 7.75 (1H, d, *J* = 15.5 Hz, H-2), 7.80 (1H, d, *J* = 15.5 Hz, H-3), 7.83 (2H, d, *J* = 7.5 Hz, H-2′ and H-6′), and 8.15 (2H, d, *J* = 7.5 Hz, H-3″ and H-5″); ^13^C-NMR (125 MHz; CD_3_COCD_3_); δ_C_ 54.9 (4-OCH_3_), 114.4 (C-3′ and C-5′), 119.6 (C-2), 127.7 (C-1′), 128.3 (C-3′’ and C-5″), 128.6 (C-2′’ and C-6″), 130.5 (C-2′ and C-6′), 132.6 (C-4″), 138.6 (C-1″), 143.9 (C-3), 161.9 (C-4′) and 189.0 (1-C=O).

*(E)-3-(4-chlorophenyl)-1-phenylprop-2-en-1-one* (**3c**). Pale yellow crystalline solid; yield: 82%; m.p.: 113–115 °C; FTIR (ATR) ν_max_ cm^−1^: 3059 (C_sp_^2^–H), 2931 (C_sp_^3^–H), 1653 (C=O), 1591 (–C=C–), 1563, 1489 (aromatic C=C), 1221 (C–O), 823 (C–Cl); ^1^H-NMR (500 MHz, CD_3_COCD_3_): δ_H_ 7.52 (2H, d, *J* = 8.5 Hz, H-3′ and H-5′), 7.58 (2H, t, *J* = 8.0 Hz, H-2″ and H-6″), 7.68 (1H, t, *J* = 7.5 Hz, H-4″), 7.79 (1H, d, *J* = 16.0 Hz, H-2), 7.90 (2H, d, *J* = 8.5 Hz, H-2′ and H-6′), 7.93 (1H, d, *J* = 16.0 Hz, H-3), and 8.17 (2H, d, *J* = 7.5 Hz, H-3″ and H-5″); ^13^C-NMR (125 MHz, CD_3_COCD_3_); δ_C_ 122.8 (C-2), 128.5 (C-3″ and C-5″), 128.7 (C-2″ and C-6″), 129.1 (C-2′ and C-‘6), 130.2 (C-3′ and C-5′), 132.9 (C-4″), 134.0 (C-1′), 135.7 (C-4′), 138.1 (C-1″), 142.4 (C-3) and 188.9 (1-C =O).

*(E)-3-(4-dimethylaminophenyl)-1-phenylprop-2-en-1-one* (**3d**). Orange crystalline solid; yield: 57%; m.p.: 113–115 °C; FTIR (ATR) ν_max_ cm^−1^: 3053.1 (C_sp_^2^–H), 2909.8 (C_sp_^3^–H), 1646 (C=O), 1611 (–C=C–), 1511, 1457 (C=C aromatic), 1322 (C–N); ^1^H-NMR (500 MHz; CD_3_COCD_3_): δ_H_ 3.07 (6H, s, 4-CH_3_ and 5-CH_3_), 6.80 (2H, d, *J* =9.0 Hz, H-3′ and H-5′), 7.55 (2H, t, *J* =7.5 Hz, H-2″ and H-6″), 7.61 (1H, d, *J* = 7.5 Hz, H-4″), 7.62 (1H, d, *J* = 15.5 Hz, H-2), 7.69 (2H, d, *J* = 9.0 Hz, H-2′ and H-6′), 7.77 (1H, d, *J* = 15.5 Hz, H-3) and 8.12 (2H, d, *J* = 7.5 Hz, H-3″ and H-5″); ^13^C-NMR (125 MHz; CD_3_COCD_3_); δ_C_ 39.3 (4-CH_3_ and 5-CH_3_), 111.8 (C-3′ and C-5′), 116.3 (C-2), 122.6 (C-1′), 128.1 (C-3″ and C-5″), 128.5 (C-2″ and C-6′’), 130.5 (C-2′ and C-6′), 132.2 (C-4″), 139.1 (C-1″), 145.1 (C-3), 152.3 (C-4′) and 188.7 (1-C=O).

*(E)-3-(2,4-dichlorophenyl)-1-phenylprop-2-en-1-one* (**3e**). Yellow solid; yield: 52%; m.p.: 69–73 °C; FTIR (ATR) ν_max_ cm^−1^: 3064 (C_sp_^2^–H), 2931 (C_sp_^3^–H), 1652 (C=O), 1607 (–C=C–), 1514, 1475.1 (C=C aromatic); ^1^H-NMR (500 MHz, CD_3_COCD_3_): δ_H_ 7.49 (1H, dd, *J* = 2.0 Hz and 8.5 Hz, H-5′), 7.59 (2H, t, *J* = 8.0 Hz, H-2″ and H-6′’), 7.64 (1H, d, *J* = 2.0 Hz, H-3′), 7.69 (1H, d, *J* = 7.5 Hz, H-4″), 7.96 (1H, d, *J* = 16.0 Hz, H-2), 8.12 (1H, d, *J* = 15.5 Hz, H-3) and 8.18 (3H, t, *J* = 7.5 Hz, H-6′, H-3″ and H-5″); ^13^C-NMR (125 MHz, CD_3_COCD_3_); δ_C_ 125.2 (C-2), 127.9 (C-5′), 128.6 (C-3′’ and C-5″), 128.8 (C-2″ and C-6″), 129.4 (C-3′), 129.6 (C-6′), 132.0 (C-4′), 133.2 (C-4″), 135.6 (C-1′), 136.0 (C-2′), 137.6 (C-3), 137.8 (C-1′’) and 188.7 (1-C=O).

*(E)-3-(2-chlorophenyl)-1-phenylprop-2-en-1-one* (**3f**). Pale yellow crystalline solid; yield: 57%; m.p.: 45–48 °C; FTIR (ATR) ν_max_ cm^−1^: 3060 (C_sp_^2^–H), 2931 (C_sp_^3^–H), 1662 (C=O), 1603 (–C=C–), 1514, 1463 (C=C aromatic), 748 (C–Cl); ^1^H-NMR (500 MHz, CD_3_COCD_3_): δ_H_ 7.47 (2H, m, H-4′ and H-5′), 7.56 (1H, m, H-3′), 7.60 (2H, d, *J* = 7.5 Hz, H-2″ and H-6″), 7.69 (1H, d, *J* = 7.5 Hz, H-4″), 7.92 (1H, d, *J* = 15.5 Hz, H-2), 8.14 (1H, d, *J* = 7.5 Hz, H-6′), 8.19 (2H, d, *J* = 8.5 Hz, H-3″ and H-5″) and 8.20 (1H, d, *J* = 15.5 Hz, H-3); ^13^C-NMR (125 MHz, CD_3_COCD_3_); δ_C_ 124.7 (C-2), 127.6 (C-4′), 128.2 (C-6′), 128.5 (C-3″ and C-5″), 128.7 (C-2″ and C-6″), 130.1 (C-5′), 131.6 (C-3′), 133.0 (C-1′), 133.1 (C-4″), 134.9 (C-2′), 137.9 (C-1″), 139.0 (C-3) and 188.9 (1-C=O).

*(E)-3-(2-methoxyphenyl)-1-phenylprop-2-en-1-one* (**3g**). Yellow crystalline solid; yield: 62%; m.p.: 51–53 °C; FTIR (ATR) ν_max_ cm^−1^: 3019 (C_sp_^2^–H), 2948 (C_sp_^3^–H), 1659 (C=O), 1594 (–C=C–), 1513, 1439 (C=C aromatic), 1247 (C–O); ^1^H-NMR (500 MHz; CD_3_COCD_3_): δ_H_ 3.98 (3H, s, 4-OCH_3_), 7.05 (1H, t, *J* = 7.5 Hz, H-5′), 7.13 (1H, d, *J* = 8.5 Hz, H-3′), 7.64 (1H, m, H-4′), 7.58 (2H, t, *J* = 7.5 Hz, H-2″ and H-6″), 7.66 (1H, d, *J* = 7.5 Hz, H-4″), 7.88 (1H, d, *J* = 15.5 Hz, H-2), 7.90 (1H, t, *J* = 7.5 Hz, H-6′), 8.14 (2H, d, *J* = 8.5 Hz, H-3″ and H-5″) and 8.17 (1H, d, *J* = 15.5 Hz, H-3); ^13^C-NMR (125 MHz; CD_3_COCD_3_); δ_C_ 55.2 (4-OCH_3_), 111.5 (C-3′), 120.7 (C-5′), 122.1 (C-2), 123.6 (C-1′), 128.4 (C-3″ and C-5″), 128.6 (C-6′), 128.6 (C-2″ and C-6″), 132.0 (C-4′), 132.7 (C-4″), 138.5 (C-1″), 139.0 (C-3), 158.8 (C-2′) and 189.4 (1-C=O).

#### 3.1.3. General Procedure for the Preparation of the First Series of 3,5-disubstituted-4,5-dihydro-N-phenyl-1H-pyrazole-1-carbothioamide (**4a–g**)

The carbothioamide compounds (**4a–g**) were synthesized according to a previously reported method with slight modifications. The cyclo-condensation of chalcone derivatives (**3a–g**) (1 mmol) with 4-phenyl-3-thiosemicarbazide (1.5 mmol) was carried out in ethanol (6 mL) in the presence of NaOH (3 mmol) for 3 to 8 h [32]. The reaction progress was observed on a TLC plate. After the reaction was over, the reaction mixture was left at room temperature overnight. A solid was formed after crushed ice was added. The solid was filtered, washed with cold water, dried and recrystallized from ethanol. Please refer the Supplementary Data for full spectra.

*5-(4-methylphenyl)-N,3-diphenyl-4,5-dihydro-1H-pyrazole-1-carbothioamide* (**4a**). Cream-colored solid; yield: 26%; m.p.: 133–136 °C; FTIR (ATR) ν_max_ cm^−1^: 3291 (N–H), 3028 (C_sp_^2^–H), 2919 (C_sp_^3^–H), 1594 (C=N), 1520, 1449 (aromatic C=C), 1399 (C=S); ^1^H-NMR (500 MHz, CD_3_COCD_3_): δ_H_ 2.29 (3H, s, 14-CH_3_), 3.26 (1H, dd, *J* = 3.5 Hz and 18 Hz, 4α-CH), 4.05 (1H, dd, *J* = 11.5 Hz and 18 Hz, 4β-CH), 6.14 (1H, dd, *J* = 3.5 Hz and 11.5 Hz, 5-CH), 7.16 (5H, m, H-2′, H-3′, H-5′, H-6′, H-11), 7.33 (2H, t, *J* = 7.5 Hz, H-10 and H-12), 7.49 (3H, m, H-3″, H-4″ and H-5″), 7.72 (2H, m, H-9 and H-13), 7.98 (2H, dd, *J* = 2.0 Hz and 7.5 Hz, H-2″ and H-6″), 9.93 (1H, s, 7-NH); ^13^C NMR (125 MHz, CD_3_COCD_3_): δ_C_ 20.2 (14-CH_3_), 42.3 (4-CH_2_), 63.3 (5-CH), 124.3 (C-9 and C-13), 124.6 (C-11), 125.5 (C-2′ and C-6′), 127.2 (C-2″ and C-6″), 128.0 (C-10 and C-12), 128.7 (C-3″ and C-5″), 129.1 (C-3′ and C-5′), 130.7 (C-4″), 131.3 (C-1″), 136.5 (C-4′), 139.8 (C-8), 140.1 (C-1′), 155.3 (3-C=N), 174.2 (6-C=S); HRMS: 394.1156 [M + Na]^+^ {calcd. 394.1354 for C_23_H_21_N_3_SNa}.

*5-(4-methoxyphenyl)-N,3-diphenyl-4,5-dihydro-1H-pyrazole-1-carbothioamide* (**4b**). Pale yellow crystals; yield: 39%; m.p.: 173–175 °C; FTIR (ATR) ν_max_ cm^−1^: 3334 (N–H), 3058 (C_sp_^2^–H), 2842 (C_sp_^3^–H), 1592 (C=N), 1510, 1445 (aromatic C=C), 1396 (C=S), 1244 (C–O); ^1^H-NMR (500 MHz, CD_3_COCD_3_): δ_H_ 3.29 (1H, dd, *J* = 3.5 Hz and 18.0 Hz, 4α-CH), 3.78 (3H, s, 14-OCH_3_), 4.05 (1H, dd, *J* = 11.5 Hz and 18.0 Hz, 4β-CH), 6.13 (1H, dd, *J* = 3.5 Hz and 11.5 Hz, 5-CH), 6.89 (2H, d, *J* = 9.0 Hz, H-3′ and H-5′), 7.15 (1H, t, *J* = 7.5 Hz, H-11), 7.22 (2H, d, *J* = 8.5 Hz, H-2′ and H-6′), 7.33 (2H, t, *J* = 7.5 Hz, H-10 and H-12), 7.50 (3H, m, H-3″, H-4″ and H-5″), 7.73 (2H, d, *J* = 7.5 Hz, H-9 and H-13), 7.99 (2H, m, H-2″ and H-6″), 9.92 (1H, s, 7-NH); ^13^C NMR (125 MHz, CD_3_COCD_3_): δ_C_ 42.3 (4-CH_2_), 54.6 (14-OCH_3_), 63.1 (5-CH), 113.8 (C-3′ and C-5′), 124.3 (C-9 and C-13), 124.6 (C-11), 126.9 (C-2′ and C-6′), 127.2 (C-2″ and C-6″), 128.0 (C-10 and C-12), 128.7 (C-3″ and C-5″), 130.7 (C-4″), 131.4 (C-1″), 135.1 (C-1′), 139.8 (C-8), 155.3 (3-C=N), 158.9 (C-4′), 174.2 (6-C=S); HRMS: 410.1098 [M + Na]^+^ {calcd. 410.1303 for C_23_H_21_N_3_OSNa}.

*5-(4-chlorophenyl)-N,3-diphenyl-4,5-dihydro-1H-pyrazole-1-carbothioamide* (**4c**). Yellow solid; yield: 34%; m.p.: 150–153 °C; FTIR (ATR) ν_max_ cm^−1^: 3306 (N–H), 3034 (C_sp_^2^–H), 1594 (C=N), 1538, 1489 (aromatic C=C), 1380 (C=S); ^1^H-NMR (500 MHz, CD_3_COCD_3_): δ_H_ 3.33 (1H, dd, *J* = 3.5 Hz and 18.0 Hz, 4α-CH), 4.11 (1H, dd, *J* = 11.5 Hz and 18.0 Hz, 4β-CH), 6.18 (1H, dd, *J* = 3.5 Hz and 11.5 Hz, 5-CH), 7.16 (1H, t, *J* = 7.5 Hz, H-11), 7.35 (6H, m, H-2′, H-3′, H-5′, H-6′, H-10 and H-12), 7.50 (3H, m, H-3″, H-4″ and H-5″), 7.72 (2H, t, *J* = 7.5 Hz, H-9 and H-13), 7.99 (2H, d, *J* = 7.0 Hz, H-2″ and H-6″), 9.96 (1H, s, 7-NH); ^13^C-NMR (125 MHz, CD_3_COCD_3_): δ_C_ 42.1 (4-CH_2_), 63.0 (5-CH), 124.4 (C-9 and C-13), 124.7 (C-11), 127.2 (C-2′ and C-6′), 127.6 (C-2″ and C-6″), 128.1 (C-10 and C-12), 128.6 (C-3′ and C-5′), 128.7 (C-3″ and C-5″), 130.7 (C-4″), 131.2 (C-1″), 132.2 (C-4′), 139.7 (C-8), 142.0 (C-1′), 155.2 (3-C=N), 174.3 (6-C=S); HRMS: 392.0952 [M + H]^+^ {calcd. 392.0988 for (C_22_H_19_ClN_3_S}.

*5-(4-dimethylaminohenyl)-N,3-diphenyl-4,5-dihydro-1H-pyrazole-1-carbothioamide* (**4d**). Orange oil; yield: 12%; FTIR (ATR) ν_max_ cm^−1^: 3338 (N–H), 3031 (C_sp_^2^–H), 2921 (C_sp_^3^–H), 1595 (C=N), 1517, 1446 (aromatic C=C), 1395 (C=S), 1320 (C–N); ^1^H-NMR (500 MHz, CD_3_COCD_3_): δ_H_ 2.90(6H, s, 14-CH_3_ and 15-CH_3_), 3.09 (1H, dd, *J* = 3.5 Hz and 18.0 Hz, 4α-CH), 3.99 (1H, dd, *J* = 11.5 Hz and 18.0 Hz, 4β-CH), 6.08 (1H, dd, *J* = 3.5 Hz and 11.0 Hz, 5-CH), 6.69 (2H, d, *J* = 9.0 Hz, H-3′ and H-5′), 7.13 (3H, m, H-2′, H-6′ and H-11), 7.33 (2H, m, H-10 and H-12), 7.50 (3H, m, H-3″, H-4″ and H-5″), 7.74 (2H, m, H-9 and H-13), 7.98 (2H, m, H-2″ and H-6″), 9.88 (1H, s, 7-NH); ^13^C-NMR (125 MHz, CD_3_COCD_3_): δ_C_ 39.8 (14-CH_3_ and 15-CH_3_), 42.3 (4-CH_2_), 63.2 (5-CH), 112.4 (C-3′ and C-5′), 124.2 (C-9 and C-13), 124.5 (C-11), 126.6 (C-2′ and C-6′), 127.2 (C-2″ and C-6″), 128.0 (C-10 and C-12), 128.7 (C-3″ and C-5″), 130.6 (C-4″), 130.6 (C-1′), 131.5 (C-1″), 139.9 (C-8), 150.0 (C-4′), 155.3 (3-C=N), 174.1 (6-C=S); HRMS: 401.1848 [M + H]^+^ {calcd. 401.1800 for C_24_H_25_N_4_S}.

*5-(2,4-dichlorophenyl)-N,3-diphenyl-4,5-dihydro-1H-pyrazole-1-carbothioamide* (**4e**). Yellowish powder; yield: 15%; m.p.: 175–177 °C; FTIR (ATR) ν_max_ cm^−1^: 3345 (N–H), 3056 (C_sp_^2^–H), 2925 (C_sp_^3^–H), 1589 (C=N), 1510, 1447 (aromatic C=C), 1398 (C=S); ^1^H-NMR (500 MHz, CD_3_COCD_3_): δ_H_ 3.14 (1H, dd, *J* = 4.0 Hz and 18.0 Hz, 4α-CH), 4.03 (1H, dd, *J* = 12.0 Hz and 18.0 Hz, 4β-CH), 6.25 (1H, dd, *J* = 4.5 Hz and 12.0 Hz, 5-CH), 7.04 (2H, m, H-10 and H-12), 7.21 (3H, m, H-5′, H-6′ and H-11), 7.36 (3H, m, H-3″, H-4″ and H-5″), 7.40 (1H, s, H-3′), 7.59 (2H, dd, *J* = 1.0 Hz and 8.5 Hz, H-9 and H-13), 7.84 (2H, dd, *J* = 1.5 Hz and 8.0 Hz, H-2″ and H-6″), 9.88 (1H, s, 7-NH); ^13^C-NMR (125 MHz, CD_3_COCD_3_): δ_C_ 40.7 (4-CH_2_), 61.1 (5-CH), 124.4 (C-10 and C-12), 124.9 (C-11), 127.3 (C-2″ and C-6″), 127.6 (C-5′ and C-6′), 128.1 (C-9 and C-13), 128.7 (C-3″ and C-5″), 129.2 (C-3′), 130.8 (C-4″), 131.1 (C-1″), 132.0 (C-2′), 132.9 (C-4′), 139.0 (C-8), 139.6 (C-1′), 155.4 (3-C=N), 174.3 (6-C=S); HRMS: 426.0573 [M + H]^+^ {calcd. 426.0599 for (C_22_H_18_Cl_2_N_3_S}.

*5-(2-chlorophenyl)-N,3-diphenyl-4,5-dihydro-1H-pyrazole-1-carbothioamide* (**4f**). Yellow solid; yield: 34%; m.p.: 59–62 °C ([33]: 58–60 °C); FTIR (ATR) ν_max_ cm^−1^: 3310 (N–H), 3051 (C_sp_^2^–H), 2936 (C_sp_^3^–H), 1588 (C=N), 1516, 1447 (aromatic C=C), 1400 (C=S); ^1^H-NMR (500 MHz, CDCl_3_): δ_H_ 3.19 (1H, dd, *J* = 4.0 Hz and 18.0 Hz, 4α-CH), 3.99 (1H, dd, *J* = 11.5 Hz and 18.0 Hz, 4β-CH), 6.50 (1H, dd, *J* = 3.5 Hz and 12.0 Hz, 5-CH), 7.20 (5H, m, H-3′, H-4′, H-5′, H-6′ and H-11), 7.40 (2H, t, *J =* 7.5 Hz, H-10 and H-12), 7.46 (3H, m, H-3″, H-4″ and H-5″), 7.79 (2H, d, *J* = 7.5 Hz, H-9 and H-13), 7.83 (2H, m, H-2″ and H-6″), 9.37 (1H, s, 7-NH); ^13^C-NMR (125 MHz, CDCl_3_): δ_C_ 41.5 (4-CH_2_), 61.3 (5-CH), 124.2 (C-9 and C-13), 125.5 (C-11), 126.9 (C-2″ and C-6″), 127.2 (C-5′), 128.7 (C-10 and C-12), 128.7 (C-6′), 128.9 (C-3″ and C-5″), 129.4 (C-3′), 130.0 (C-4′), 130.6 (C-4″), 131.1 (C-2′), 131.3 (C-1″), 138.7 (C-1′), 138.8 (C-8), 155.4 (3-C=N), 174.1 (6-C=S); HRMS: 414.0771 [M + Na]^+^ {calcd. 414.0808 for C_22_H_18_ClN_3_SNa}.

*5-(2-methoxyphenyl)-N,3-diphenyl-4,5-dihydro-1H-pyrazole-1-carbothioamide* (**4g**). Yellow solid; yield: 22%; m.p.: 193–196 °C; FTIR (ATR) ν_max_ cm^−1^: 3359 (N–H), 3055 (C_sp_^2^–H), 2920 (C_sp_^3^–H), 1572 (C=N), 1520, 1445 (aromatic C=C), 1379 (C=S); ^1^H-NMR (500 MHz, CD_3_COCD_3_): δ_H_ 3.27 (1H, dd, *J* = 3.5 Hz and 18.0 Hz, 4α-CH), 3.82 (3H, s, 14-OCH_3_), 4.06 (1H, dd, *J* = 11.5 Hz and 18.0 Hz, 4β-CH), 6.15 (1H, dd, *J* = 3.5 Hz and 11.5 Hz, 5-CH), 7.16 (5H, q, *J* = 8.0 Hz and 17.5 Hz, H-3′, H-4′, H-5′, H-6′ and H-11), 7.33 (2H, t, *J* = 7.5 Hz, H-10 and H-12), 7.50 (3H, m, H-3″, H-4″ and H-5″), 7.73 (2H, d, *J* = 7.5 Hz, H-9 and H-13), 7.98 (2H, dd, *J* = 1.5 Hz and 8.0 Hz, H-2″ and H-6″), 9.93 (1H, s, 7-NH); ^13^C-NMR (125 MHz, CD_3_COCD_3_): δ_C_ 42.3 (4-CH_2_), 55.8 (14-OCH_3_), 63.3 (5-CH), 124.3 (C-9 and C-13), 124.6 (C-11), 125.6 (C-5′ and C-6′), 127.2 (C-2″ and C-6″), 128.0 (C-10 and C-12), 128.7 (C-3″ and C-5″), 129.1 (C-3′ and C-4′),130.7 (C-4″), 131.3 (C-1″), 136.5 (C-2′), 139.8 (C-8), 140.1 (C-1′), 155.3 (3-C=N), 174.2 (6-C=S); HRMS: 410.1319 [M + Na]^+^ {calcd. 410.1303 for C_23_H_21_N_3_OSNa}.

#### 3.1.4. General Procedure for the Preparation of (3,5-disubstituted-4,5-dihydro-1H-pyrazol-yl) (4-hydroxyphenyl)methanone (**5a–g**)

The new methanone compounds (**5a–g**) were synthesized by the cyclo-condensation of chalcone (**3a–g**) (1 mmol) with 4-hydroxybenzhydrazide (1.5 mmol) in ethanol (6 mL) in the presence of NaOH (3 mmol) for 3 to 8 h. The reaction progress was observed on a TLC plate. After the reaction was over, the reaction mixture was left at room temperature overnight. The reaction mixture was neutralized by adding 1% *v/v* of HCl and monitored by pH paper until the precipitate formed. A solid was formed after crushed ice was added. The solid was filtered, washed with cold water and dried. The products were purified by CC using silica gel with the eluent *n*-hexane:ethyl acetate to give the compounds **5a–g**. Please refer the Supplementary Data for full spectra.

*N-(4-hydroxyphenyl)(5-(4-methylphenyl)-3-phenyl-4,5-dihydro-1H-pyrazol-1-yl)methanone* (**5a**). Pale yellow powder; yield: 29%; m.p.: 224–228 °C; FTIR (ATR) ν_max_ cm^−1^: 3209 (O–H), 3055 (C_sp_^2^–H), 2923 (C_sp_^3^–H), 1740 (C=O), 1588 (C=N), 1511, 1439 (aromatic C=C), 1230 (C–O); ^1^H-NMR (500 MHz, DMSO-d_6_): δ_H_ 2.27 (3H, s, 14-CH_3_), 3.12 (1H, dd, *J* = 5.5 Hz and 18.0 Hz, 4α-CH), 3.87 (1H, dd, *J* = 12.0 Hz and 18.0 Hz, 4β-CH), 5.71 (1H, dd, *J* = 5.0 Hz and 11.5 Hz, 5-CH), 6.85 (2H, d, *J* = 9.0 Hz, H-8 and H-12), 7.16 (4H, m, H-2′, H-3′, H-5′ and H-6′), 7.47 (3H, m, H-3″, H-4″ and H-5″), 7.75 (2H, m, H-2″ and H-6″), 7.86 (2H, d, *J* = 8.5 Hz, H-9 and H-11), 10.04 (1H, s, 13-OH); ^13^C-NMR (125 MHz, DMSO-d_6_): δ_C_ 21.1 (14-CH_3_), 41.6 (4-CH_2_), 61.0 (5-CH), 115.0 (C-8 and C-12), 125.3 (C-7), 126.0 (C-2″ and C-6″), 127.1 (C-2′ and C-6′), 129.3 (C-3′ and C-5′), 129.7 (C-3″ and C-5″), 130.7 (C-4″), 131.7 (C-1″), 132.4 (C-9 and C-11), 136.8 (C-4′), 140.2 (C-1′), 155.9 (3-C=N), 160.4 (10-C-OH), 165.2 (6-C=O); HRMS: 357.1591 [M + H]^+^ {calcd. 357.1603 for C_23_H_21_N_2_O_2_}.

*N-(4-hydroxyphenyl)(5-(4-methoxyphenyl)-3-phenyl-4,5-dihydro-1H-pyrazol-1-yl)methanone* (**5b**). Pale yellow powder; yield: 30%; m.p.: 228–232 °C; FTIR (ATR) ν_max_ cm^−1^: 3211.5 (O–H), 3066 (C_sp_^2^–H), 2929 (C_sp_^3^–H), 1741 (C=O), 1605 (C=N), 1513, 1437 (aromatic C=C), 1236 (C–O); ^1^H-NMR (500 MHz, DMSO-d_6_): δ_H_ 3.14 (1H, dd, *J* = 5.0 Hz and 18.0 Hz, 4α-CH), 3.72 (3H, s, 14-OCH_3_), 3.86 (1H, dd, *J* = 12.0 Hz and 18.0 Hz, 4β-CH), 5.70 (1H, dd, *J* = 5.0 Hz and 11.5 Hz, 5-CH), 6.84 (2H, d, *J* = 9.0 Hz, H-8 and H-12), 6.90 (2H, d, *J* = 8.5 Hz, H-2′ and H-6′), 7.21 (2H, d, *J* = 8.5 Hz, H-3′ and H-5′), 7.47 (3H, m, H-3″, H-4″ and H-5″), 7.76 (2H, m, H-2″ and H-6″), 7.85 (2H, d, *J* = 9.0 Hz, H-9 and H-11), 10.03 (1H, s, 13-OH); ^13^C-NMR (125 MHz, DMSO-d_6_): δ_C_ 41.6 (4-CH_2_), 55.6 (14-OCH_3_), 60.7 (5-CH), 114.5 (C-2′ and C-6′), 114.9 (C-8 and C-12), 125.4 (C-7), 127.1 (C-2″ and C-6″), 127.4 (C-3′ and C-5′), 129.3 (C-3″ and C-5″), 130.7 (C-4″), 131.8 (C-1″), 132.4 (C-9 and C-11), 135.2 (C-1′), 155.0 (3-C=N), 158.9 (C-4′), 160.4 (10-C-OH), 165.2 (6-C=O); HRMS: 373.1583 [M + H]^+^ {calcd. 373.1552 for C_23_H_21_N_2_O_3_}.

*N-(4-hydroxyphenyl)(5-(4-chlorolphenyl)-3-phenyl-4,5-dihydro-1H-pyrazol-1-yl)methanone* (**5c**). Yellowish needles; yield: 16%; m.p.: 263–266 °C; FTIR (ATR) ν_max_ cm^−1^: 3255 (O–H), 3070 (C_sp_^2^–H), 2926 (C_sp_^3^–H), 1738 (C=O), 1572 (C=N), 1516, 1438 (aromatic C=C), 1238 (C–O); ^1^H-NMR (δ/ppm, 500 MHz, DMSO-d_6_): δ_H_ 3.16 (1H, dd, *J* = 5.5 Hz and 18.0 Hz, 4α-CH), 3.89 (1H, dd, *J* = 12.0 Hz and 18.0 Hz, 4β-CH), 5.76 (1H, dd, *J* = 5.0 Hz and 11.5 Hz, 5-CH), 6.85 (2H, d, *J* = 9.0 Hz, H-8 and H-12), 7.32 (2H, d, *J* = 8.5 Hz, H-2′ and H-6′), 7.41 (2H, d, *J* = 8.5 Hz, H-3′ and H-5′), 7.47 (3H, m, H-3″, H-4″ and H-5″), 7.75 (2H, m, H-2″ and H-6″), 7.87 (2H, d, *J* = 8.5 Hz, H-9 and H-11), 10.07 (1H, s, 13-OH); ^13^C-NMR (δ/ppm, 125 MHz, DMSO-d_6_): δ_C_ 41.4 (4-CH_2_), 60.7 (5-CH), 114.9 (C-8 and C-12), 125.1 (C-7), 127.2 (C-2″ and C-6″), 128.1 (C-2′ and C-6′), 129.1 (C-3′ and C-5′), 129.3 (C-3″ and C-5″), 130.8 (C-4″), 131.6 (C-1″), 132.2 (C-4′), 132.5 (C-9 and C-11), 142.1 (C-1′), 155.1 (3-C=N), 160.5 (10-C-OH), 165.3 (6-C=O); HRMS: 377.1030 [M + H]^+^ {calcd. 376.0979 for C_22_H_17_ClN_2_O_2_}.

*N-(4-hydroxyphenyl)(5-(4-dimethylaminophenyl)-3-phenyl-4,5-dihydro-1H-pyrazol-1-yl) methanone* (**5d**). Brownish solid; yield: 9%; m.p.: 236–240 °C; FTIR (ATR) ν_max_ cm^−1^: 3241.4 (O–H), 3065.5 (C_sp_^2^–H), 2929 (C_sp_^3^–H), 1720 (C=O), 1604 (C=N), 1511, 1423 (aromatic C=C), 1339 (C–N), 1226 (C–O). ^1^H-NMR (500 MHz, CDCl_3_): δ_H_ 2.77 (6H, s, 14-CH_3_ and 15-CH_3_), 3.09 (1H, dd, *J* = 5.0 Hz and 17.5 Hz, 4α-CH), 3.62 (1H, dd, *J* = 11.5 Hz and 18.0 Hz, 4β-CH), 5.65 (1H, dd, *J* = 5.0 Hz and 11.5 Hz, 5-CH), 6.64 (2H, d, *J* = 8.5 Hz, H-8 and H-12), 6.71 (2H, d, *J* = 9.0 Hz, H-2′ and H-6′), 7.12 (2H, d, *J* = 8.5 Hz, H-3′ and H-5′), 7.32 (3H, m, H-3″, H-4″ and H-5″), 7.63 (2H, m, H-2″ and H-6″), 7.85 (2H, d, *J* = 7.5 Hz, H-9 and H-11); ^13^C NMR (125 MHz, CDCl_3_): δ_C_ 41.1 (14-CH_3_ and 15-CH_3_), 41.4 (4-CH_2_), 61.1 (5-CH), 113.6 (C-3′ and C-5′), 114.7 (C-8 and C-12), 125.4 (C-7), 126.8 (C-2′ and C-6′), 126.9 (C-2″ and C-6″), 128.7 (C-3″ and C-5″), 130.4 (C-4″), 130.6 (C-1′), 131.5 (C-1″), 132.5 (C-9 and C-11), 149.5(C-4′), 155.1 (3-C=N), 159.4 (10-C-OH), 166.4 (6-C=O); HRMS: 386.1891 [M + H]^+^ {calcd. 386.1869 for C_24_H_24_N_3_O_2_}.

*N-(4-hydroxyphenyl)(5-(2,4-dichlorolphenyl)-3-phenyl-4,5-dihydro-1H-pyrazol-1-yl)methanone* (**5e**). Pale yellow powder; yield: 17%; m.p.: 237–241 °C; FTIR (ATR) ν_max_ cm^−1^: 3137 (O–H), 3066 (C_sp_^2^–H), 2926 (C_sp_^3^–H), 1737 (C=O), 1618 (C=N), 1573, 1427 (aromatic C=C), 1244 (C–O); ^1^H-NMR (500 MHz, DMSO-d_6_): δ_H_ 3.13 (1H, dd, *J* = 6.0 Hz and 18.0 Hz, 4α-CH), 3.96 (1H, dd, *J* = 12.0 Hz and 18.0 Hz, 4β-CH), 5.95 (1H, dd, *J* = 5.5 Hz and 12.0 Hz, 5-CH), 6.87 (2H, d, *J* = 8.5 Hz, H-8 and H-12), 7.22 (1H, d, *J* = 8.0 Hz, H-6′), 7.40 (1H, dd, *J* = 2.5 Hz and 8.5 Hz, H-5′), 7.47 (3H, m, H-3″, H-4″ and H-5″), 7.69 (1H, d, *J* = 2.5 Hz, H-3′), 7.75 (2H, m, H-2″ and H-6″), 7.91 (2H, d, *J* = 8.5 Hz, H-9 and H-11), 10.11 (1H, s, 13-OH); ^13^C NMR (125 MHz, DMSO-d_6_): δ_C_ 40.1 (4-CH_2_), 58.8 (5-CH), 114.9 (C-8 and C-12), 124.7 (C-7), 127.2 (C-2″ and C-6″), 128.4 (C-5′ and C-6′), 129.3 (C-3″ and C-5″), 129.6 (C-3′), 130.9 (C-4″), 131.5 (C-1″), 132.4 (C-2′), 132.6 (C-9 and C-11), 133.0 (C-4′), 138.9 (C-1′), 155.3 (3-C=N), 160.7 (10-C-OH), 165.2 (6-C=O); HRMS: 411.0694 [M + H]^+^ {calcd. 411.0667 for C_22_H_17_Cl_2_N_2_O_2_}.

*N-(4-hydroxyphenyl)(5-(2-chlorolphenyl)-3-phenyl-4,5-dihydro-1H-pyrazol-1-yl)methanone* (**5f**). Pale yellow powder; yield: 15%; m.p.: 216–219 °C; FTIR (ATR) ν_max_ cm^−1^: 3125 (O–H), 3066 (C_sp_^2^–H), 2928 (C_sp_^3^–H), 1720 (C=O), 1605 (C=N), 1514, 1431 (aromatic C=C), 1240 (C–O); ^1^H-NMR (500 MHz, DMSO-d_6_): δ_H_ 3.05 (1H, dd, *J* = 5.5 Hz and 18.0 Hz, 4α-CH), 3.96 (1H, dd, *J* = 12.0 Hz and 18.0 Hz, 4β-CH), 5.96 (1H, dd, *J* = 5.5 Hz and 12.0 Hz, 5-CH), 6.86 (2H, d, *J* = 9.0 Hz, H-8 and H-12), 7.18 (1H, m, H-5′), 7.30 (2H, m, H-3′ and H-6′), 7.44 (3H, m, H-3″, H-4″ and H-5″), 7.50 (1H, m, H-4′), 7.72 (2H, m, H-2″ and H-6″), 7.89 (2H, d, *J* = 8.5 Hz, H-9 and H-11), 10.27 (1H, s, 13-OH); ^13^C NMR (125 MHz, DMSO-d_6_): δ_C_ 40.4 (4-CH_2_), 59.1 (5-CH), 115.0 (C-8 and C-12), 124.9 (C-7), 127.1 (C-2″, C-6″ and C-5′), 128.2 (C-6′), 129.3 (C-3″ and C-5″), 129.5 (C-3′), 130.3 (C-4′), 130.9 (C-4″), 131.4 (C-1″ and C-2′), 132.5 (C-9 and C-11), 139.6 (C-1′), 155.3 (3-C=N), 160.5 (10-C-OH), 165.4 (6-C=O); HRMS: 377.1011 [M + H]^+^ {calcd. 377.1057 for C_22_H_18_ClN_2_O_2_}.

*N-(4-hydroxyphenyl)(5-(2-methoxyphenyl)-3-phenyl-4,5-dihydro-1H-pyrazol-1-yl)methanone* (**5g**). Pale yellow powder; yield: 22%; m.p.: 220–223 °C; FTIR (ATR) ν_max_ cm^−1^: 3198 (O–H), 3029 (C_sp_^2^–H), 2925 (C_sp_^3^–H), 1738 (C=O), 1603 (C=N), 1513, 1429 (aromatic C=C), 1245 (C–O); ^1^H-NMR (500 MHz, DMSO-d_6_): δ_H_ 3.00 (1H, dd, *J* = 5.0 Hz and 18.0 Hz, 4α-CH), 3.84 (1H, dd, *J* = 10.0 Hz and 19.0 Hz, 4β-CH), 3.85 (3H, s, 14-OCH_3_), 5.89 (1H, dd, *J* = 4.5 Hz and 12.0 Hz, 5-CH), 6.87 (3H, m, H-5′, H-8 and H-12), 7.05 (2H, m, H-3′ and H-6′), 7.26 (1H, m, H-4′), 7.45 (3H, m, H-3″, H-4″ and H-5″), 7.74 (2H, m, H-2″ and H-6″), 7.89 (2H, d, *J* = 9.0 Hz, H-9 and H-11), 10.09 (1H, s, 13-OH); ^13^C NMR (125 MHz, DMSO-d_6_): δ_C_ 40.1 (4-CH_2_), 56.1 (14-OCH_3_), 56.9 (5-CH), 111.9 (C-6′), 114.9 (C-8 and C-12), 120.9 (C-5′), 125.4 (C-7), 125.9 (C-3′), 127.1 (C-2″ and C-6″), 128.9 (C-4′), 129.3 (C-3″ and C-5″), 131.0 (C-1′), 130.7 (C-4″), 131.8 (C-1″), 132.4 (C-9 and C-11), 155.5 (3-C=N), 156.4 (C-2′), 160.4 (10-C-OH), 165.1 (6-C=O); HRMS: 373.1599 [M + H]^+^ {calcd. 373.1552 for C_23_H_21_N_2_O_3_}.

#### 3.1.5. Screening of Anti-Tuberculosis Activity against *M. tuberculosis* H37Ra

The tetrazolium microplate assay (TEMA) method was performed to evaluate the anti-mycobacterial activity of the derivatives as described by Caviedes et al. with minor modifications [34]. The assay was performed in 96-well plates in duplicate and at least three times independently. The derivatives were dissolved in DMSO and serially diluted to the desired concentration in complete Middlebrook 7H9 media enriched with albumin dextrose catalase supplement to reduce the DMSO concentration below 1%. DMSO at this concentration did not inhibit the growth of *M. tuberculosis* H37Ra (unreported data). Briefly, 200 μL of sterile distilled water was added to the outer wells of the microplate and 100 µL of Middlebrook 7H9-ADC media into the wells in columns C to G, rows 2 to 11. Then, 100 μL of working solution containing the compounds was added into the wells in columns B and C, rows 2 to 11, in duplicate. A two-fold serial dilution of the compounds was made by transferring 100 μL from the wells in column C to column D, the content was mixed well, and the dilutions were continued until column G, where 100 μL of the excess medium from the wells in column G was discarded. Log phase *M. tuberculosis* H37Ra at a density of (~1.5 × 107 CFU/mL) was added and incubated at 37 °C with 5% CO_2_ for 5 days. On day 5, 50 μL of tetrazolium reagent mixture was added to all wells, and the plates were re-incubated for 24 h. The results were read visually the following day. The MIC is defined as the lowest drug concentration that prevented the color change from yellow to purple. A small volume of the culture from the 96-well plate was transferred into Middlebrook 7H10 agar media and the plates were incubated at 37 °C with 5% CO_2_ for 28 days. The MBC is defined as the lowest concentration of compound that did not show any bacterial colony growth.

#### 3.1.6. Molecular Docking

The three-dimensional coordinates of the reference structure, cytochrome P45014 alpha-sterol demethylase (CYP51) complexed with fluconazole (PDB ID: 1EA1), and the structure of the experimental control, isoniazid (PDB ID: 2VCF), were fetched by ID from the Protein Data Bank (PDB) database in UCSF Chimera version 1.14 [35]. Before docking, the crystal PDBs were processed using the Dock Prep tool, starting with the removal of water molecules and unrelated hetam (i.e., refer to any ions molecules and any atoms that not belong to the protein) followed by separation of the receptor and inhibitor from the complexes into individual structures and finally the minimization of individual structures by steepest descent steps [36]. The compound **4a** was built using ChemDraw and subsequently converted from cdx format to PDB.

To recognize the binding sites in CYP51, the grid size was set to −18, −2.6 and 63 along the X-, Y- and Z-axes, respectively, with a 0.375 Å grid spacing. The grid center along the X-, Y- and Z-axes was set to 76, 72, and 83 Å. The AutoDock Vina tool was then used to calculate possible bindings and energies [37]. All poses were combined using UCSF Chimera version 1.14 and further labelled by the GNU Image Manipulation program. The interaction modes between CYP51 and compound **4** for each pose were further analyzed using Biovia Discovery Studio Visualizer Client 2020 (Dassault Systèmes BIOVIA, Discovery Studio Modeling Environment, Release 2017, San Diego: Dassault Systèmes, 2016).

## 4. Conclusions

In conclusion, two series of pyrazoline derivatives (**4a**–**g** and **5a**–**g**) were prepared by cyclocondensation reaction between chalcone derivatives (**3a–g**) with a different type of 4-phenyl-3-thiosemicarbazide/4-hydroxybenzhydrazide in the presence of NaOH as a catalyst. Nine out of fourteen new compounds synthesized from two series of experiments were elucidated for structure using FTIR, 1D-NMR, 2D-NMR and HRMS. All seven synthesized compounds (**5a–g**) from the second series are new compounds. The synthesized compounds were evaluated for their antituberculosis activity by in vitro study against *Mycobacterium tuberculosis* H37Ra. The tested compounds displayed some degree of inhibition, with MIC values ranging from 17 μM to 535 μM. Significant activity was found for compound **4a**, which had the lowest MIC value of 17 μM. Meanwhile, pyrazoline derivatives (**5a**, **5c**, **5d**, **5e**, **5f** and **5g**), except for **5b** from the second series, exhibited moderate activity against *M. tuberculosis* with MICs of 70 μM, 66 μM, 65 μM, 61 μM, 66 μM and 134 μM, respectively. In the MBC assay, compound **4a** showed the strongest killing effect towards *M. tuberculosis* H37Ra with a value of 34 μM. Molecular docking studies demonstrated that compound **4a** has the best binding capability towards the cytochrome P450 14 alpha-sterol demethylase (CYP51) complex.

## Figures and Tables

**Table 1:** In vitro anti-tuberculosis activities of pyrazoline derivatives (**4a–g** and **5a–g**) against *Mycobacterium tuberculosis* H37Ra.

Compounds	MIC		MBC	
	(μg/mL)	(μM)	(μg/mL)	(μM)
**4a**	6.25	17	12.5	34
**4b**	>200	>517	>200	>517
**4c**	200	511	>200	>511 (NC)
**4d**	>200	>500	>200	>500
**4e**	200	471	>200	>471 (NC)
**4f**	200	511	>200	>511 (NC)
**4g**	>200	>517	>200	>517
**5a**	25	70	200	562
**5b**	>200	>535	>200	>535
**5c**	25	66	100	266
**5d**	25	65	200	519
**5e**	25	61	200	488
**5f**	25	66	200	532
**5g**	50	134	200	537
Isoniazid (Control)	0.625	5	0.625	5

Results are from three independent experiments performed in duplicate. NC = no bactericidal effect even at the highest test concentration.

**Table 2:** Binding energy between cytochrome P450 14 alpha-sterol demethylase (CYP51) with compound **4a** and controls (fluconazole and isoniazid) for all poses.

Enzyme	Compound	Pose	Binding
CYP51 (PDB 1EA1)	**4a**	1	−6.7
		2	−6.6
		3	−6.5
		4	−6.4
		5	−6.3
		1	−7.1
		2	−6.8
	Fluconazole	3	−6.6
		4	−6.4
		5	−6.2
	Isoniazid	1	−6.0
		2	−5.5
		3	−5.4
		4	−5.3
		5	−5.0

**Table 3:** Binding interactions between compound **4a** with CYP51 (poses 1 to 5).

Enzyme + Compound	Active Site	Pose	Interacting Unit of Compounds	Protein Residue	Type of Interaction
CYP51 + Compound **4a**	2	1	Phenyl		Pi–Alkyl
			Phenyl	ILE27	Pi–Pi T-shaped
			Phenyl	TRP267	Pi–Pi T-shaped
			Phenyl	HIS318	Pi–Cation
			Thioamide	ARG354	Unfavorable
					Unfavorable
	2	2	Phenyl	TRP267	Pi–Pi Stacked
			Phenyl	GLU271	Pi–Anion
			Pyrazoline ring	ARG274	H-bond
			Phenyl		Pi–Cation
			Thioamide		Unfavorable
					Unfavorable
			Thioamide	GLU424	H-bond
			Thioamide	TYR426	H-bond
			Phenyl	ARG427	Pi–Alkyl
	2	3	Phenyl	ILE27	Pi–Alkyl
			Phenyl	ARG274	Pi–Cation
			Thioamide	LEU317	H-bond
			Thioamide	HIS318	Pi–Sulfur
			Pyrazoline ring	ARG354	H-bond
			Thioamide		Unfavorable
			Phenyl	ARG427	Pi–Alkyl
			Thioamide	HIS430	Pi–Sulfur
	3	4	Phenyl	THR80	Pi–Sigma
			Thioamide	GLY84	H-bond
			Thioamide	GLU94	Attractive charge
			Phenyl	ARG96	Pi–Cation
	3	5	Phenyl	THR80	Pi–Sigma
			Thioamide	GLU94	Attractive charge
			Phenyl	ARG96	Pi–Cation

## Data Availability

Data is contained within the article or supplementary material. The data presented in this study are available in this article or supplementary material.

## Supplementary Materials

The following are available online. Figures S1–S80: spectrums for compounds **4a**–**5g**.

## References

[1] Knorr L. (1883). Einwirkung von acetessigester auf phenylhydrazin. Eur. J. Inorg. Chem..

[2] Kumar G., Tanwer O., Kumar J., Akhter M., Sharma S., Pillai C.R., Alam M.M., Zama M.S. (2018). Pyrazole-pyrazoline as promising novel antimalarial agents: A mechanistics study. Eur. J. Med. Chem..

[3] Vineet M., Seema P., Rajendra N., Devashis M., Kirpa S. (2002). Substituted pyrazolines and their cardiovascular activity. Indian J. Chem. Sect. B.

[4] Lv P.C., Li D.D., Li Q.S., Lu X., Xiao Z.P., Zhu H.L. (2011). Synthesis, molecular docking and evaluation of thiazolyl-pyrazoline derivatives as EGFR TK inhibitors and potential anticancer agents. Bioorg. Med. Chem. Lett..

[5] Abid M., Bhat A.R., Athar F., Azam A. (2009). Synthesis, spectral studies and antiamoebic activity of new 1-N-substituted thiocarbamoyl-3-phenyl-2-pyrazolines. Eur. J. Med. Chem..

[6] Abdel-Wahad B.F., Abdel-Aziz H.A., Ahmed E.M. (2009). Synthesis and antimicrobial evaluation of 1-(benzofuran-2-yl)-4-nitro-3- arylbutan-1-ones and 3-(benzofuran-2-yl)-4,5-dihydro-5-aryl-1-[4-(aryl)-1, 3-thiazol-2-yl]-1H-pyrazoles. Eur. J. Med. Chem..

[7] Revanasiddappa B.C., Jisha M.S., Kumar M.V., Kumar H. (2018). Synthesis, antibacterial and antifungal evaluation of novel pyrazoline derivatives. Dhaka Univ. J. Pharm. Sci..

[8] Ismaeil Z.H., Soliman F.M.A., Abd-El Monem S.H. (2011). Synthesis, Antimicrobial and Antitumor Activity of Some 3, 5-Diaryl and 1, 3, 5-Triaryl-2-Pyrazoline Derivatives. J. Am. Sci..

[9] Nayak B.V., Ciftci-Yabanoglu S., Jadav S.S., Jagrat M., Sinha B.N., Ucar G., Jayaprakash V. (2013). Monoamine oxidase inhibitory activity of 3,5-biaryl-4,5-dihydro-1H- pyrazole-1-carboxylate derivatives. J. Med. Chem..

[10] Upadhyay S., Tripathi A.C., Paliwal S., Saraf S.K. (2017). 2-pyrazoline derivatives in neuropharmacology: Synthesis, adme prediction, molecular docking and in vivo biological evaluation. EXCLI.

[11] Ahsan M.J., Samy J.G., Soni S., Jain N., Kumar L., Sharma L.K., Yadav H., Saini L., Kalyansing R.G., Devenda N.S. (2011). Discovery of novel antitubercular 3a,4-dihydro-3H-indeno [1,2-c]pyrazole-2- carboxamide/carbothioamide analogues. Bioorg. Med. Chem. Lett..

[12] Palleapati K., Kancharlapalli V.R., Shaik A.B. (2019). Synthesis, characterization and antitubercular evaluation of some new isoxazole appended 1-carboxamido-4,5-dihydro-1H-pyrazoles. J. Res. Pharm..

[13] Chirke S.S., Krishna J.S., Rathod B.B., Bonam S.R., Khedkar V.M., Rao B.V., Sampath Kumar H.M., Shetty P.R. (2017). Synthesis of Triazole Derivatives of 9-Ethyl-9H-carbazole and Dibenzo[b,d]furan and Evaluation of Their Antimycobacterial and Immunomodulatory Activity. ChemistrySelect.

[14] Nustrat B., Siddiqui N., Sahu M., Naim M.J., Yar M.S., Ali R., Alam O. (2019). Anticonvulsant evaluation of 2-pyrazolines carrying napthyl moiety: An insight into synthesis and molecular docking study. Braz. J. Pharm. Sci..

[15] Bhandari S., Tripathi A.C., Saraf S.K. (2013). Novel 2-pyrazoline derivatives as potential anticonvulsant agents. Med. Chem. Res..

[16] Ozdemir Z., Kandilci H.B., Gumusel B., Calis U., Bilgin A.A. (2007). Synthesis and studies on antidepressant and anticonvulsant activities of some 3-(2-furyl)-pyrazoline derivatives. Eur. J. Med. Chem..

[17] World Health Organization (2015). 2015 Global Tuberculosis Report.

[18] World Health Organization (2019). 2019 Global Tuberculosis Report.

[19] Malaysia Health Ministry Official (2019). Malaysia’s High TB Mortality Rate Preventable. https://www.malaymail.com/news/malaysia/2019/12/16/health-ministry-official-tb-mortality-rate-high-in-malaysia-due-to-late-tre/1819581.

[20] Heinrichs M., May R., Heider F., Reimers T., B Sy S., Peloquin C., Derendorf H. (2018). Mycobacterium tuberculosis Strains H37ra and H37rv have equivalent minimum inhibitory concentrations to most antituberculosis drugs. Int. J. Mycobacteriol..

[21] McEvoy (2014). Characterizing carbonyls with infrared spectroscopy: An introductory chemistry experiment in a molecular bioscience program. J. Chem. Educ..

[22] Jadhav S.B., Fatema S., Sanap G., Farooqui M. (2018). Antitubercular activity and synergistic study of novel pyrazole derivatives. J. Heterocycl. Chem..

[23] Ali M.A., Shaharyar M., Siddiqui A.A. (2007). Synthesis, structural activity relationship and anti-tubercular activity of novel pyrazoline derivatives. Eur. J. Med. Chem..

[24] Gupta S.P.B.N., Pramanik S.K., Upmanyu N. (2009). Pyrazolines as promising medicinal agents. Int. J. Chem. Sci..

[25] Podust L.M., Poulos T.L., Waterman M.R. (2001). Crystal structure of cytochrome P450 14 alpha-sterol demethylase (CYP51) from Mycobacterium tuberculosis in complex with azole inhibitors. Proc. Natl. Acad. Sci. USA..

[26] Bhasker N., Prashanthi N., Subba Reddy B.V. (2015). Piperidine mediated synthesis of new series of prenyloxy chalcones, flavanones and comparative cytotoxic study. Der Pharm. Lett..

[27] Venkatesan P., Sumathi S. (2010). Piperidine Mediated Synthesis of N-Heterocyclic Chalcones and Their Antibacterial Activity. J. Heterocycl. Chem..

[28] Kumar S., Lamba M., Makrandi J.K. (2008). An efficient green procedure for the synthesis of chalcones using C-200 as solid support under grinding conditions. Green Chem. Lett. Rev..

[29] Supuran C.T., Popescu A., Ilisiu M., Costandache A., Banciu M.D. (1996). Carbonic anhydrase inhibitors. Part 36”. Inhibition of isozymes I and II with Schiff bases derived from chalkones and aromatic/heterocyclic sulfonamides. Eur. J. Med. Chem..

[30] Davey W., Gwilt J.R. (1957). Chalcones and related compounds. Part I. Preparation of nitro-, amino-, and halogeno-chalcones. J. Chem. Soc..

[31] Sashidhara K.V., Rosaiah J.N., Kumar A. (2009). Iodine-catalyzed mild and efficient method for the synthesis of chalcones. Syn. Commun..

[32] Beyhan N., Kocyigit-Kaymakcioglu B., Gümrü S., Aricioglu F. (2017). Synthesis and anticonvulsant activity of some 2-pyrazolines derived from chalcones. Arab. J. Chem..

[33] Patel V.I., Patel B. (2010). Synthesis and study of some pyrazole derivatives as anti-tubercular agent. Int. J. Pharma Bio Sci..

[34] Caviedes L., Delgado J., Gilman R.H. (2002). Tetrazolium Microplate Assay as a Rapid and Inexpensive Colometric Method for Determination of Antibiotic Susceptibility of Mycobacterium tuberculosis. J. Clin. Microbiol..

[35] Pettersen E.F., Goddard T.D., Huang C.C., Couch G.S., Greenblatt D.M., Meng E.C., Ferrin T.E. (2004). UCSF Chimera—A visualization system for exploratory research and analysis. J. Comput. Chem..

[36] Wang J., Wang W., Kollman P.A., Case D.A. (2006). Automatic atom type and bond type perception in molecular mechanical calculation. J. Mol. Graph. Model..

[37] Ddd Huang C.C., Meng E.C., Morris J.H., Pettersen E.F., Ferrin T.E. (2014). Enhancing UCSF Chimera through web services. Nucleic Acid Res..

