# Supplementary material for: Synthesis, Characterization and Biological Evaluation of New 3,5-Disubstituted-Pyrazoline Derivatives as Potential Anti-Mycobacterium tuberculosis H37Ra Compounds
Source: Molecules. 2021 Apr 5;26(7):2081. doi: 10.3390/molecules26072081 (PMC8038544; doi:10.3390/molecules26072081)
Supplement: Supplementary file 1 [file molecules-26-02081-s001.pdf]

Supporting information

Synthesis, Characterisation and Biological Evaluation of New 3,5-Disubstituted-Pyrazoline Derivatives as Potential Anti-*Mycobacterium tuberculosis* H37Ra.

Kok Tong Wong, Hasnah Osman, Thaigarajan Parumasivam, Unang Supratman, Mohammad Tasyriq Che Omar and Mohamad Nurul Azmi*

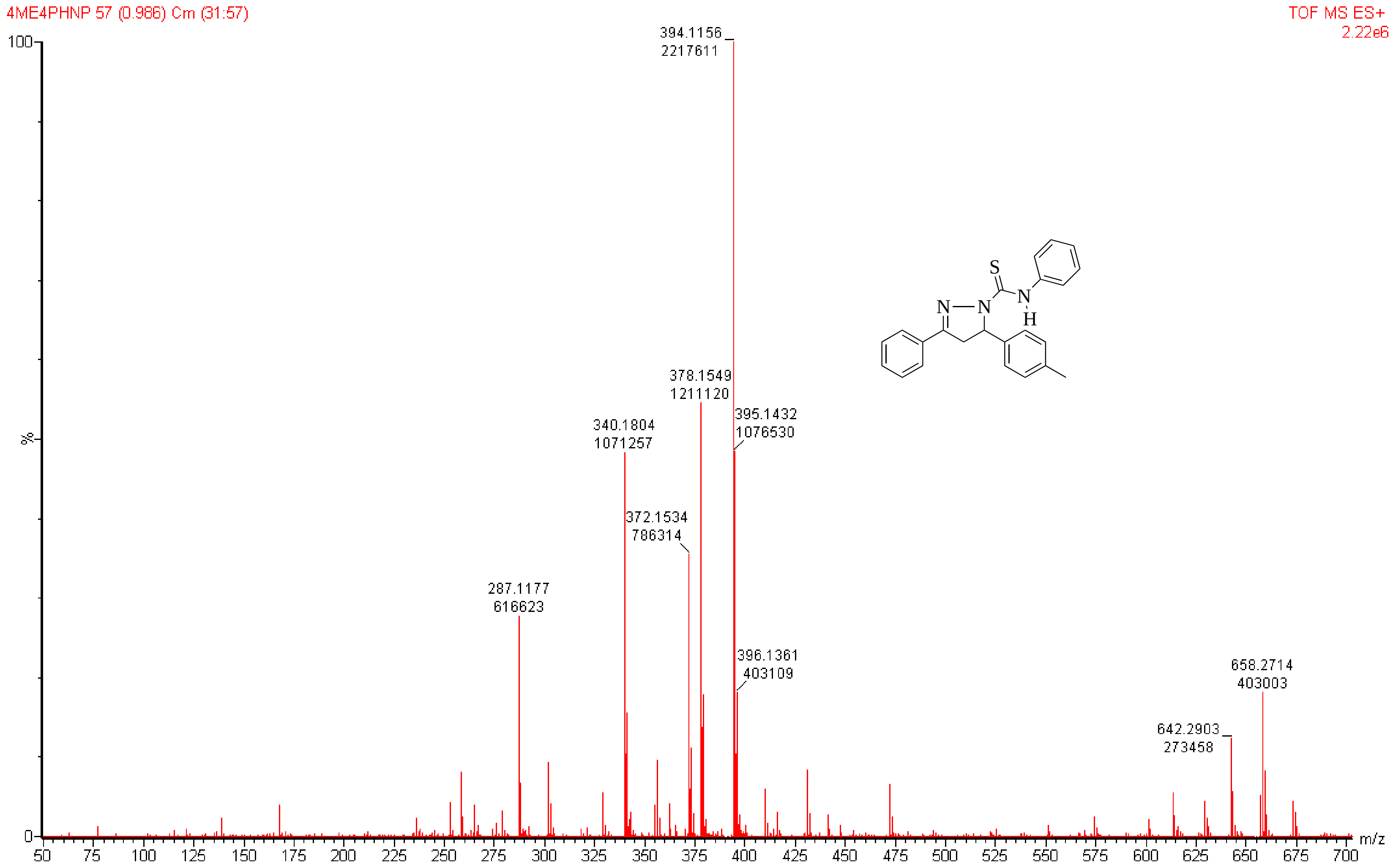

HRMS spectrum of compound **4a**

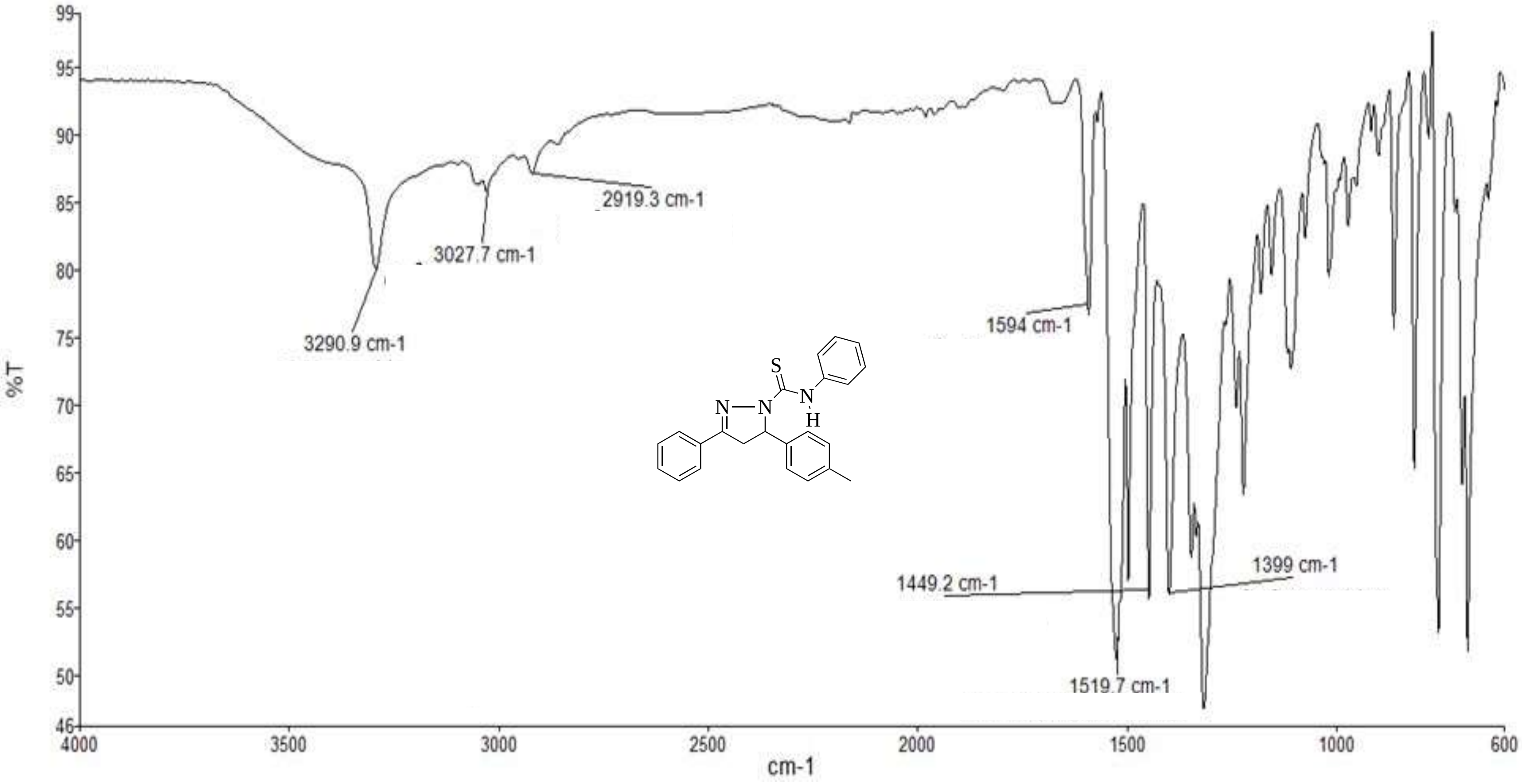

IR spectrum of compound **4a**

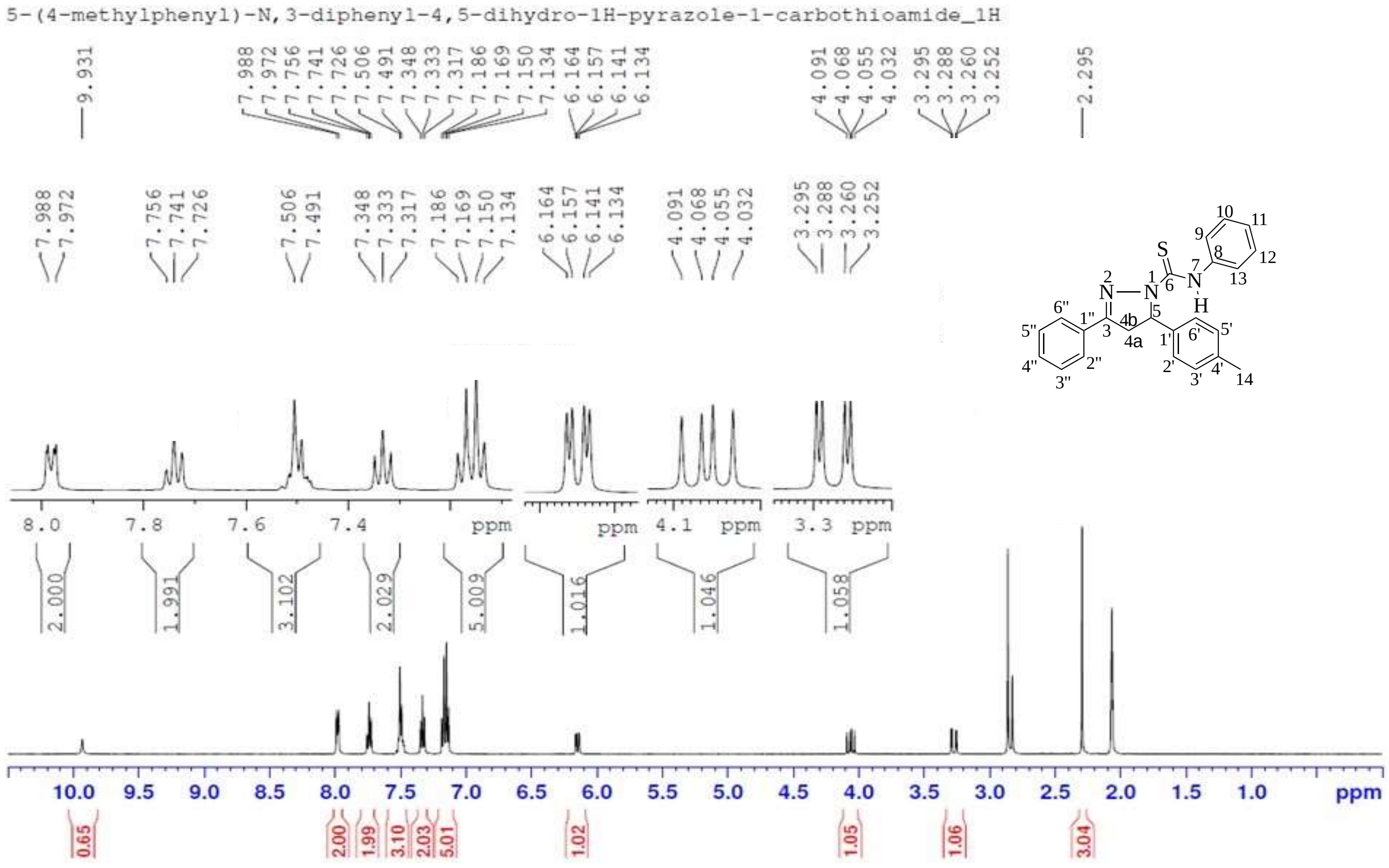

^{1}H NMR spectrum of compound **4a**

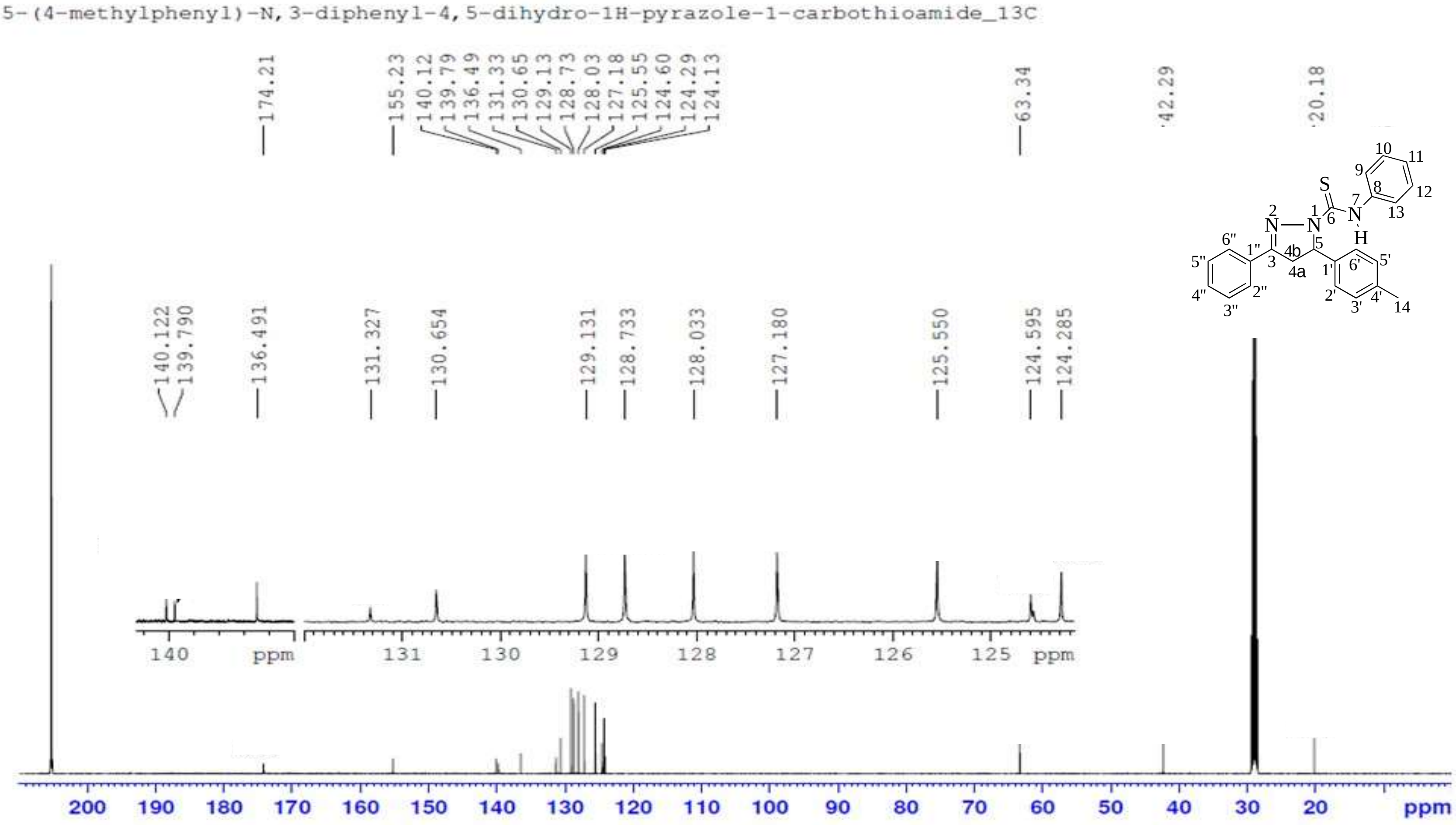

^{13}C NMR spectrum of compound **4a**

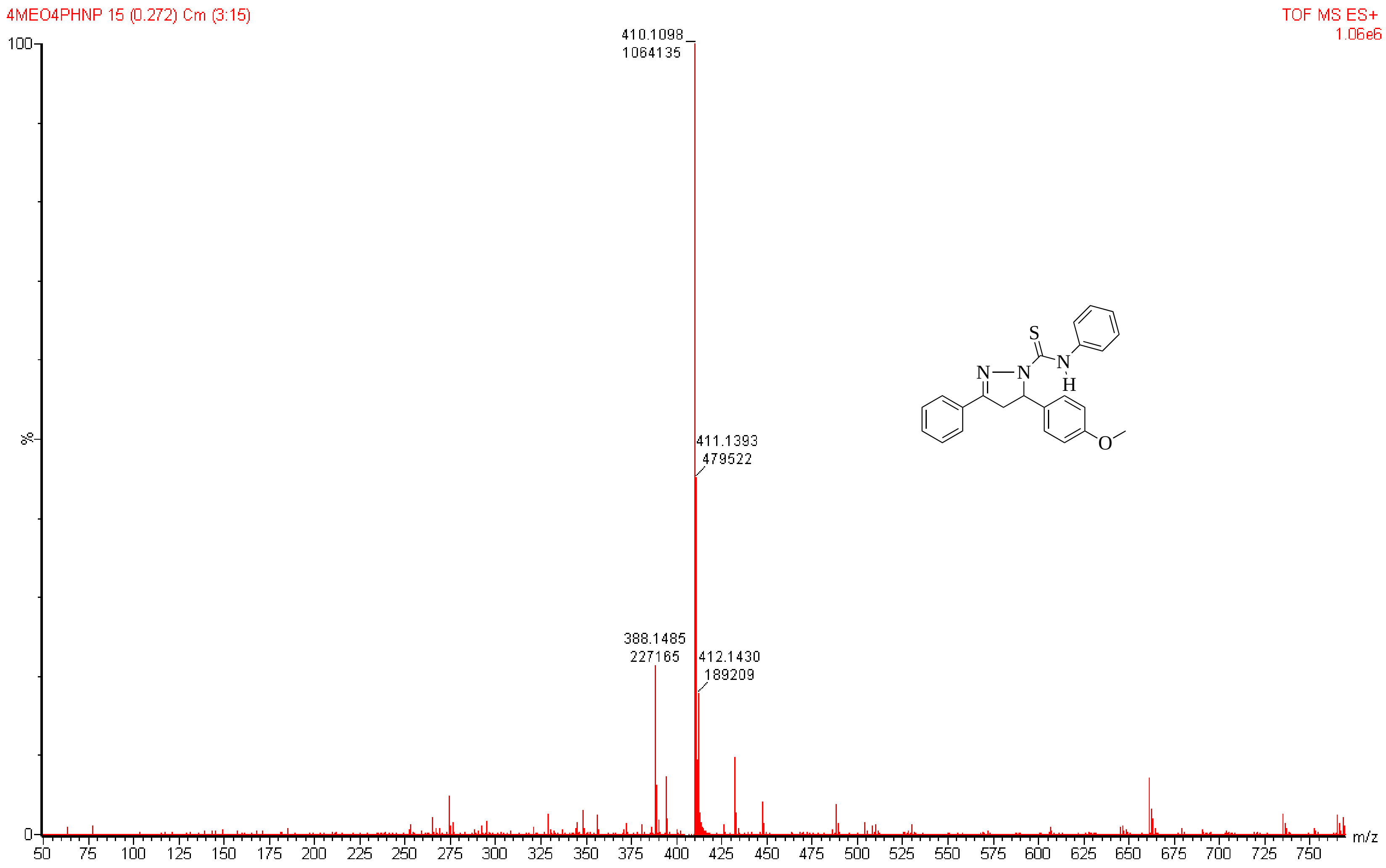

HRMS spectrum of compound **4b**

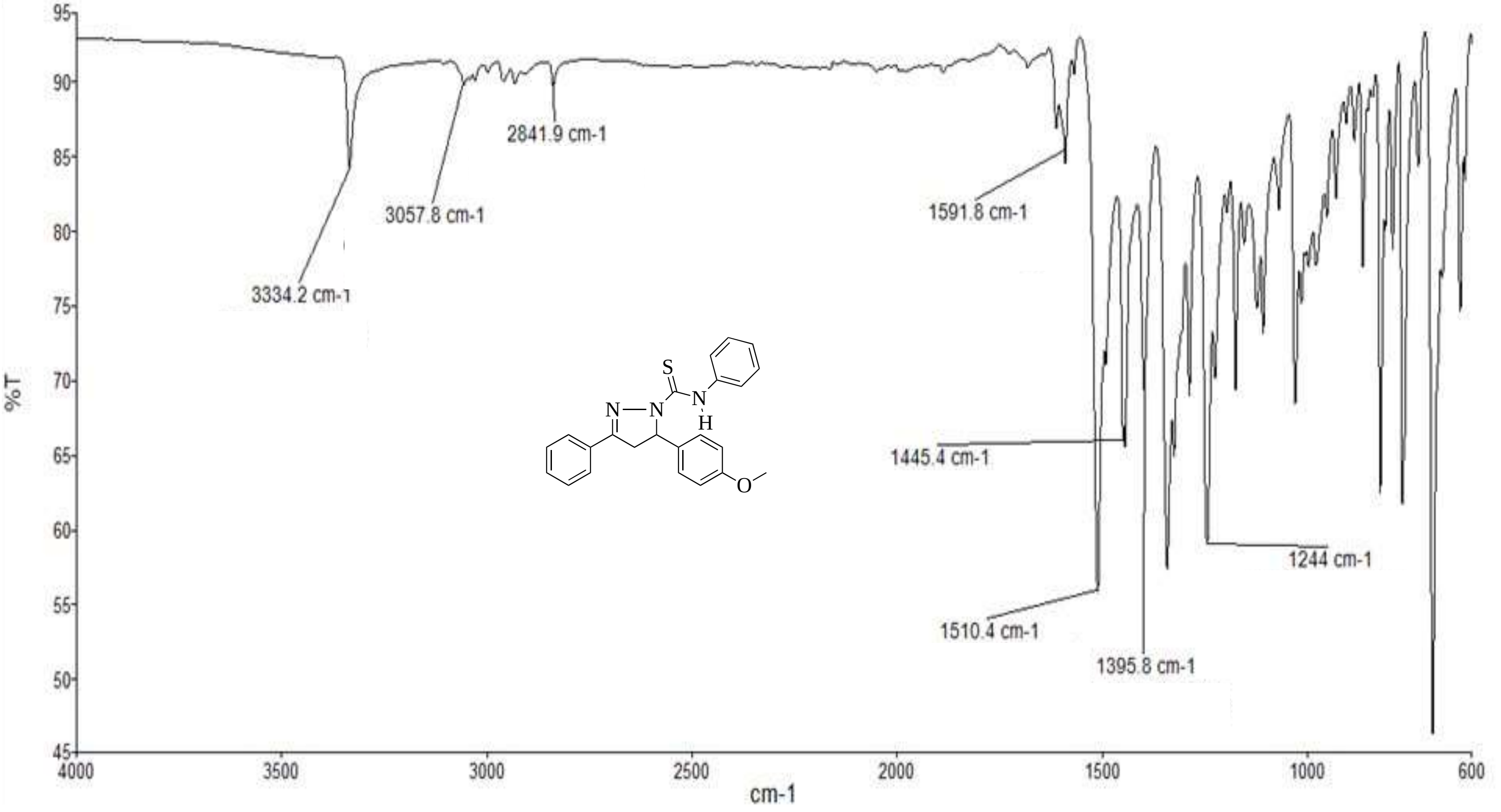

IR spectrum of compound **4b**

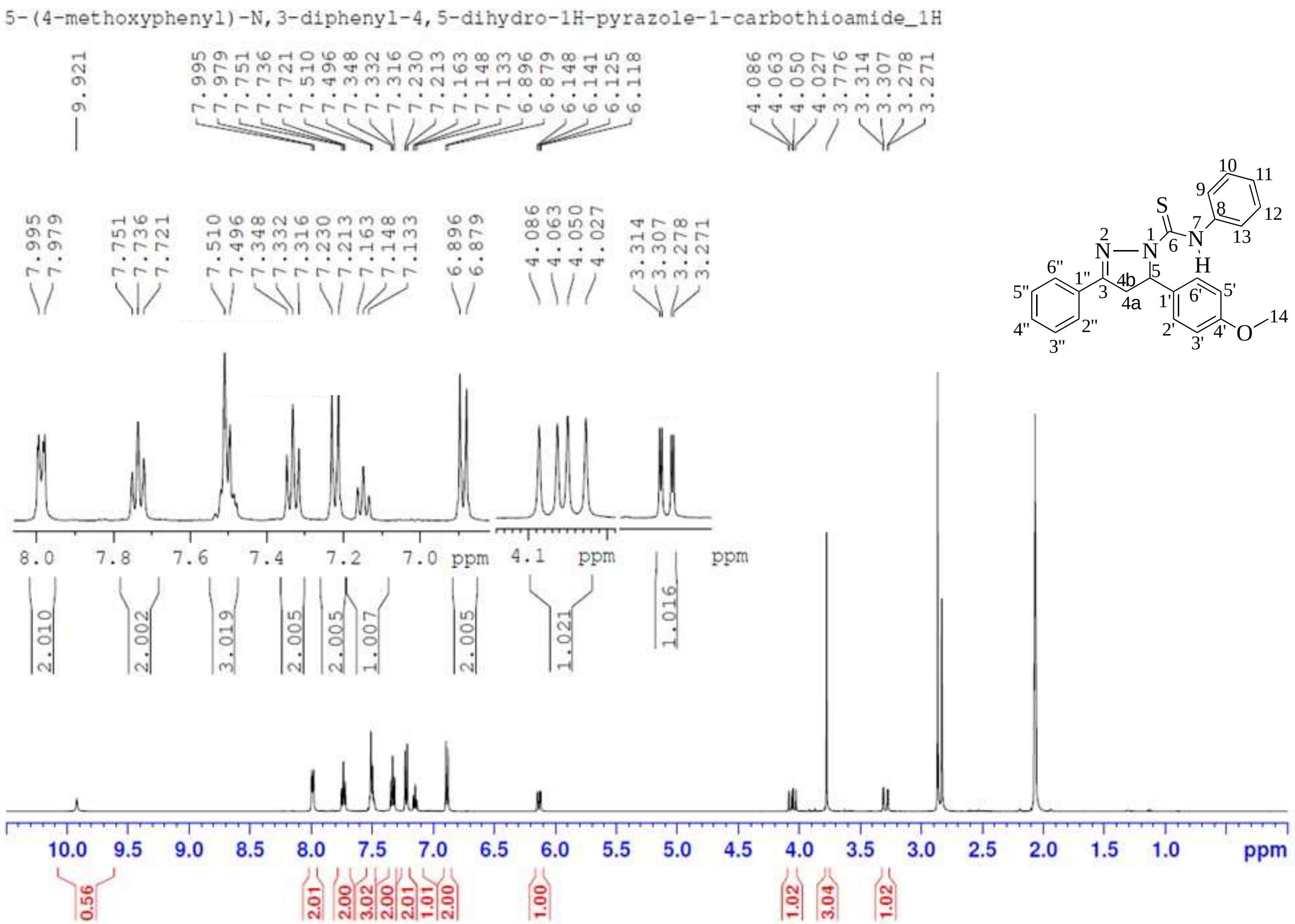

^{1}H NMR spectrum of compound **4b**

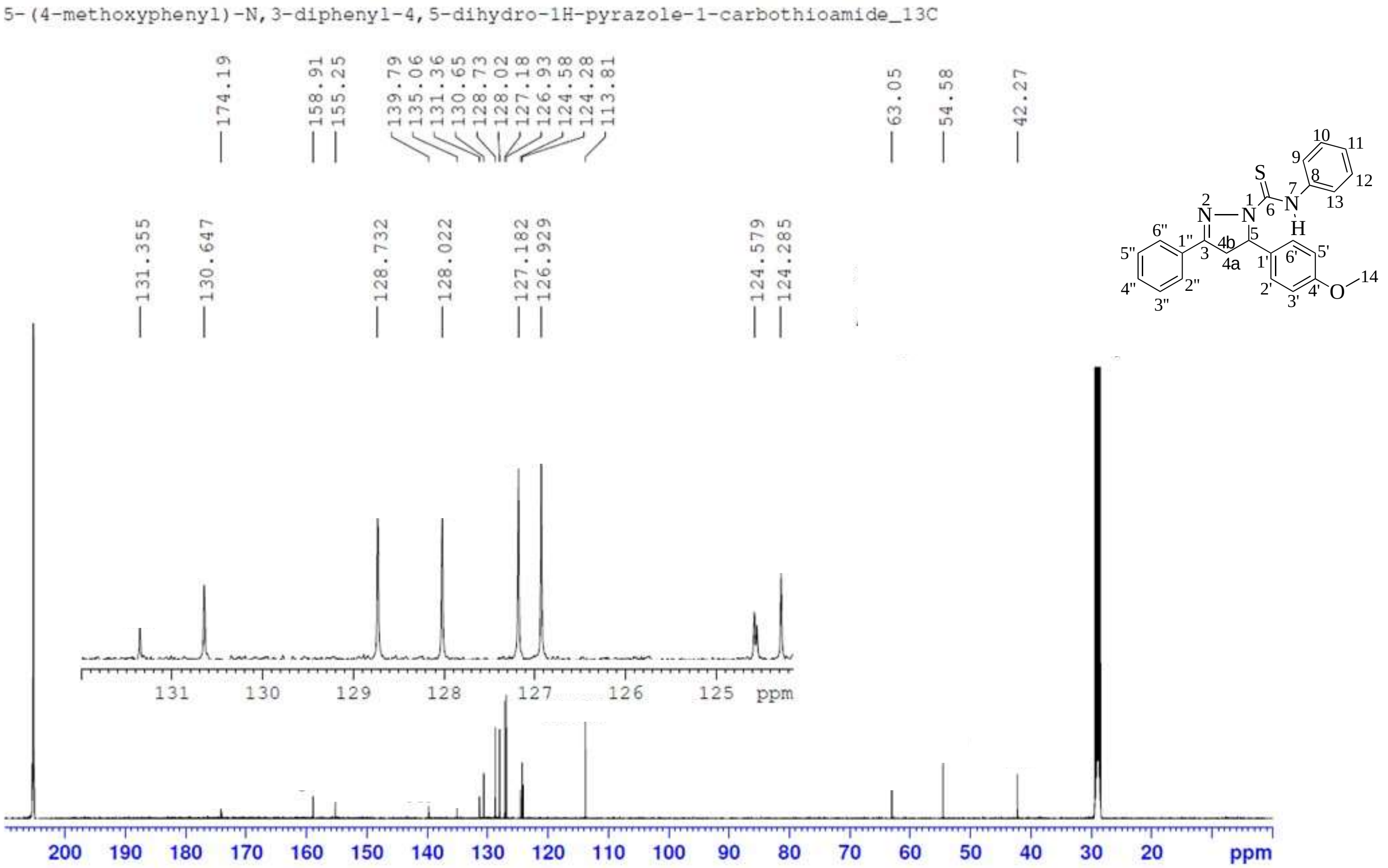

^{13}C NMR spectrum of compound **4b**

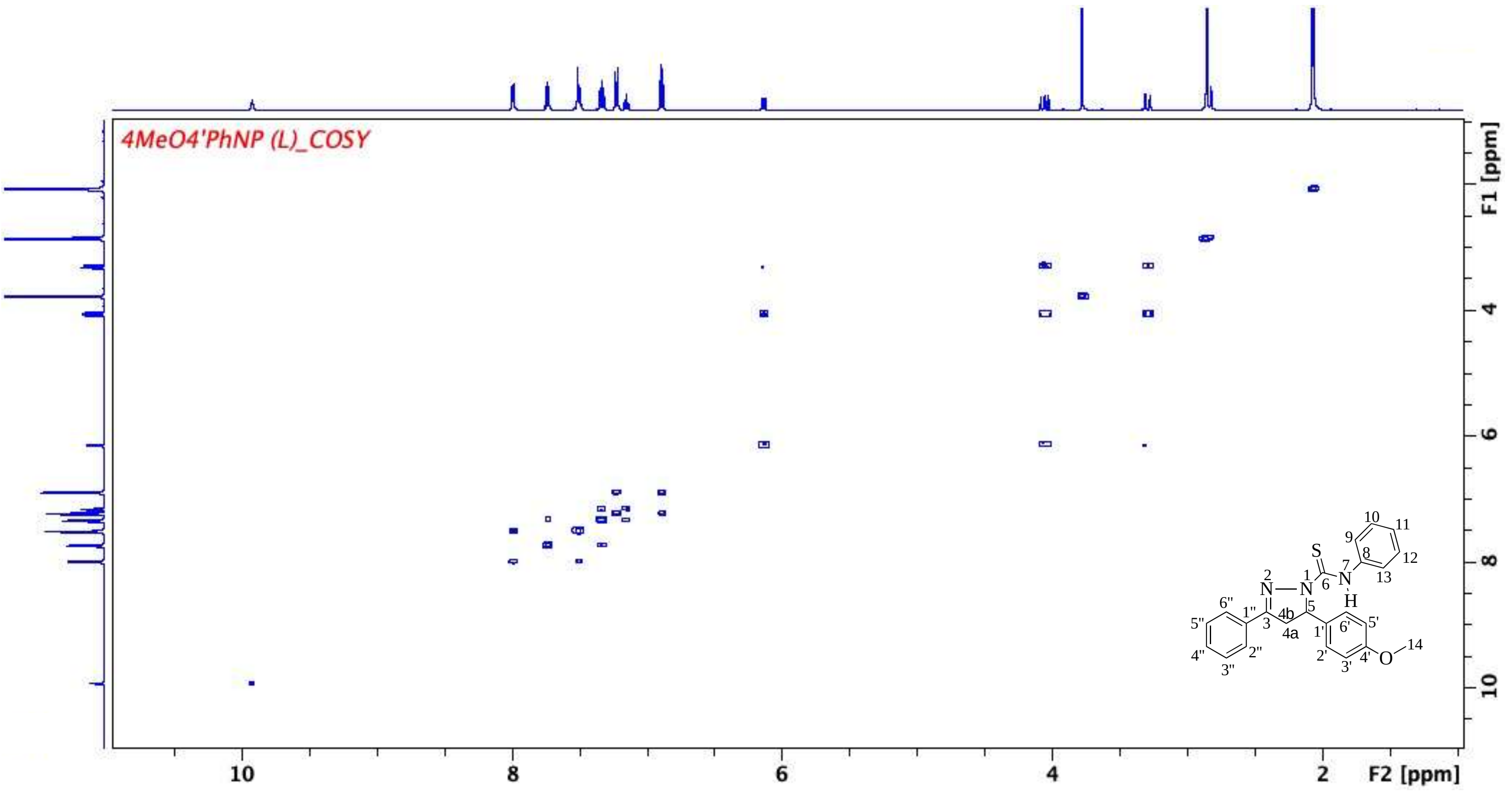

^{1}H-^{1}H COSY NMR spectrum of compound 4b

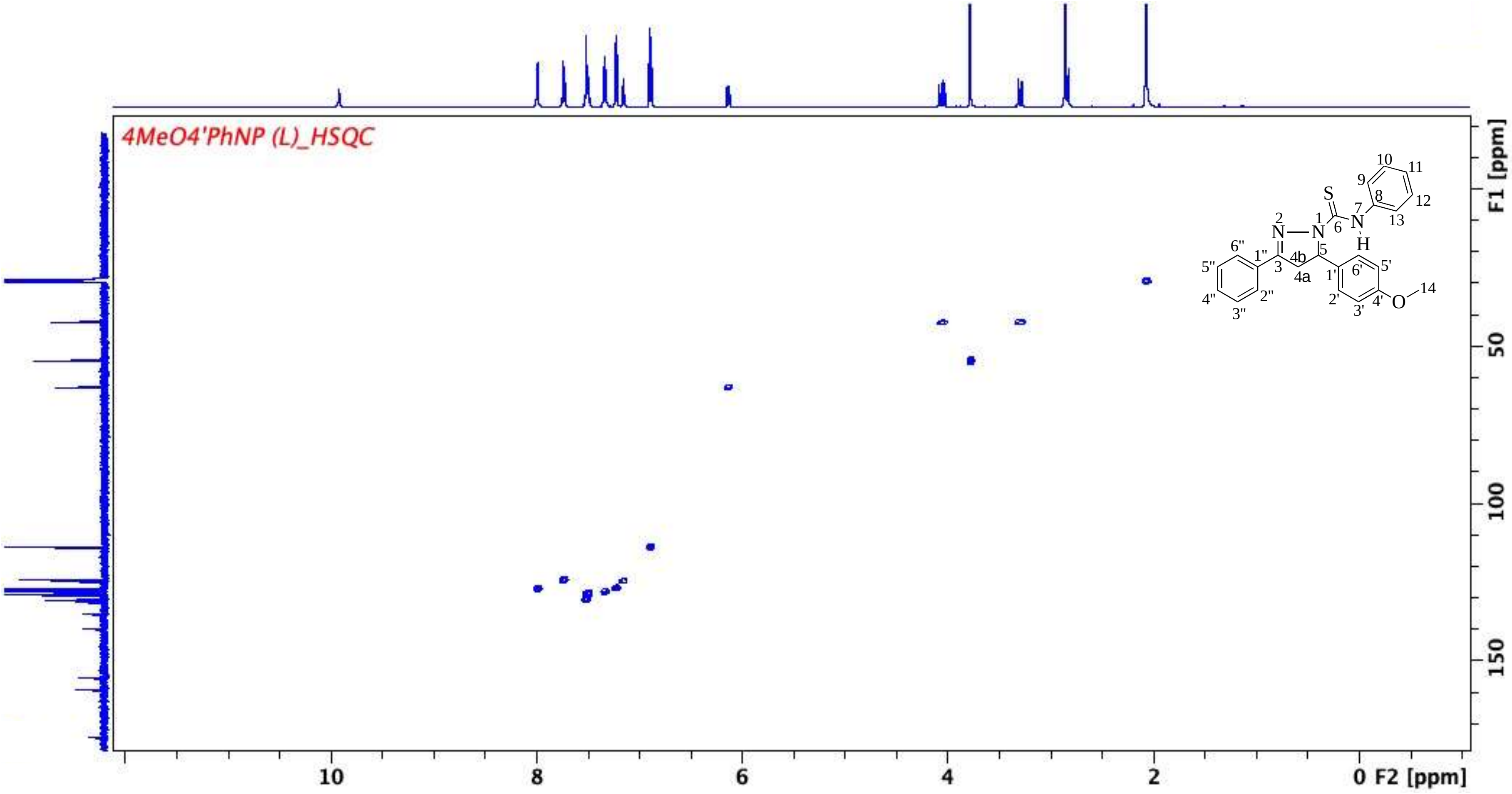

^{1}H-^{13}C HSQC NMR spectrum of compound 4b

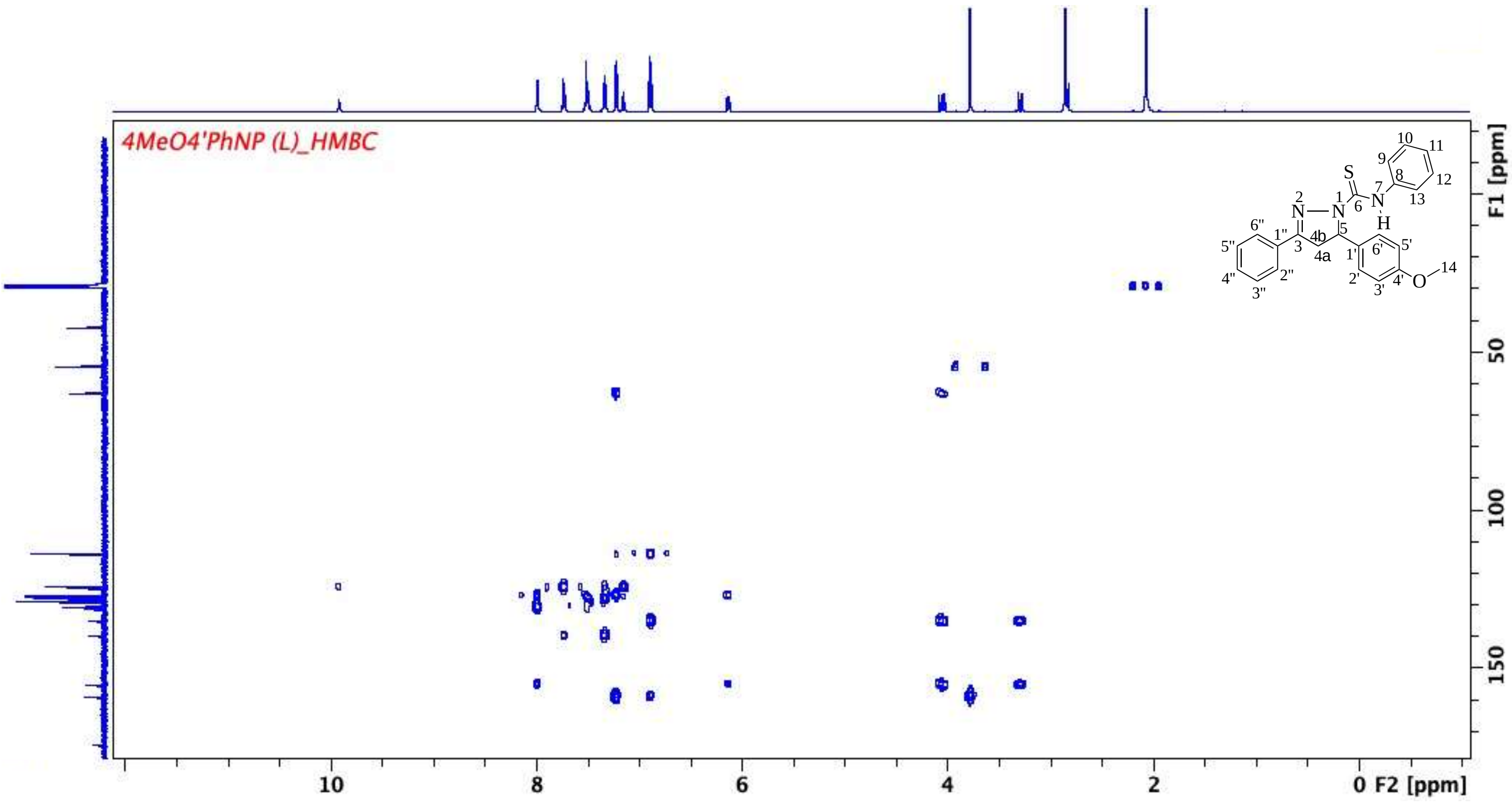

^{1}H-^{13}C HMBC NMR spectrum of compound 4b

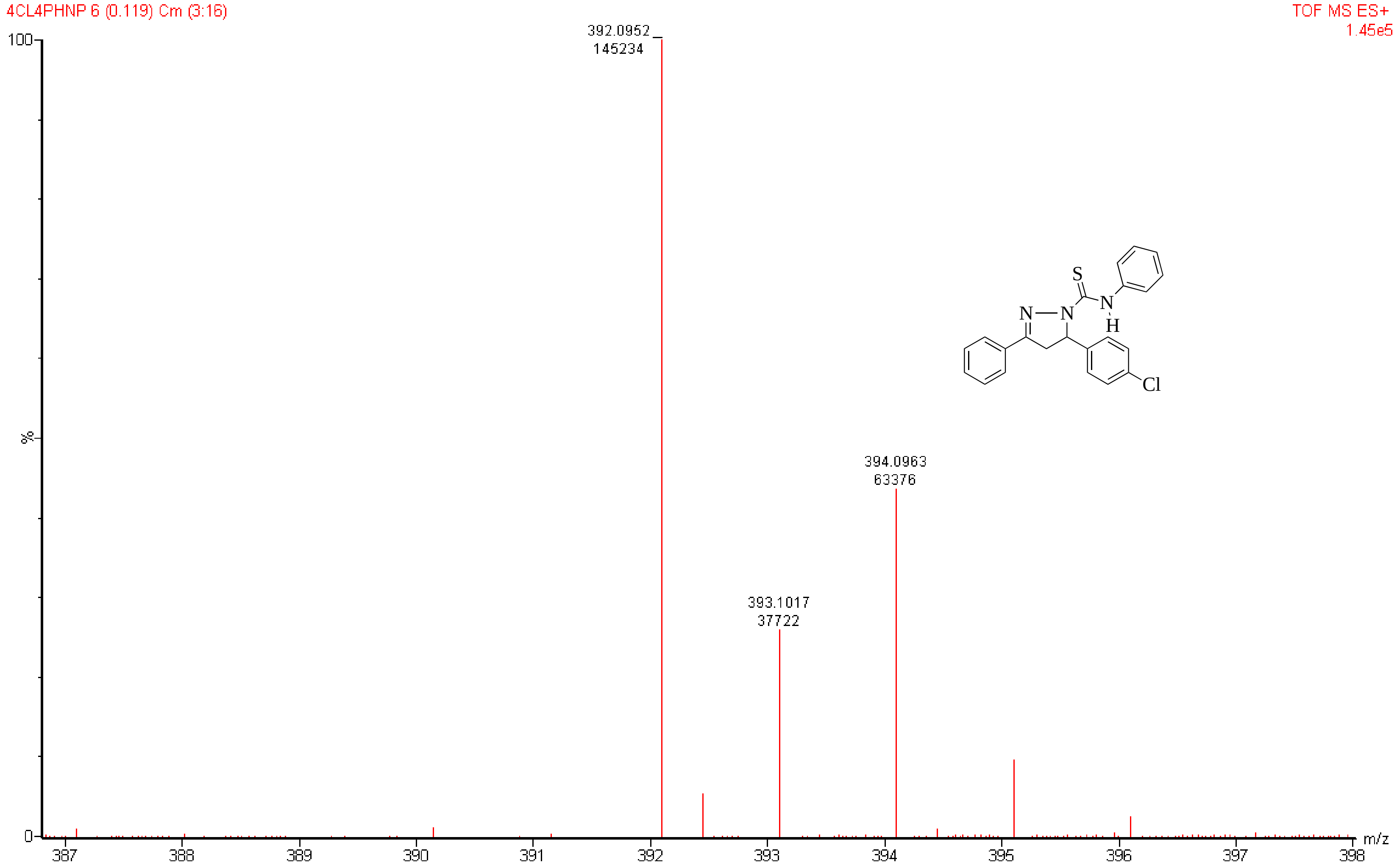

HRMS spectrum of compound **4c**

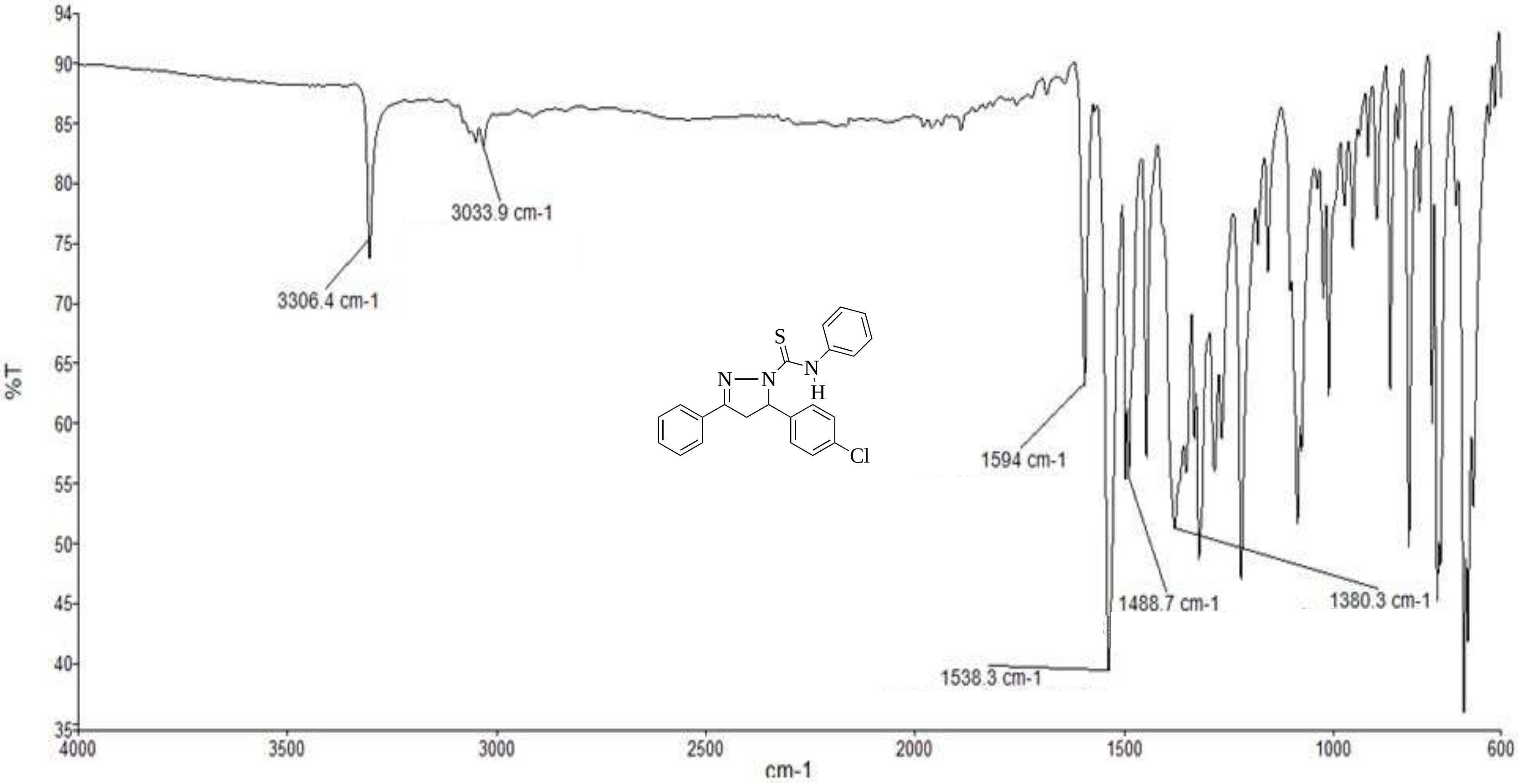

IR spectrum of compound **4c**

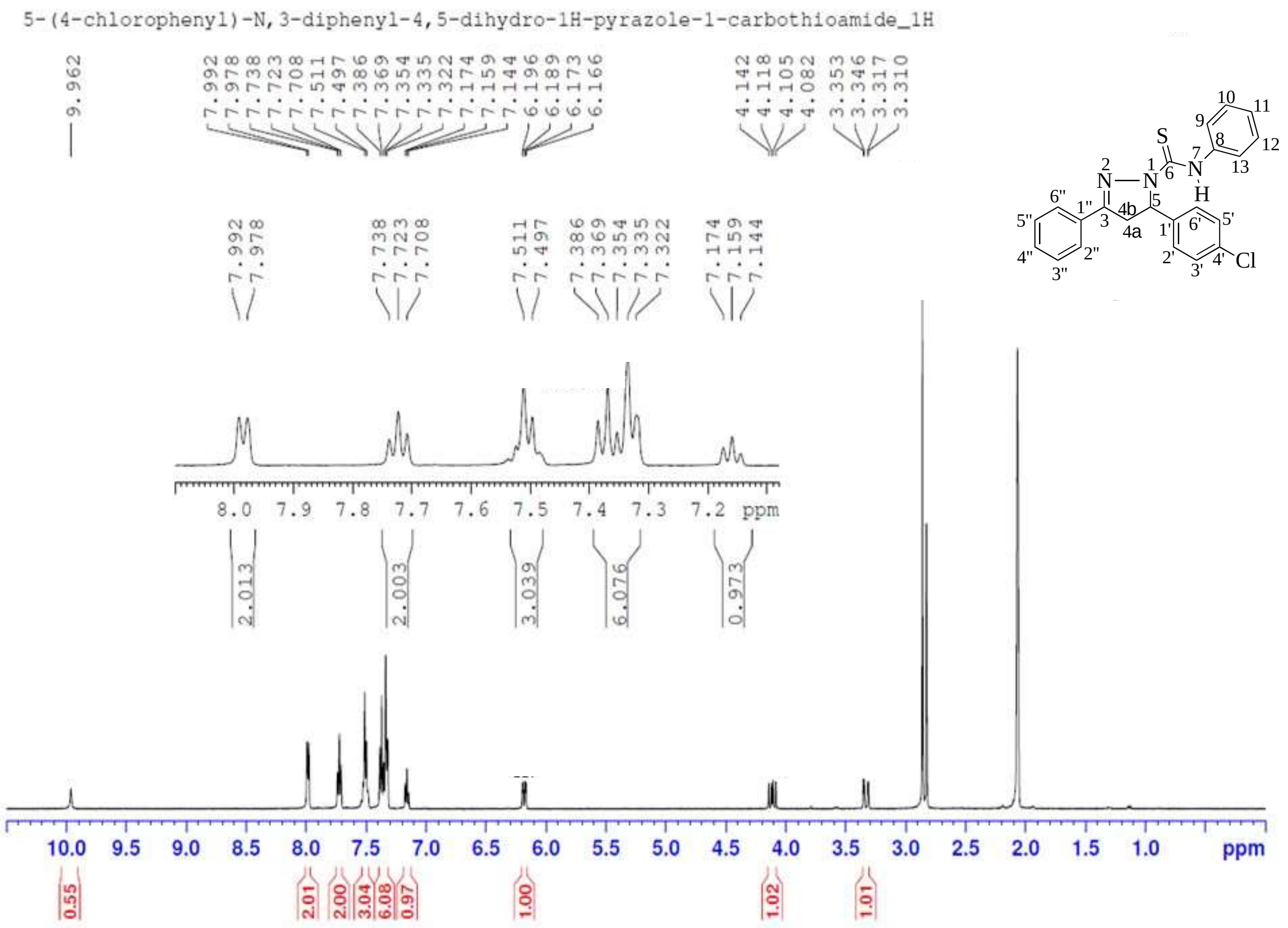

^{1}H NMR spectrum of compound **4c**

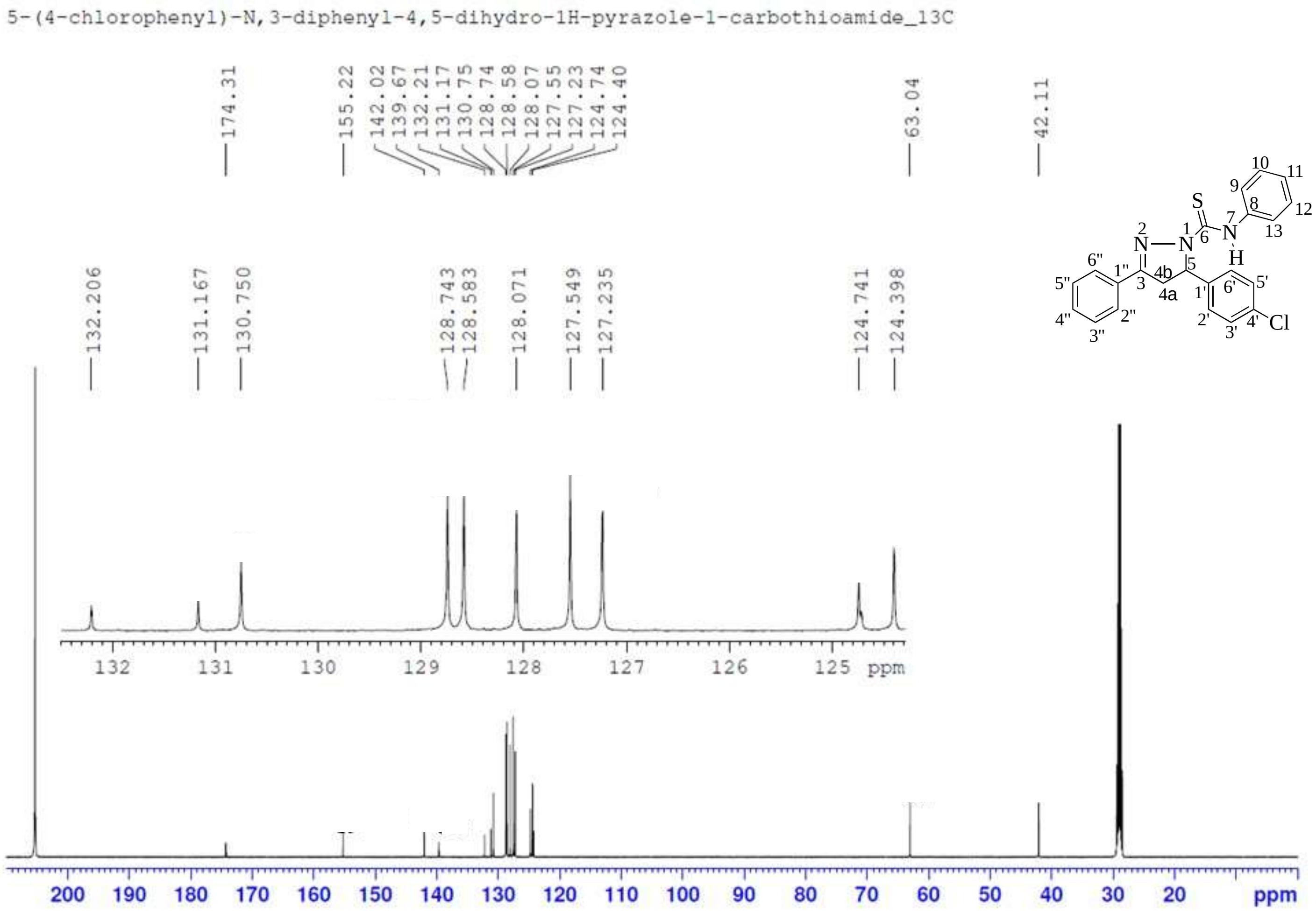

^{13}C NMR spectrum of compound **4c**

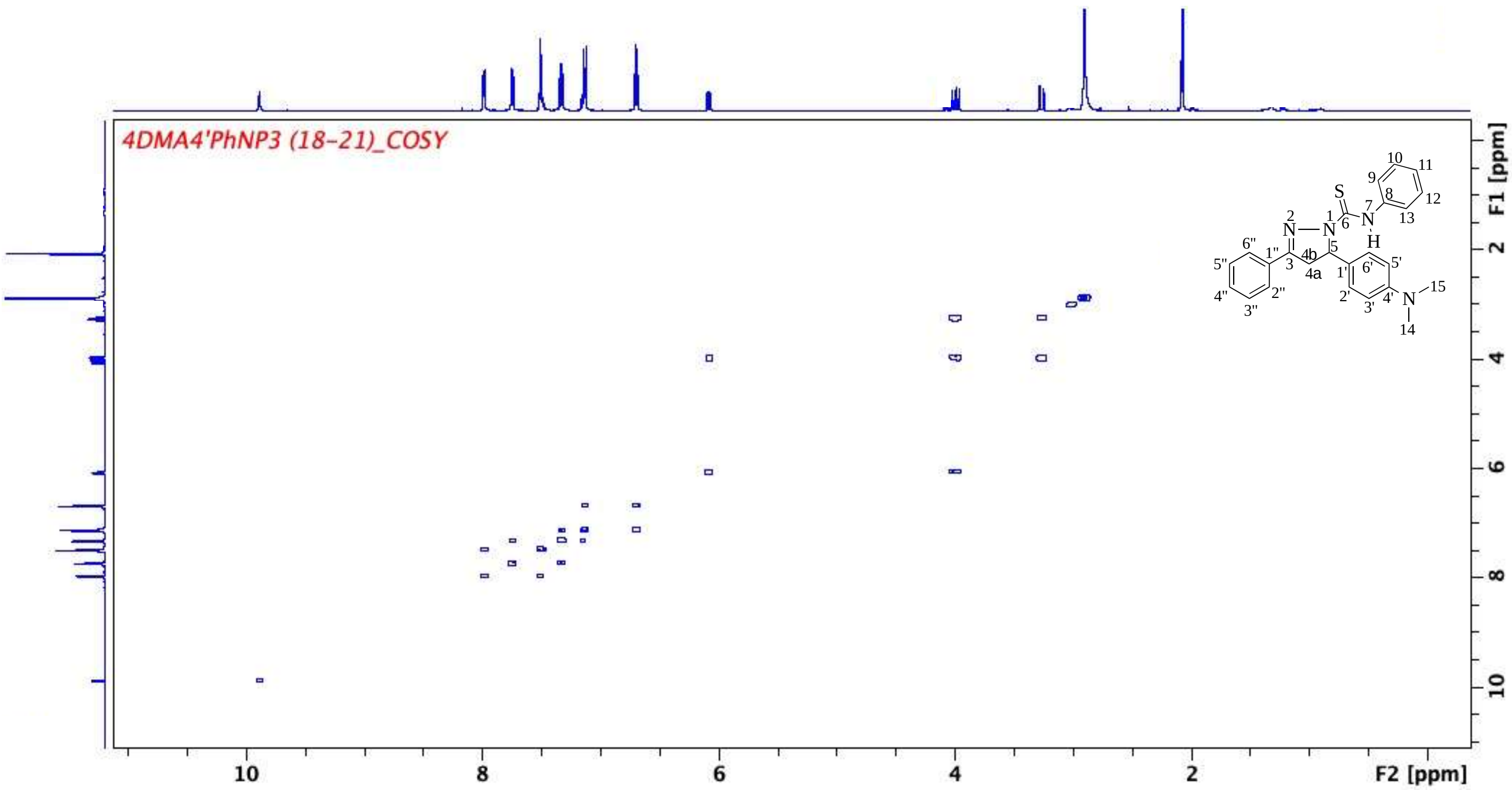

^1H-^1H COSY NMR spectrum of compound 4d

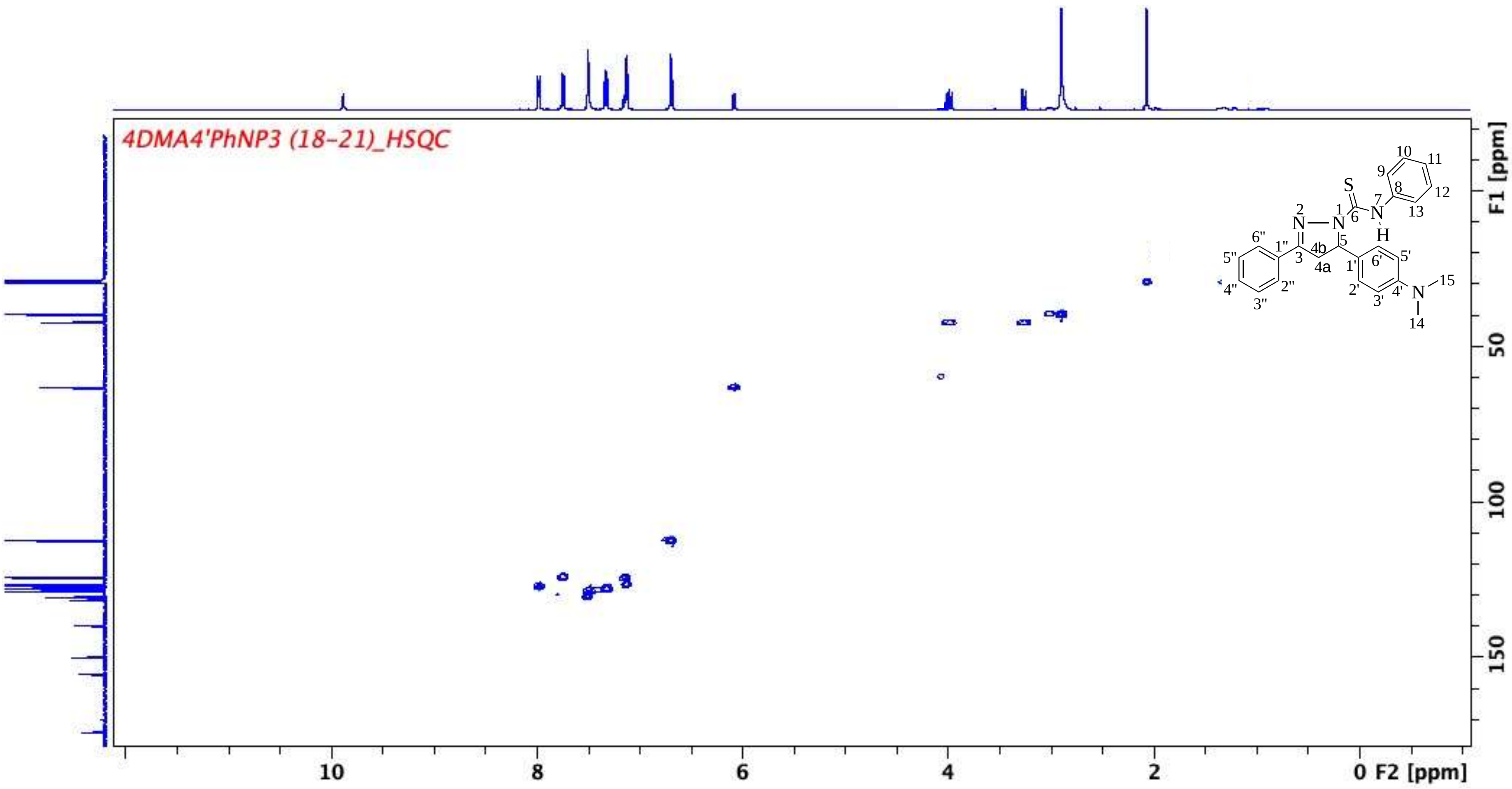

^{1}H-^{13}C HSQC NMR spectrum of compound 4d

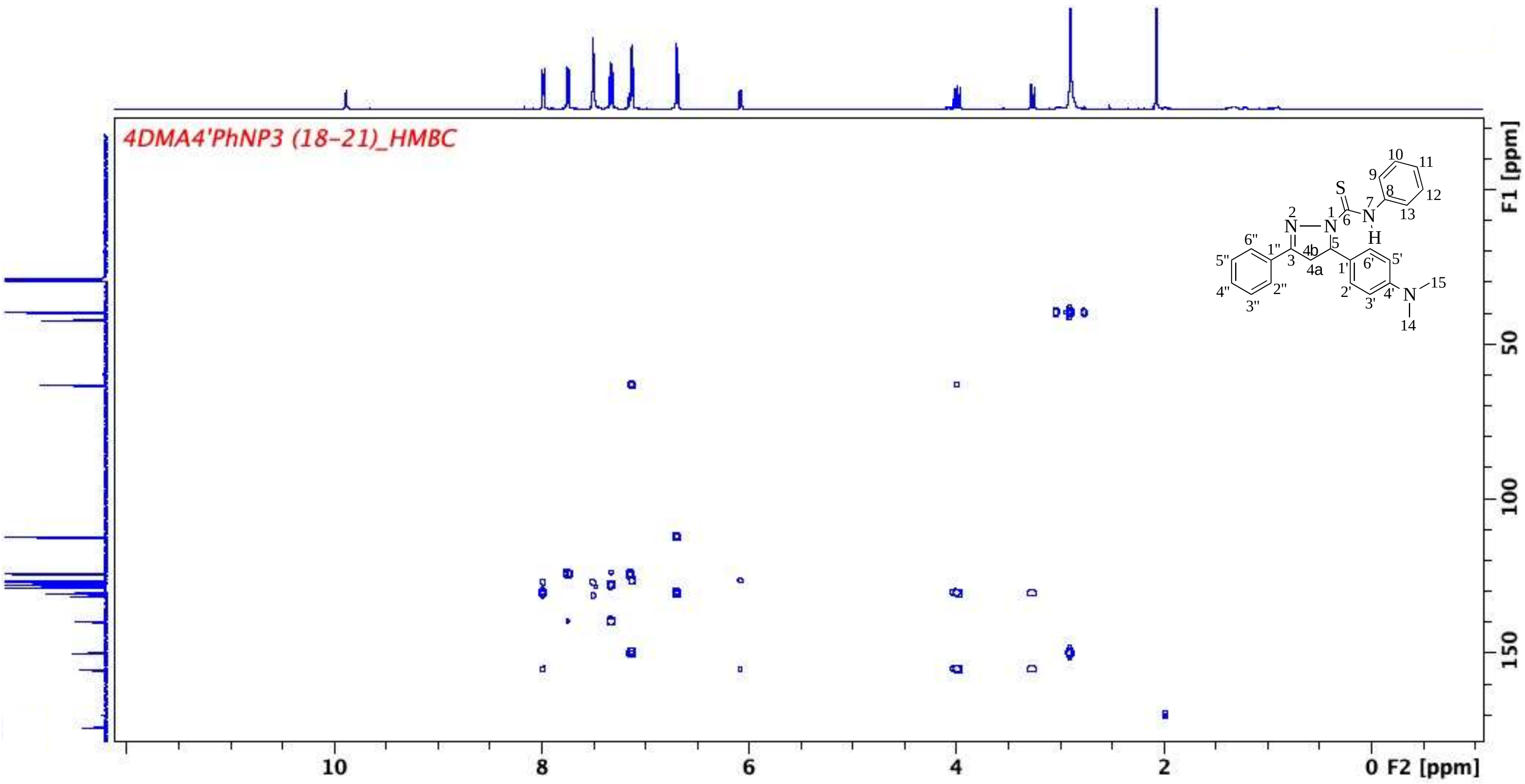

^{1}H-^{13}C HMBC NMR spectrum of compound 4d

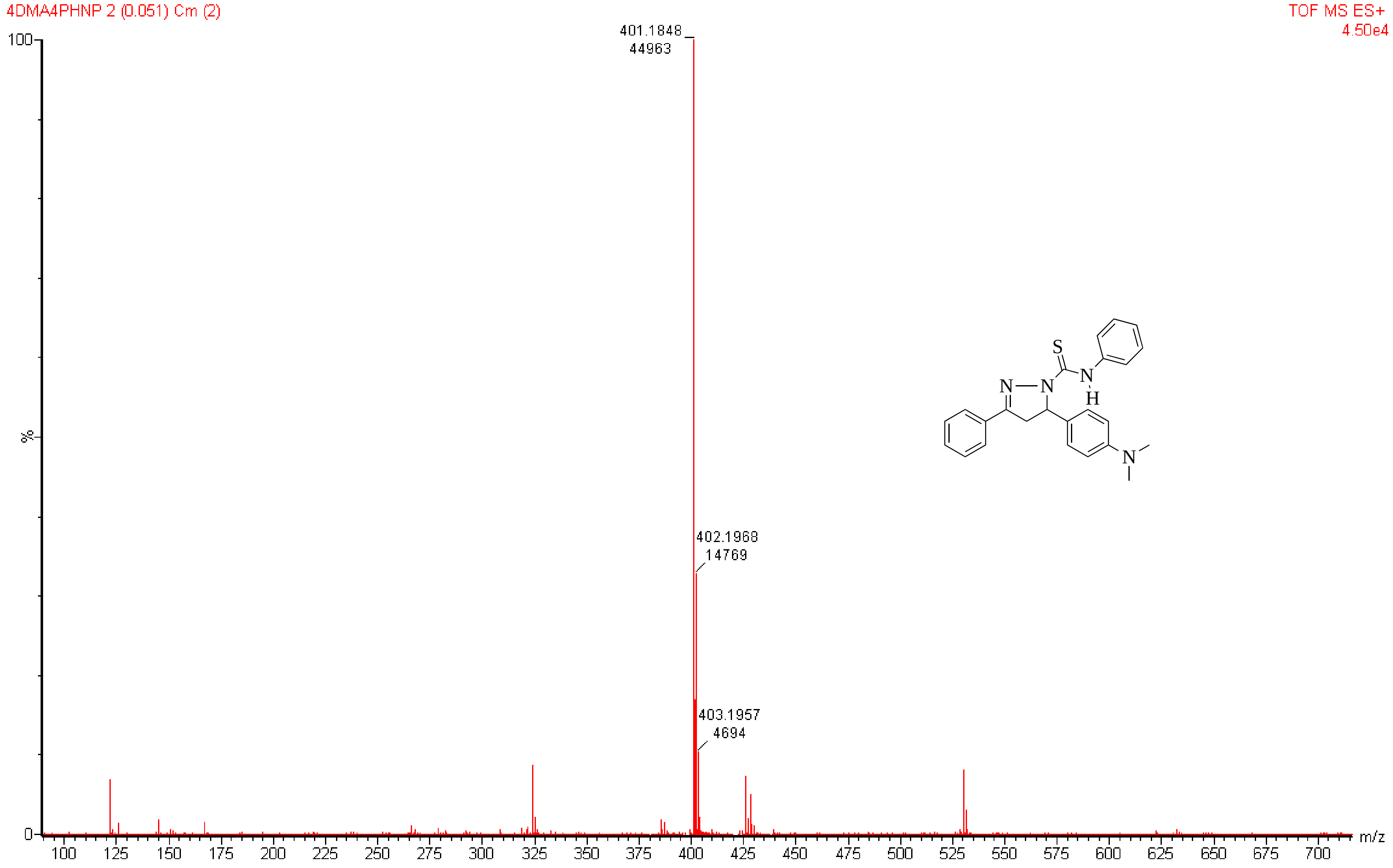

HRMS spectrum of compound **4d**

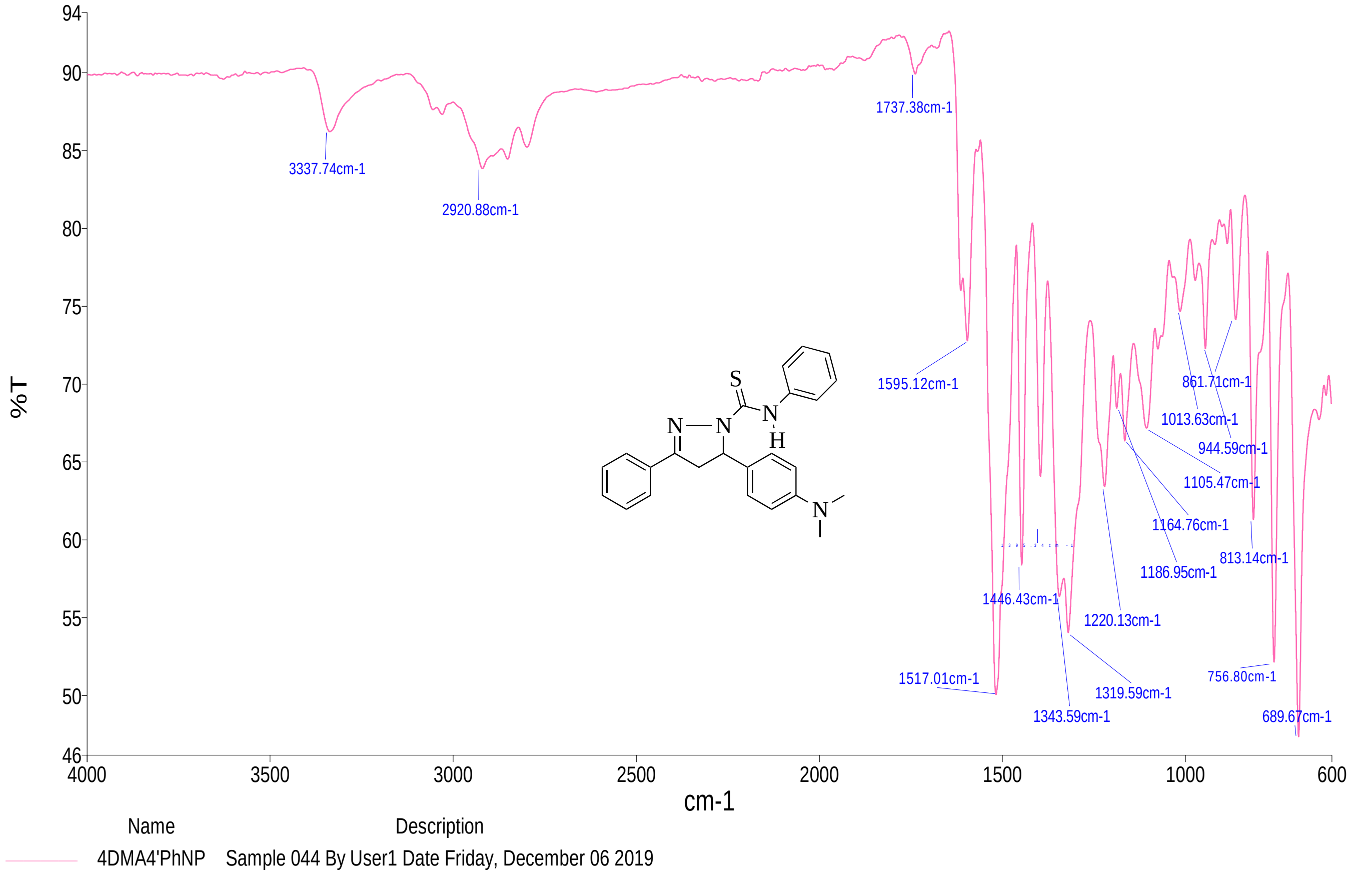

IR spectrum of compound **4d**

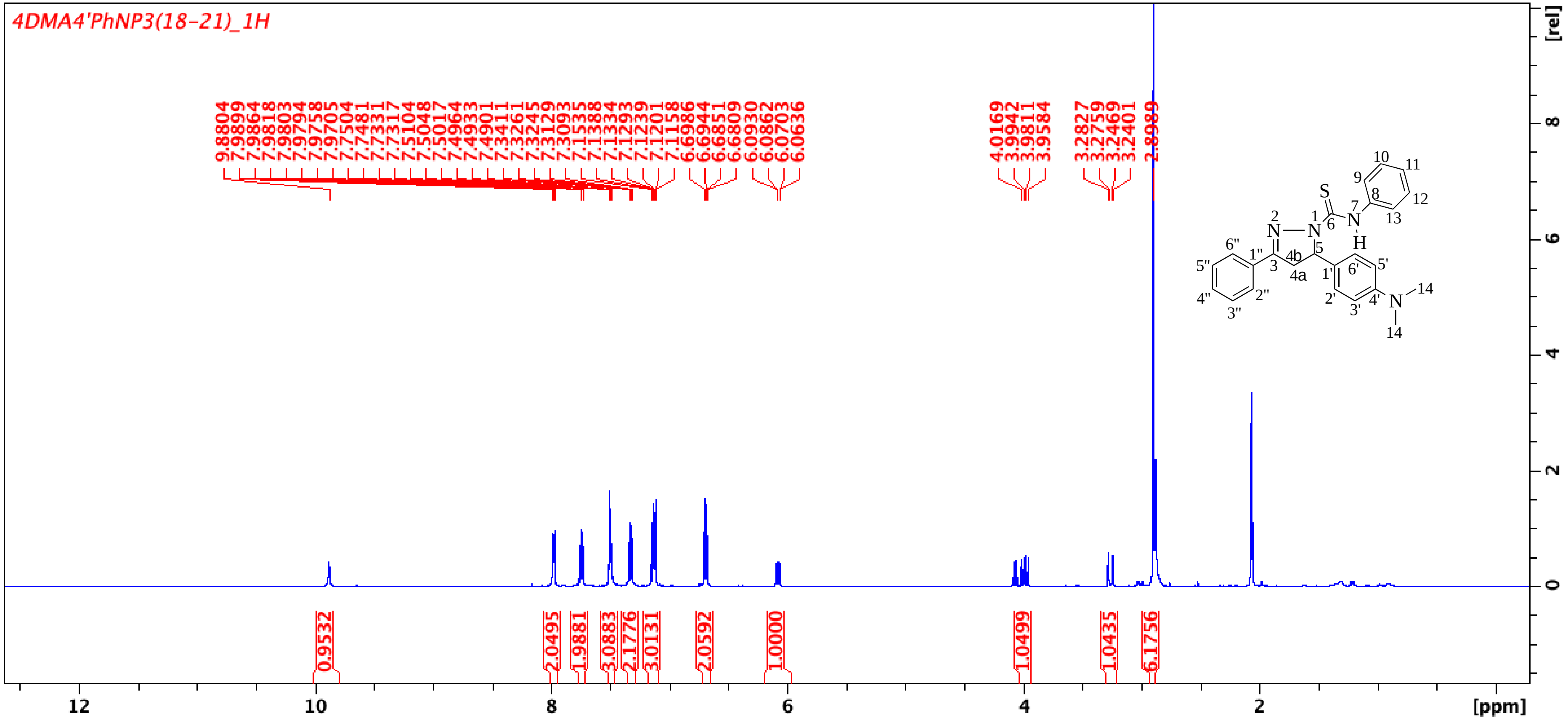

^{1}H NMR spectrum of compound **4d**

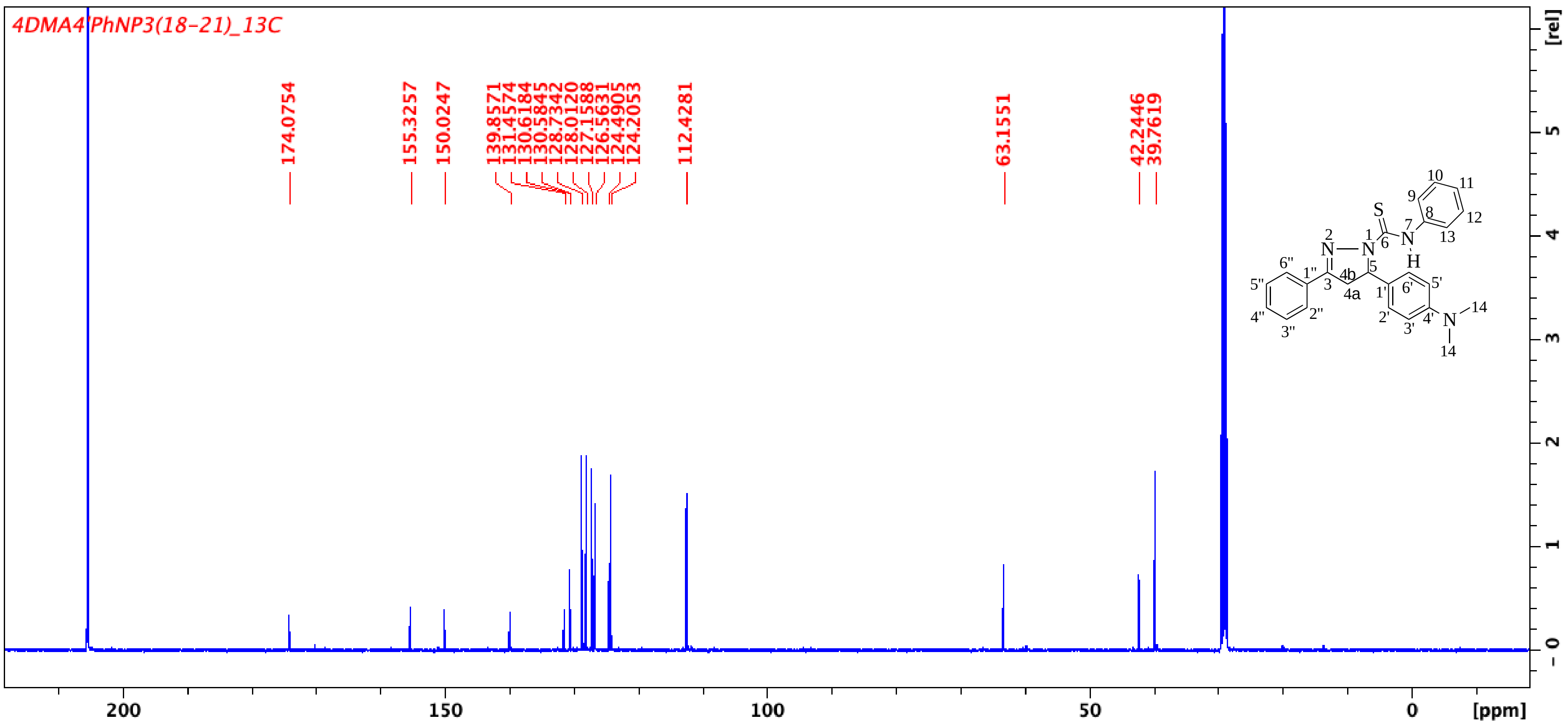

^{13}C NMR spectrum of compound **4d**

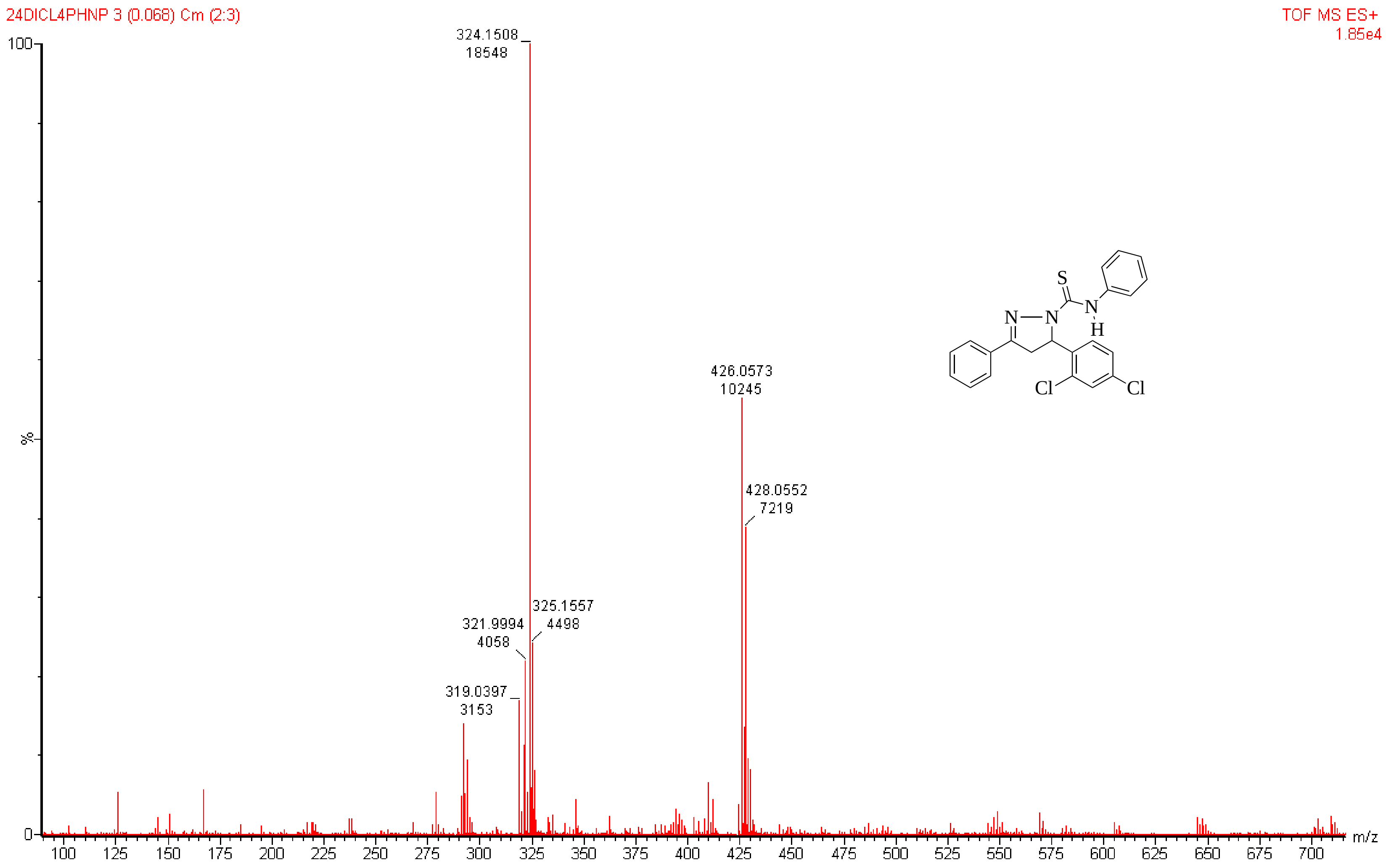

HRMS spectrum of compound **4e**

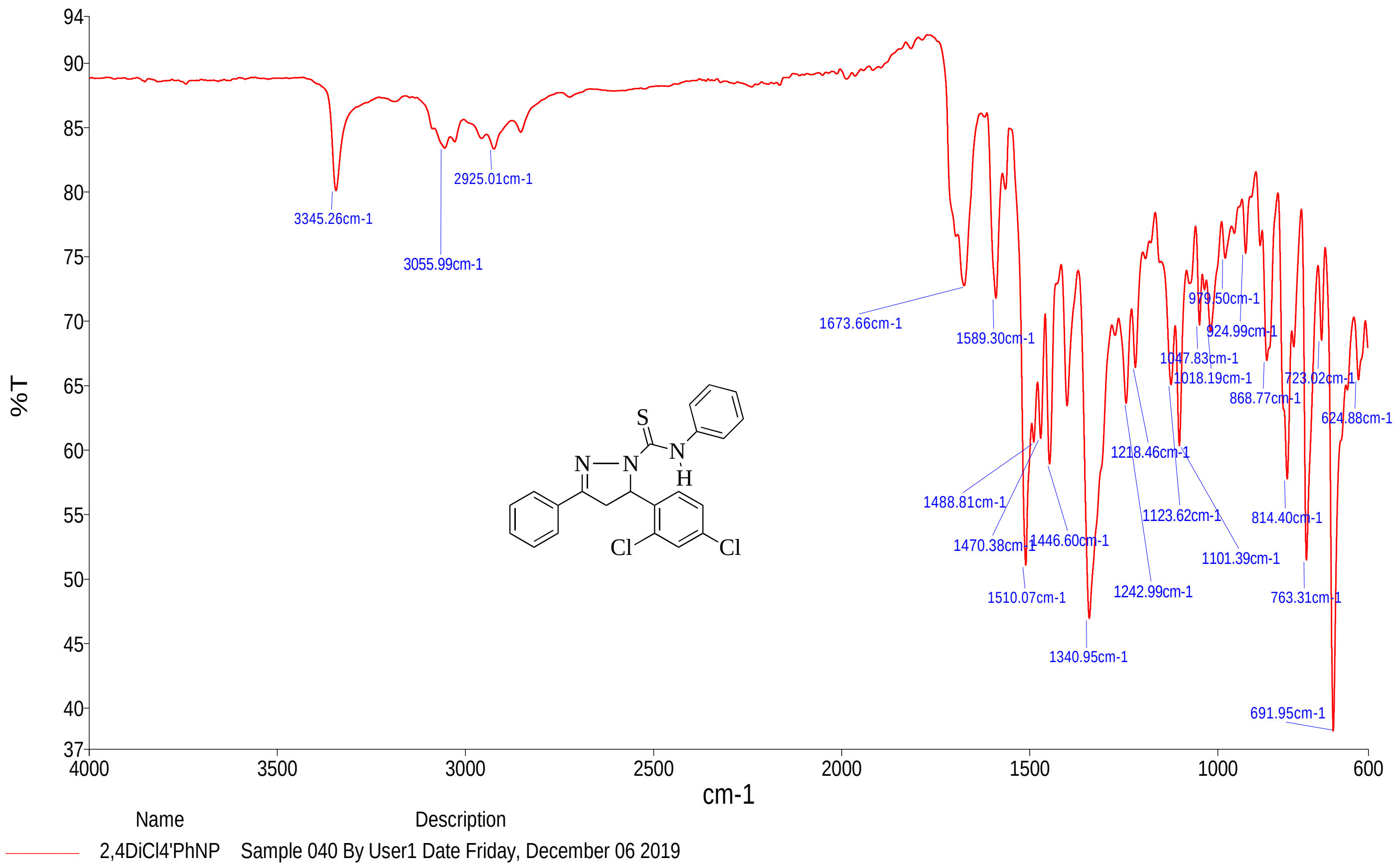

IR spectrum of compound **4e**

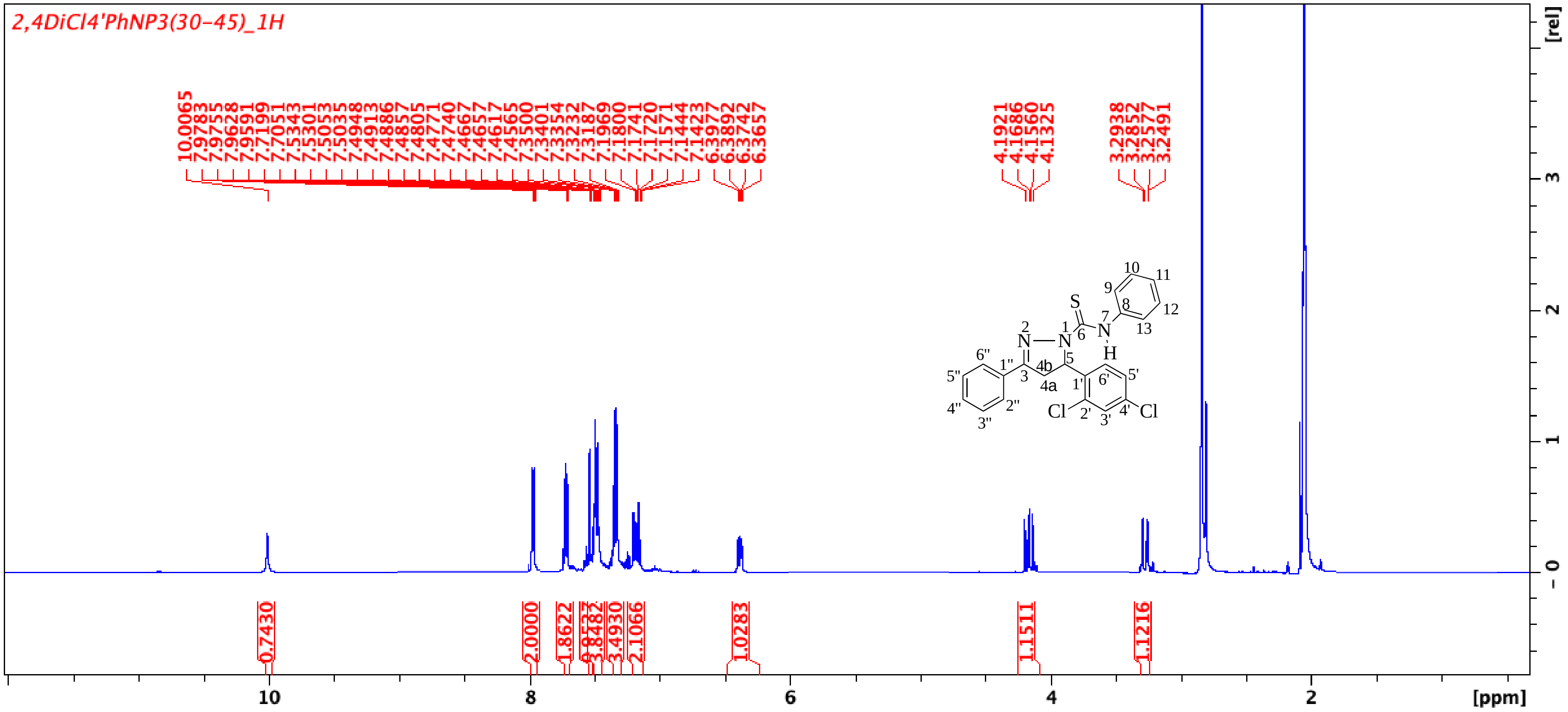

^{1}H NMR spectrum of compound **4e**

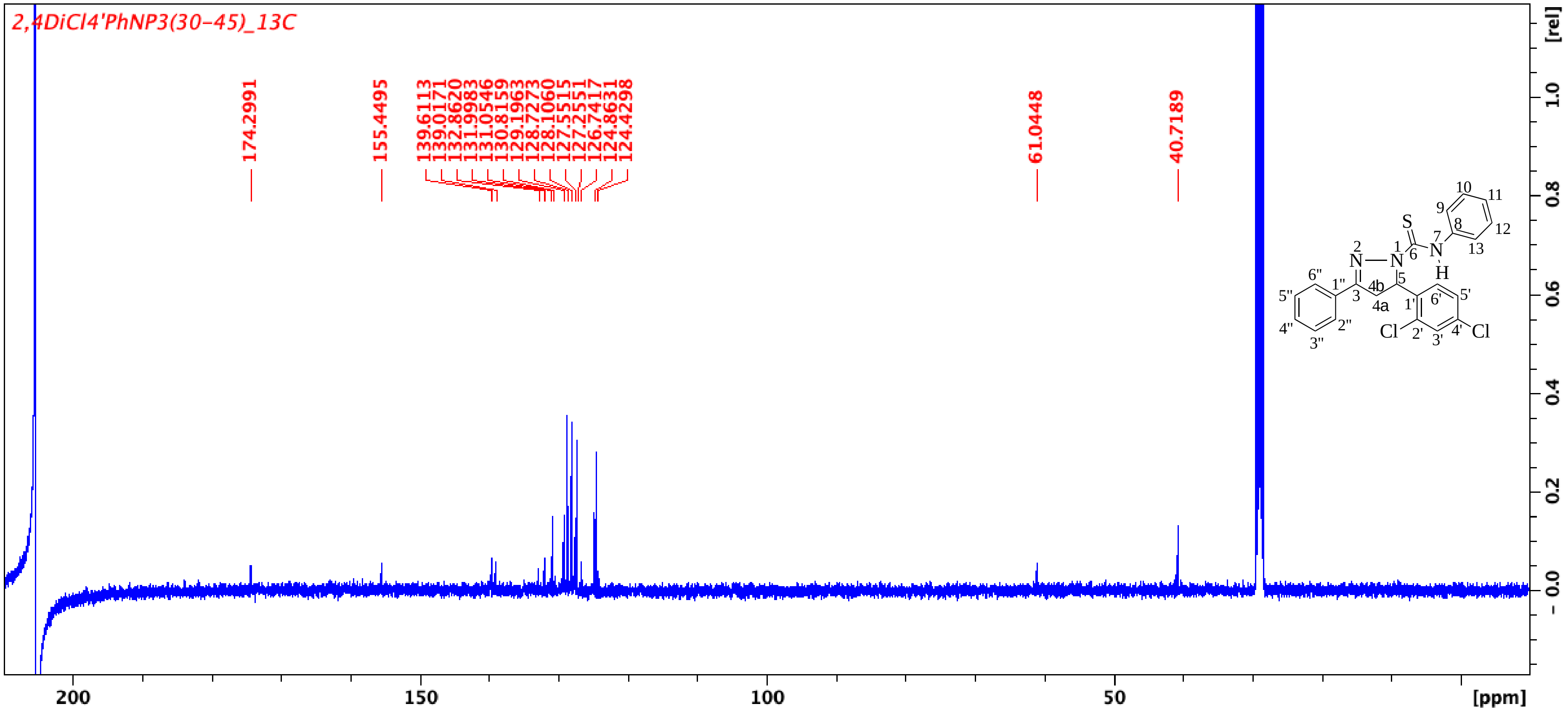

^{13}C NMR spectrum of compound **4e**

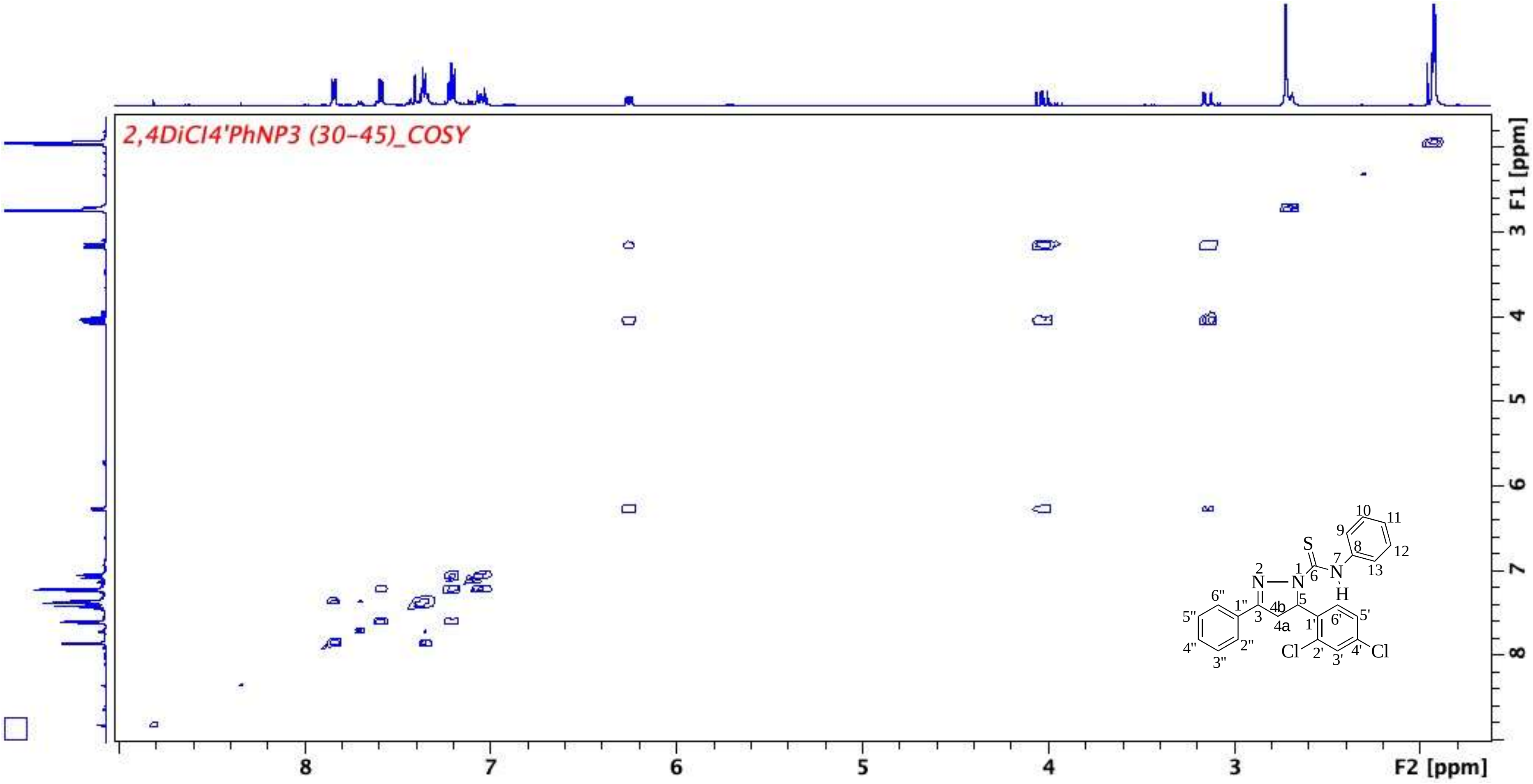

^{1}H-^{1}H COSY NMR spectrum of compound 4e

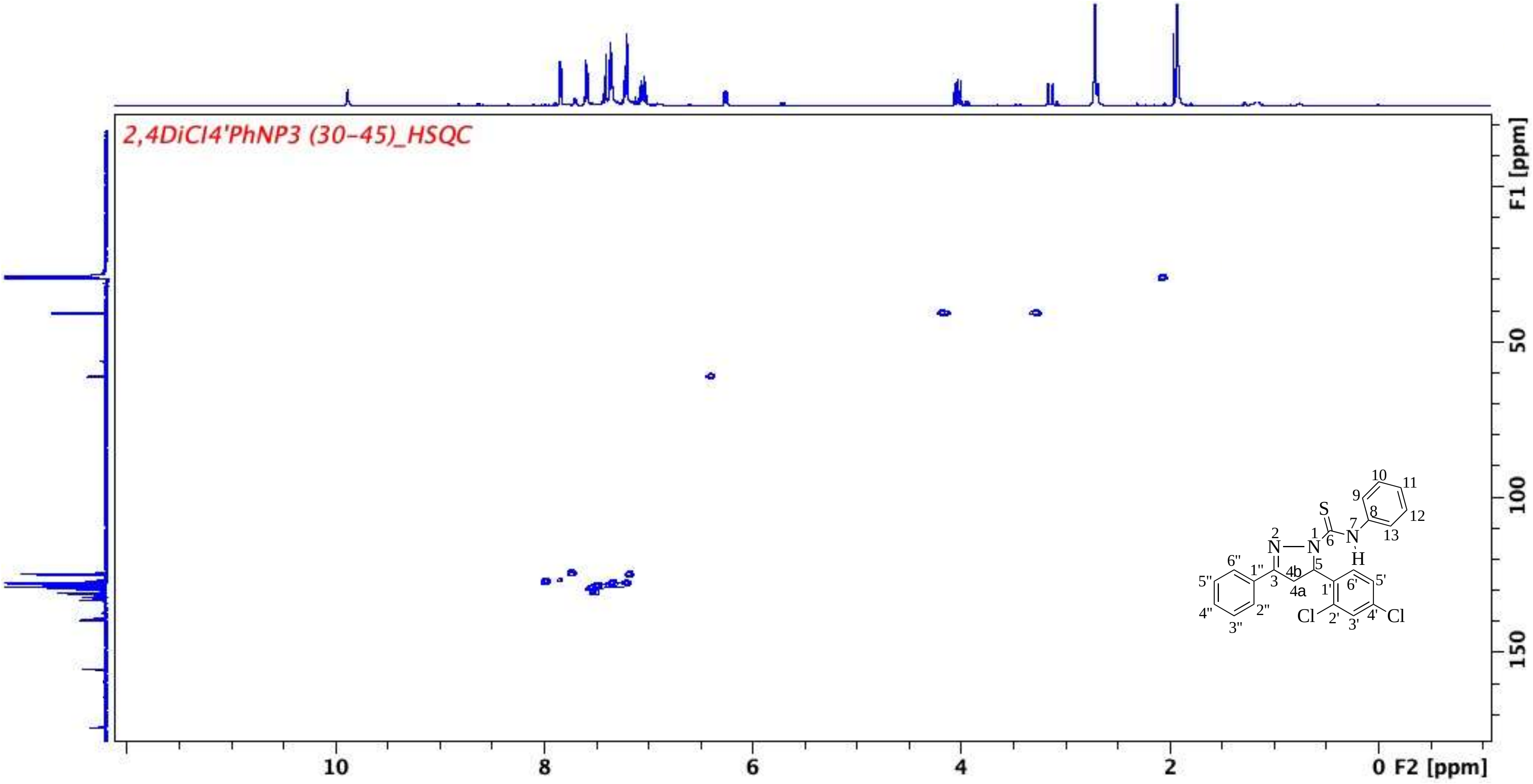

^{1}H-^{13}C HSQC NMR spectrum of compound 4e

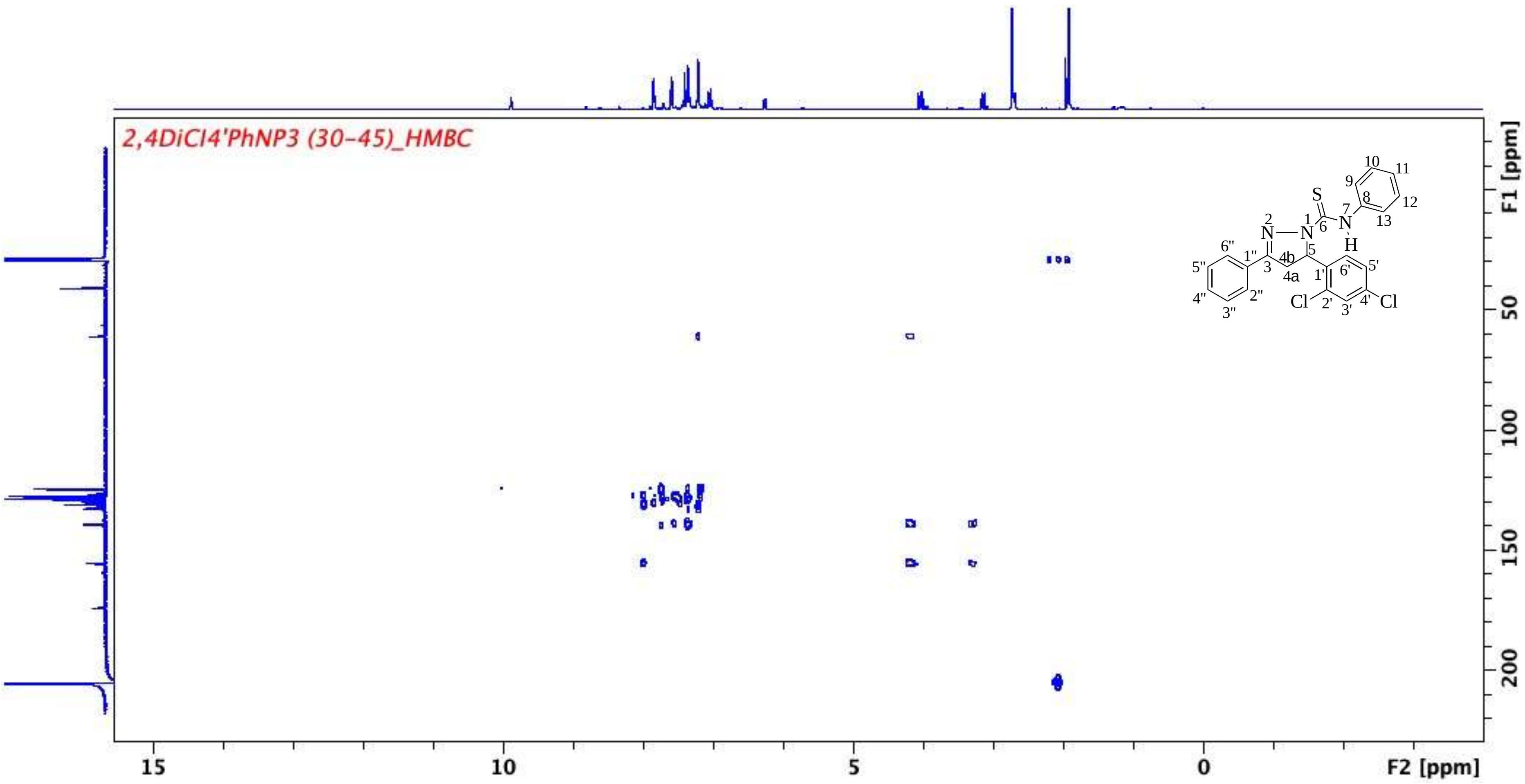

^{1}H-^{13}C HMBC NMR spectrum of compound 4e

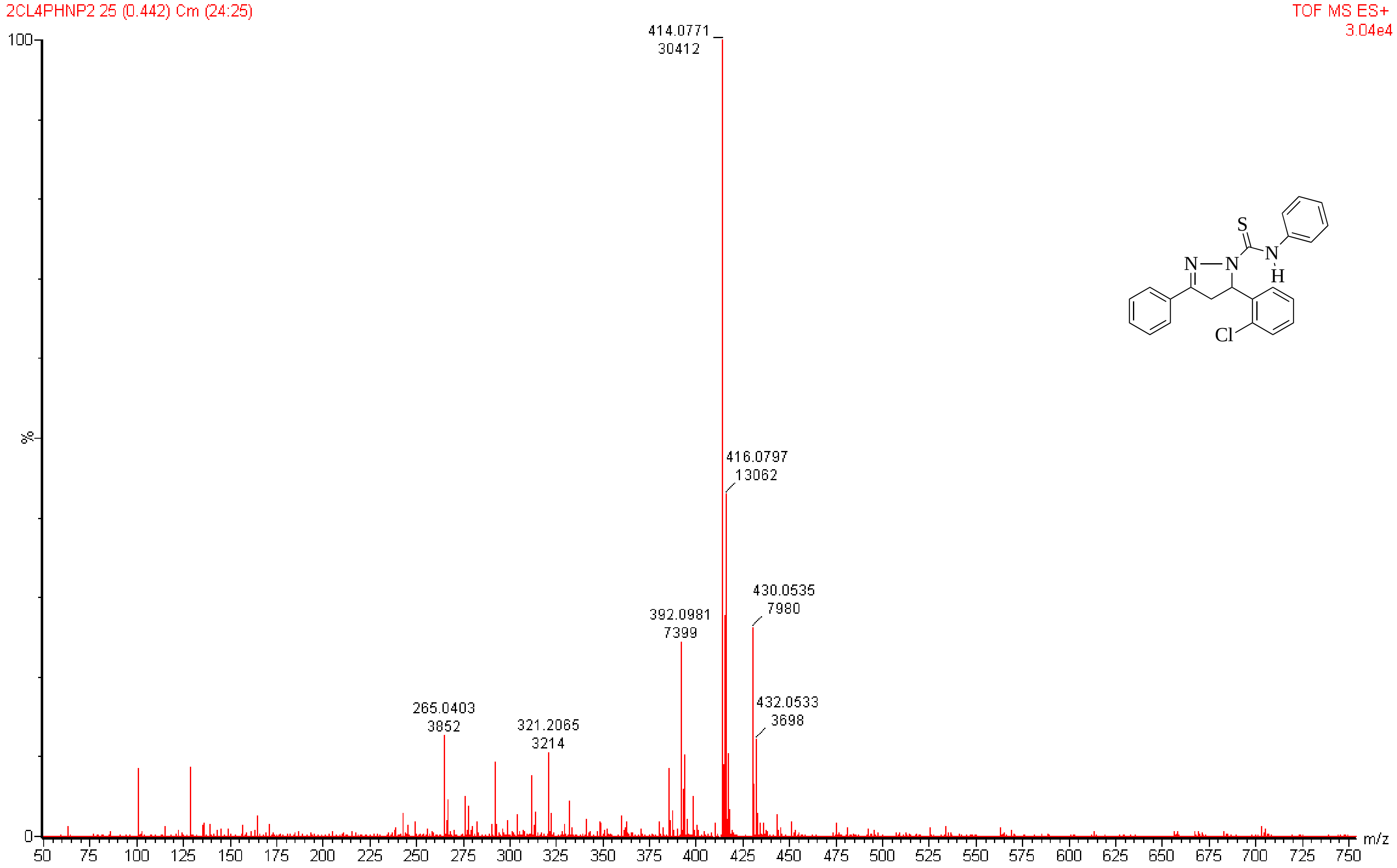

HRMS spectrum of compound **4f**

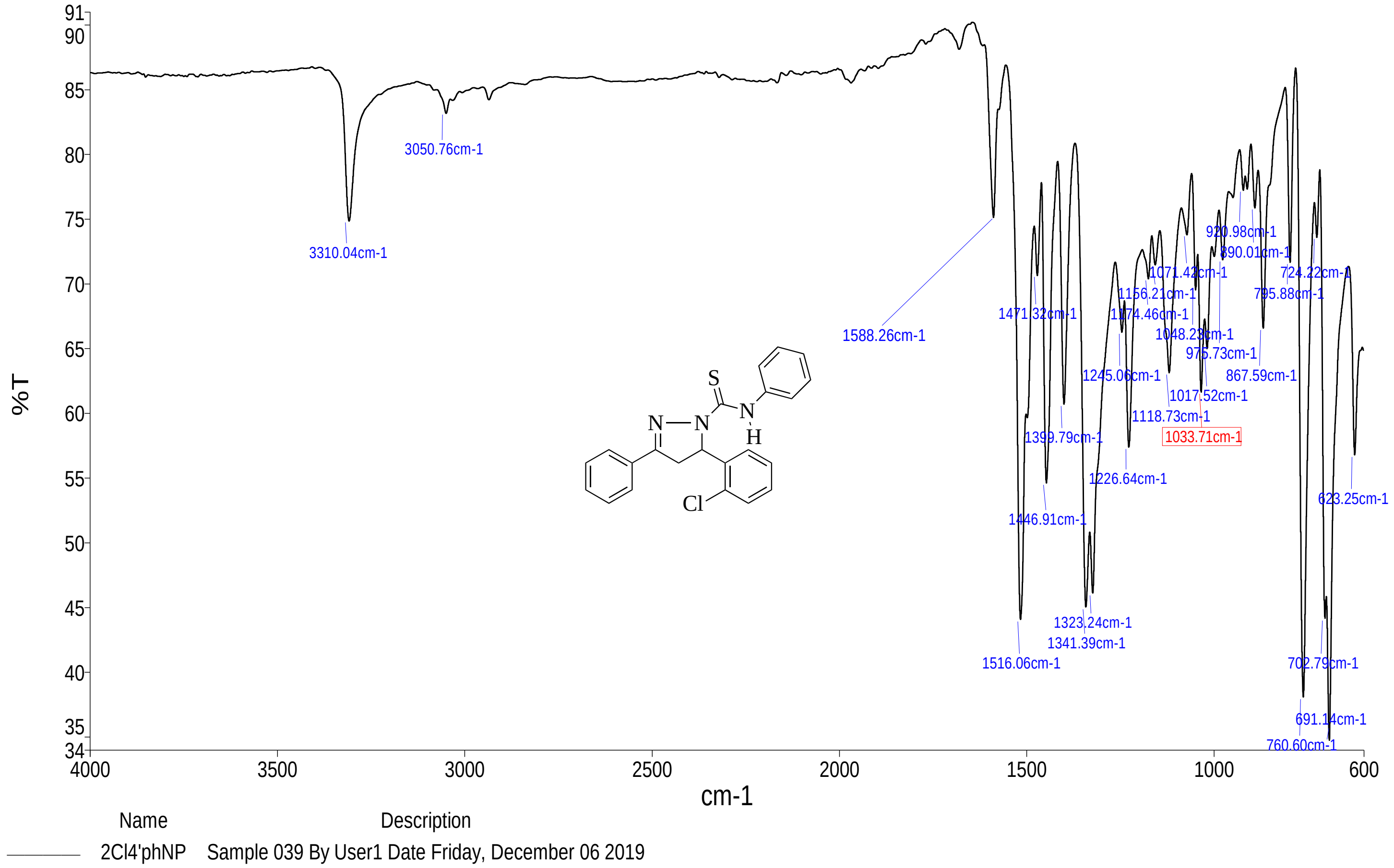

IR spectrum of compound **4f**

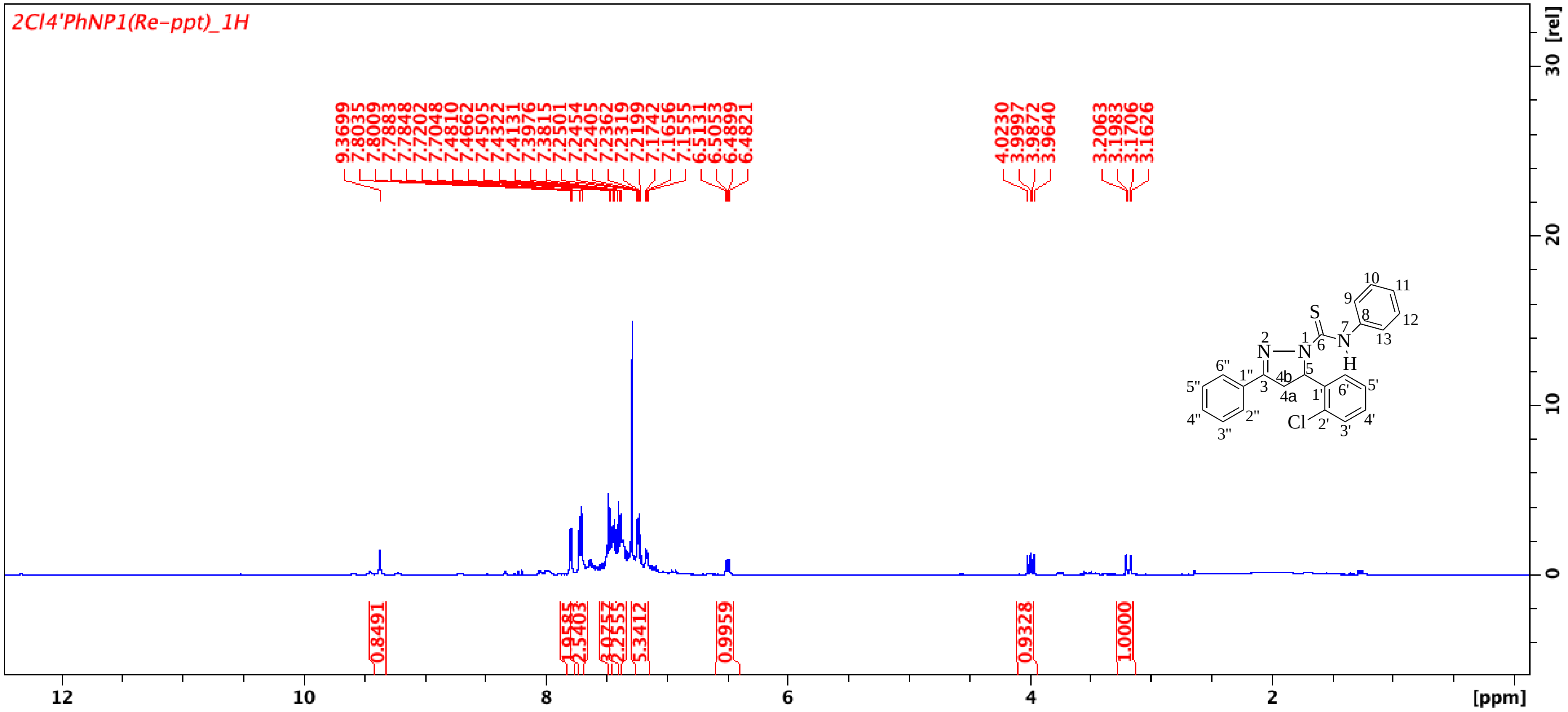

^{1}H NMR spectrum of compound **4f**

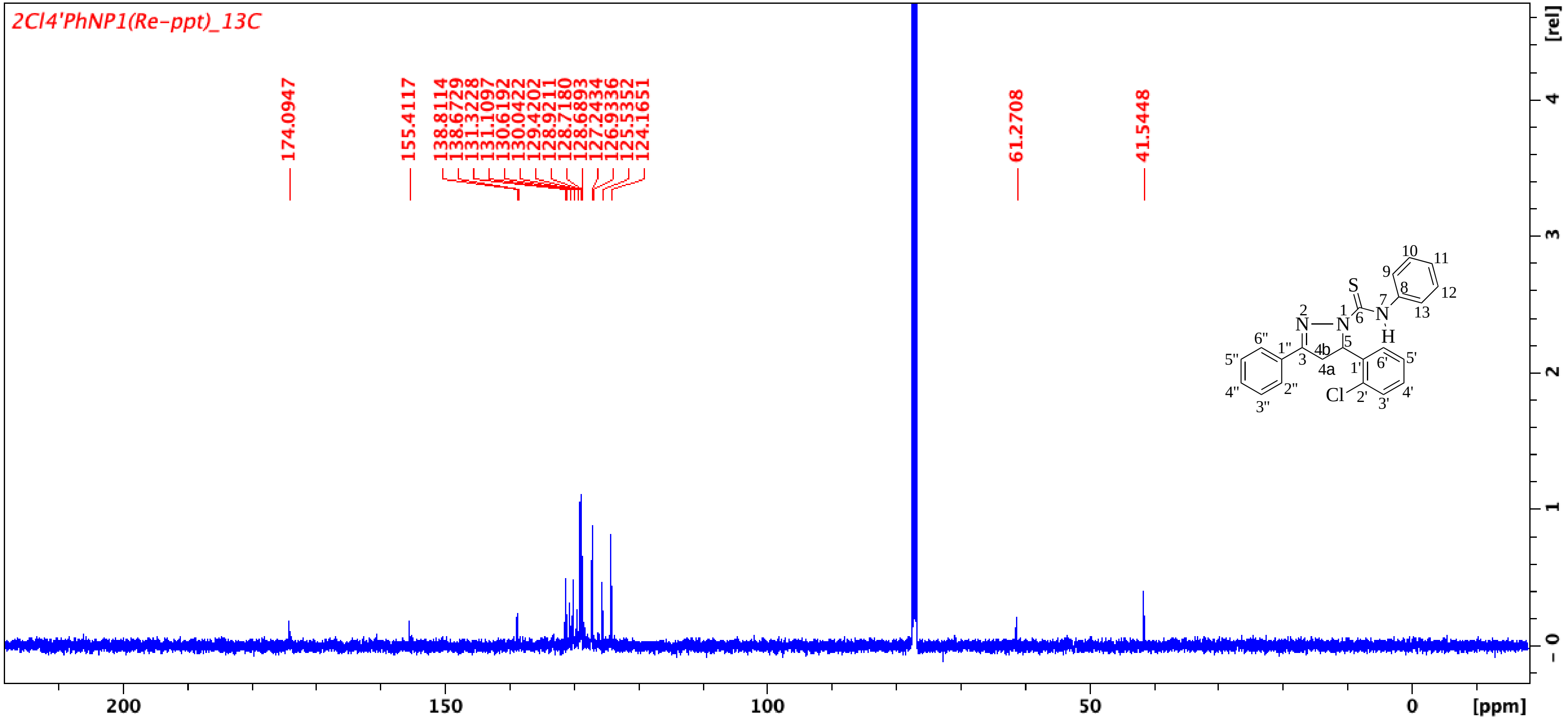

^{13}C NMR spectrum of compound **4f**

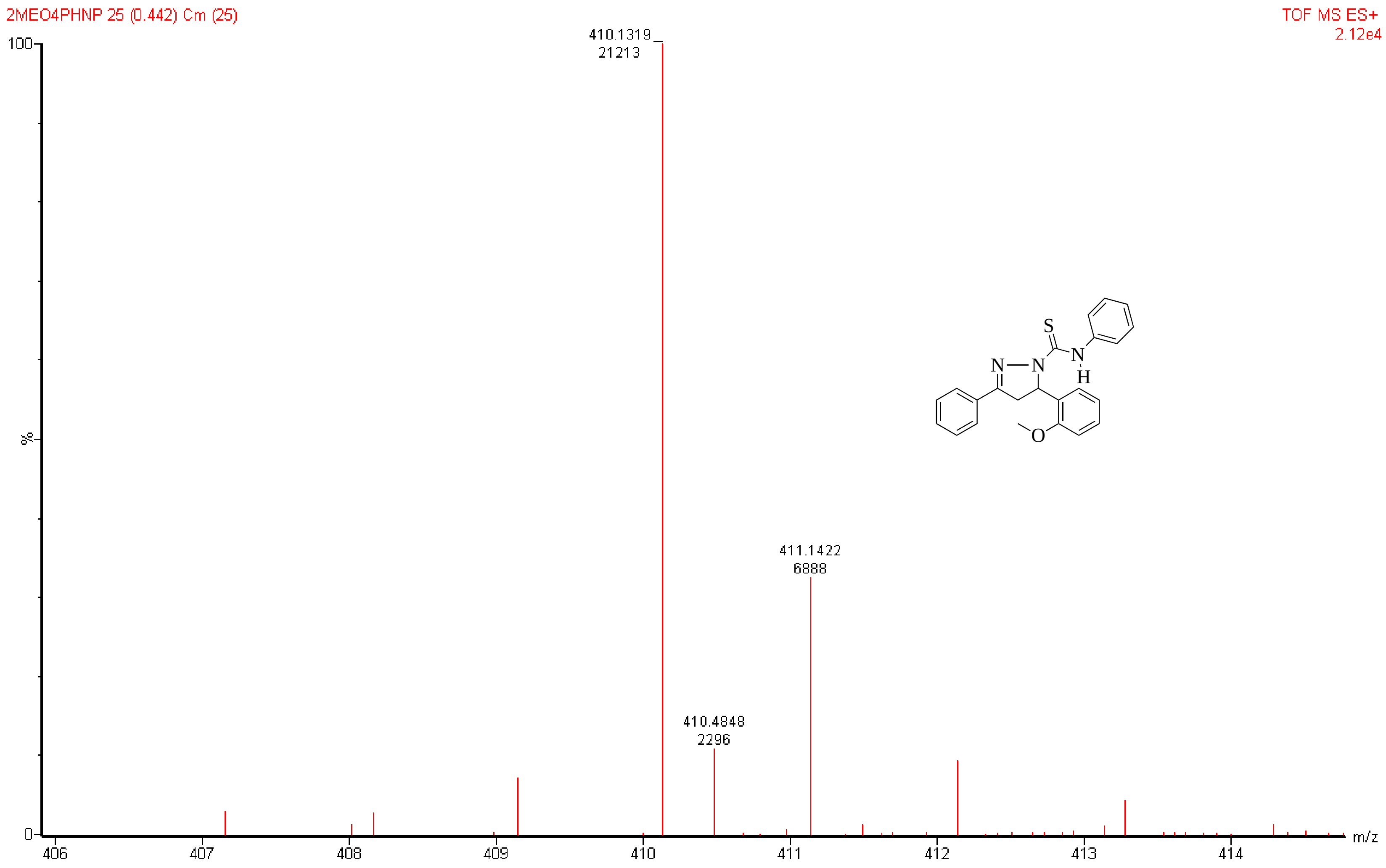

HRMS spectrum of compound **4g**

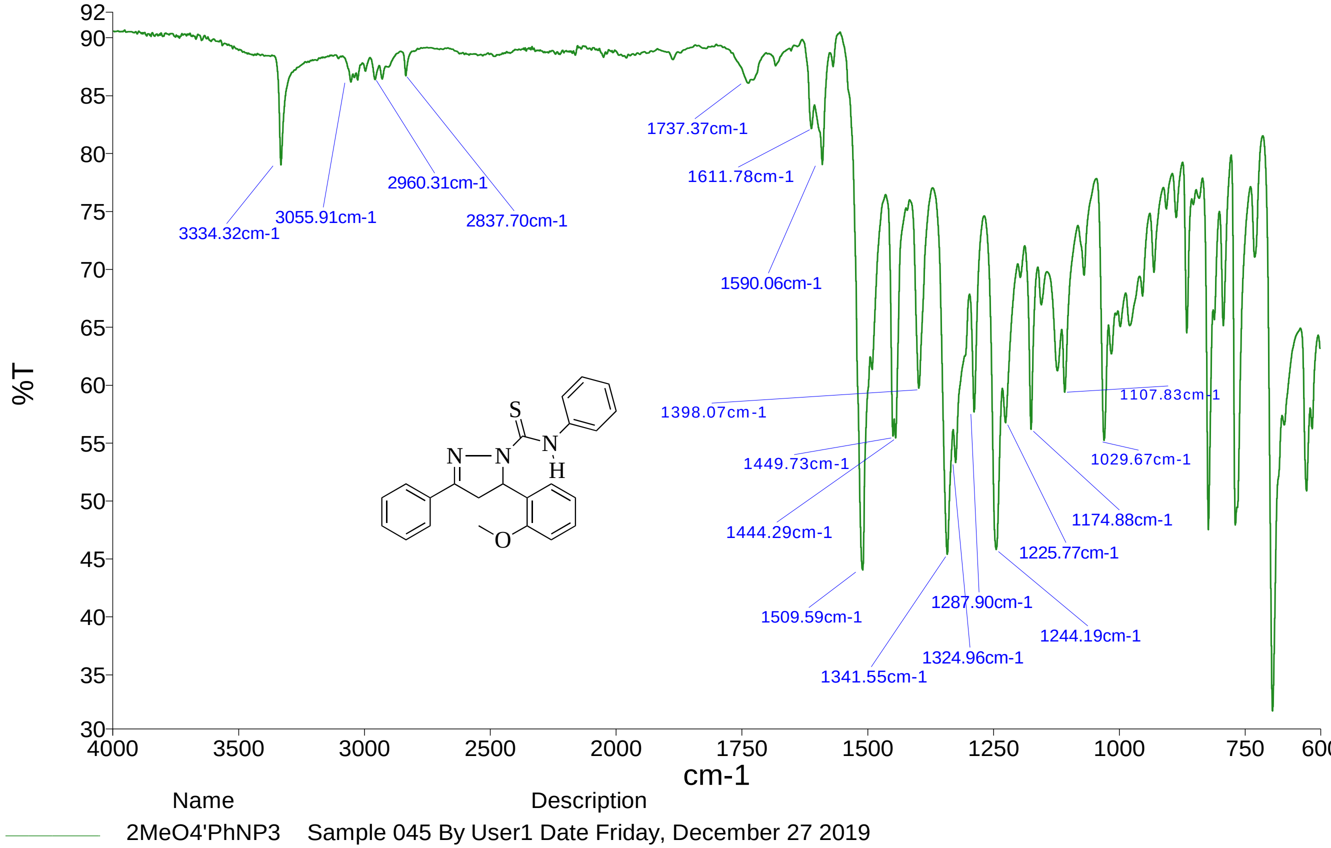

IR spectrum of compound **4g**

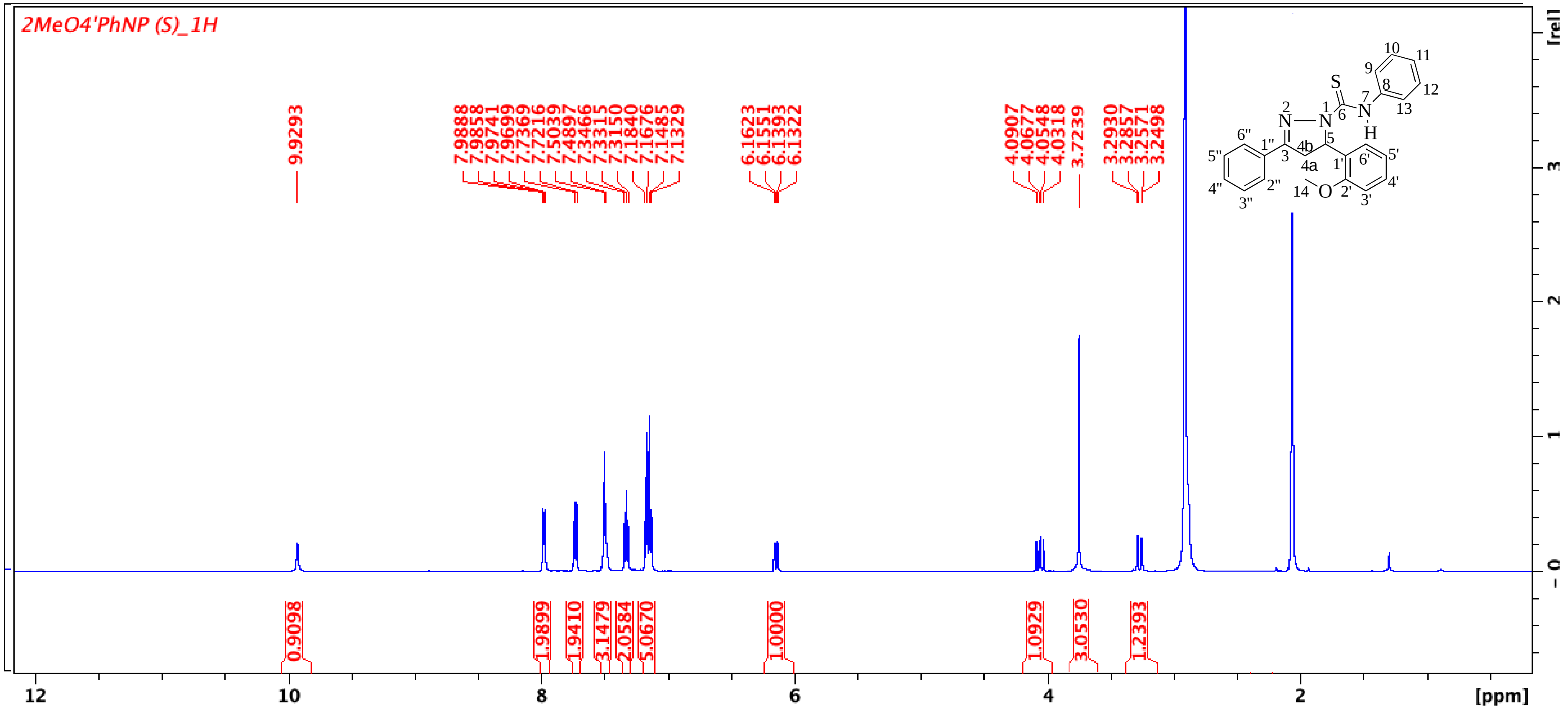

^{1}H NMR spectrum of compound **4g**

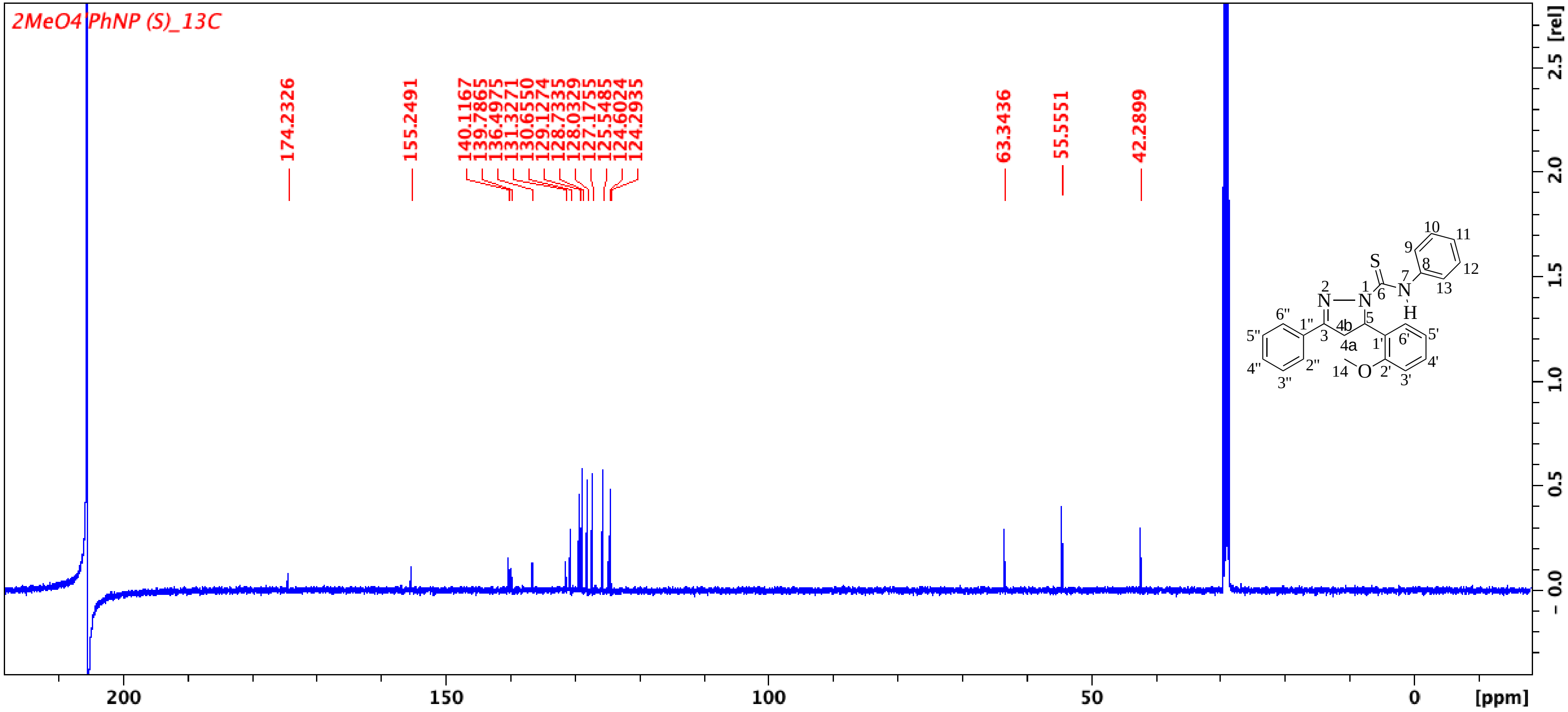

^{13}C NMR spectrum of compound **4g**

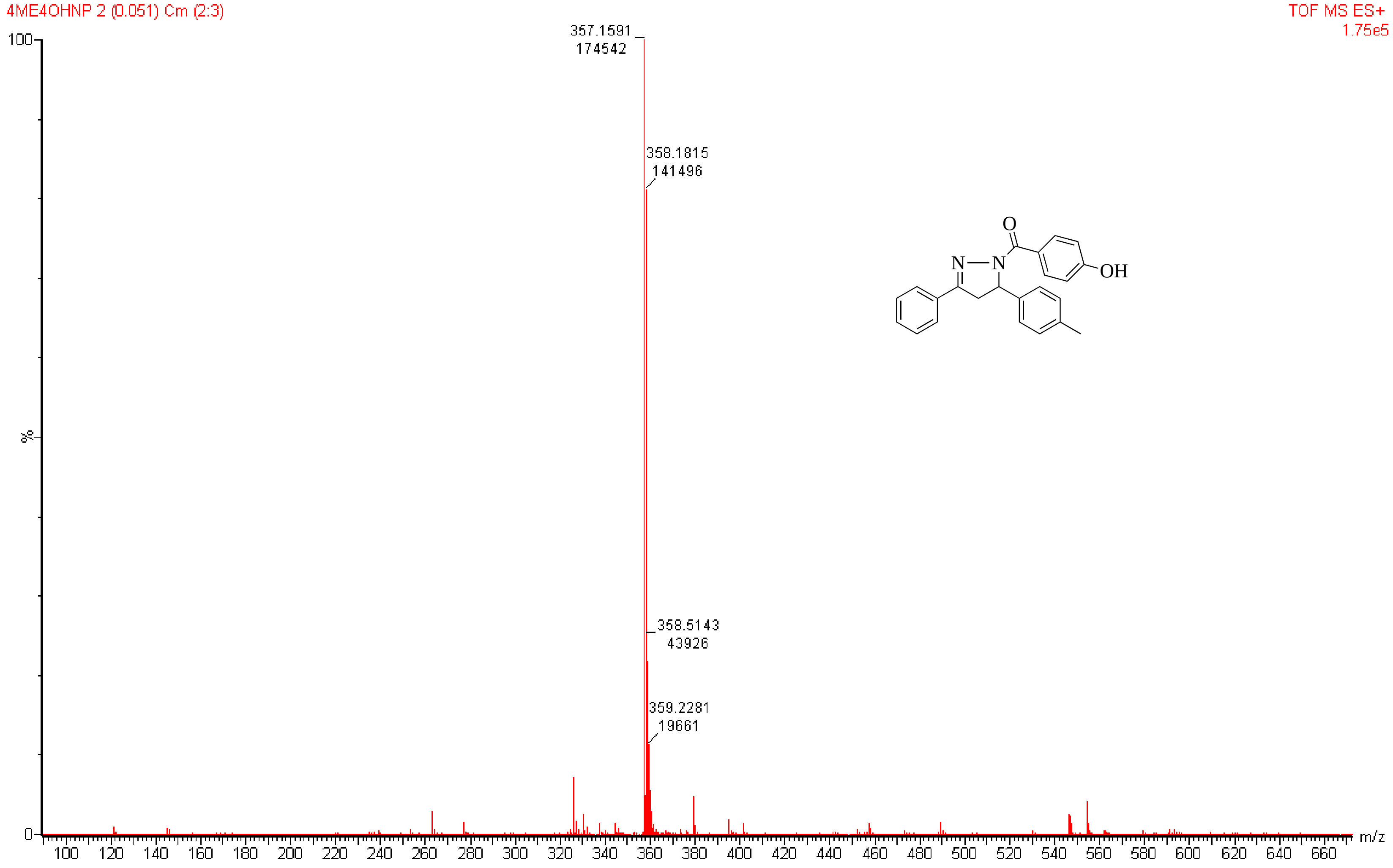

HRMS spectrum of compound **5a**

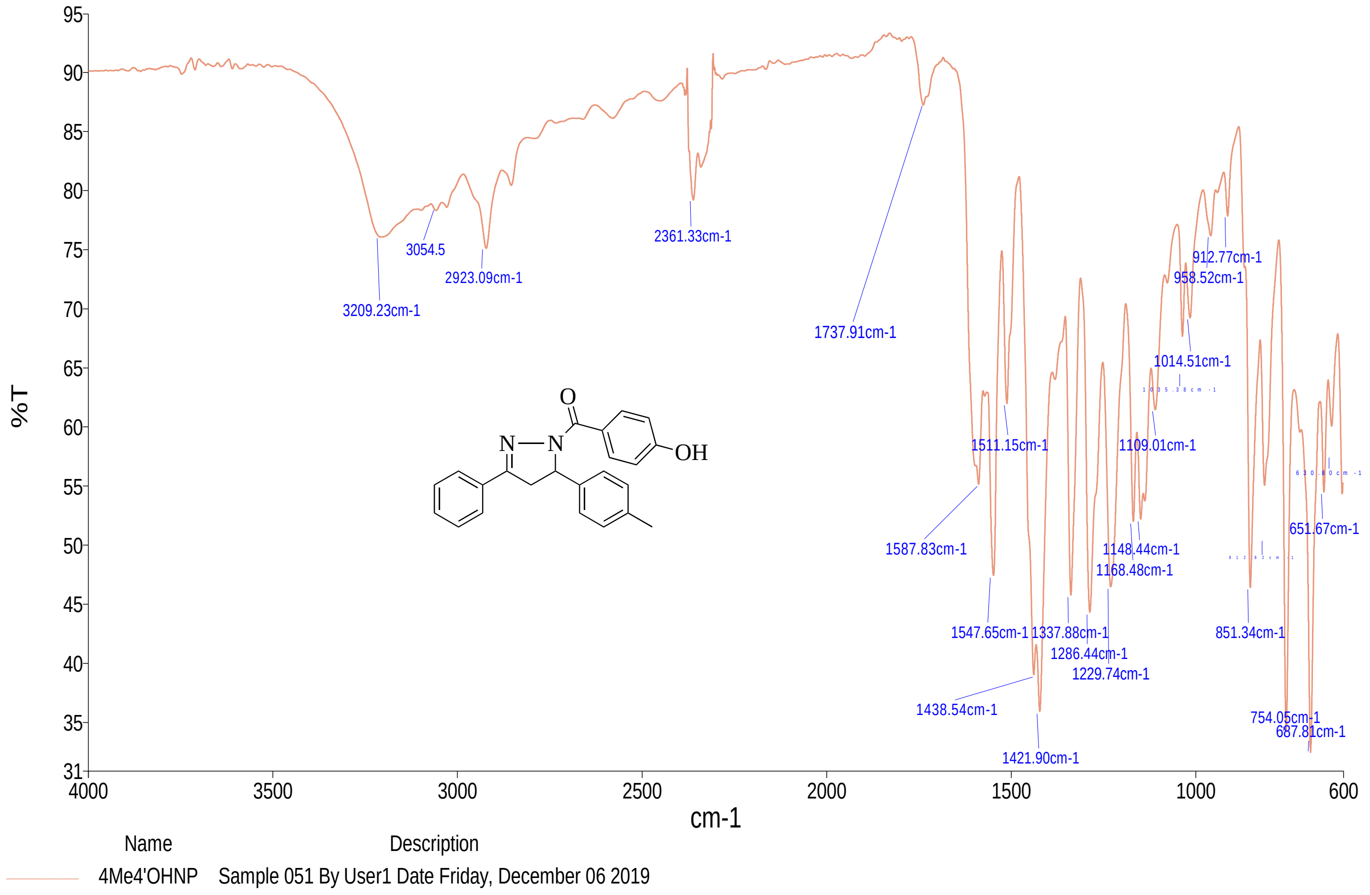

IR spectrum of compound **5a**

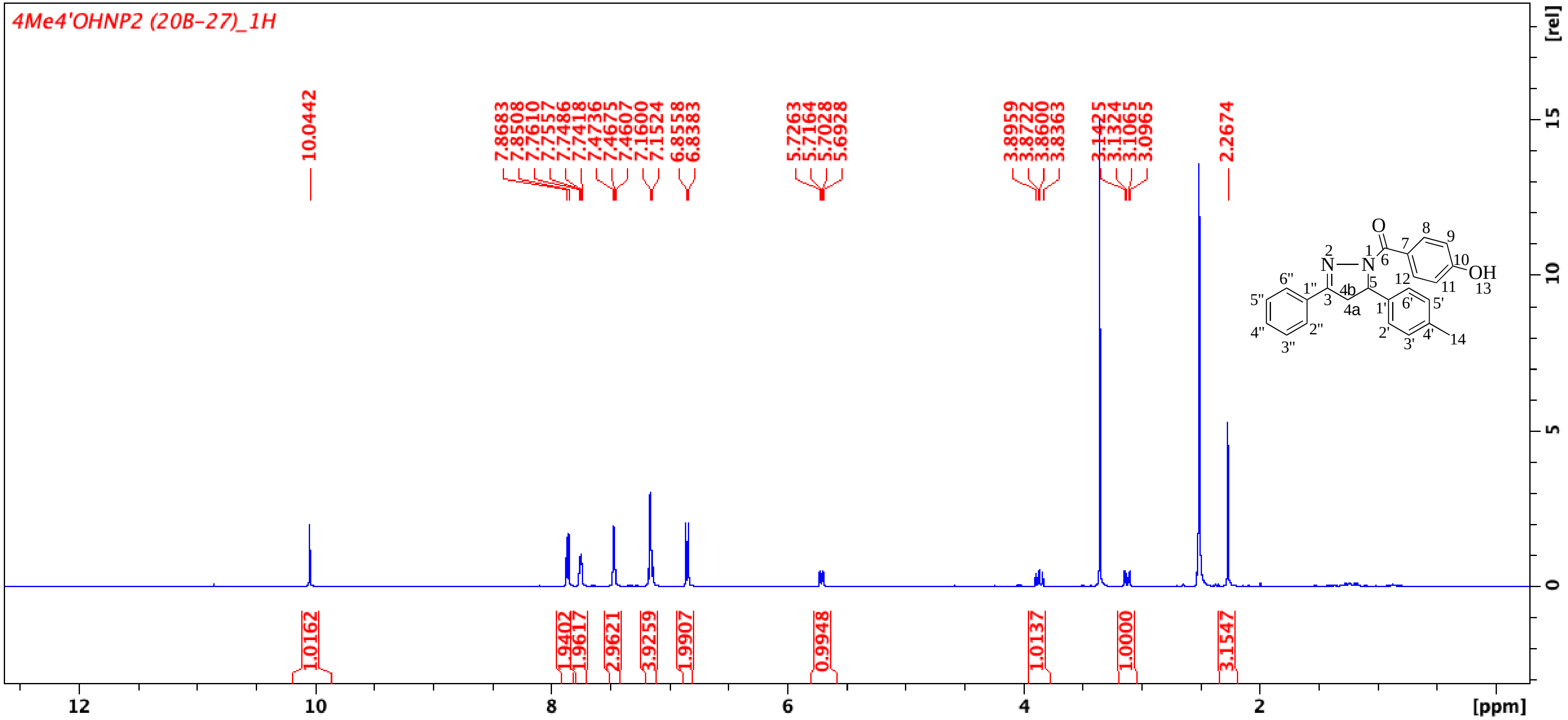

^{1}H NMR spectrum of compound **5a**

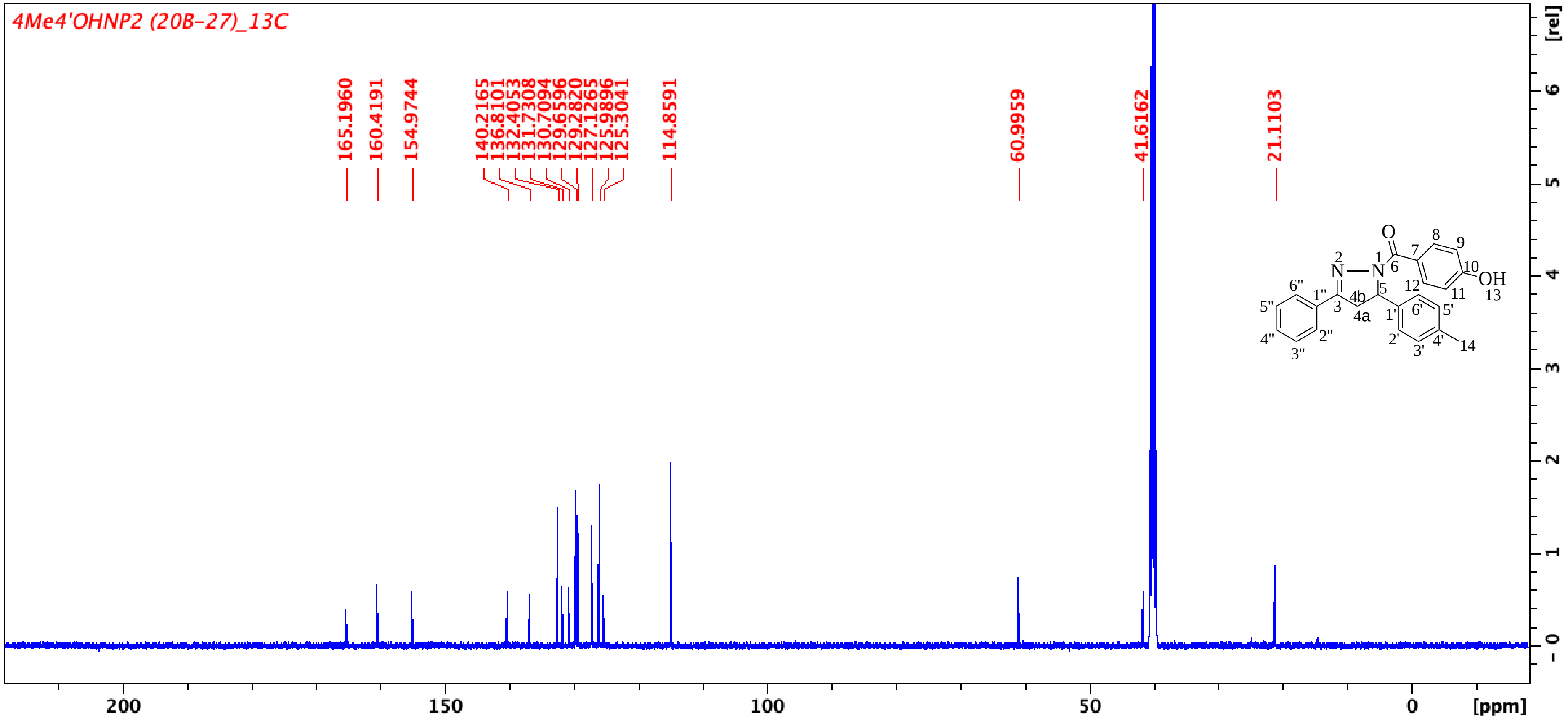

^{13}C NMR spectrum of compound **5a**

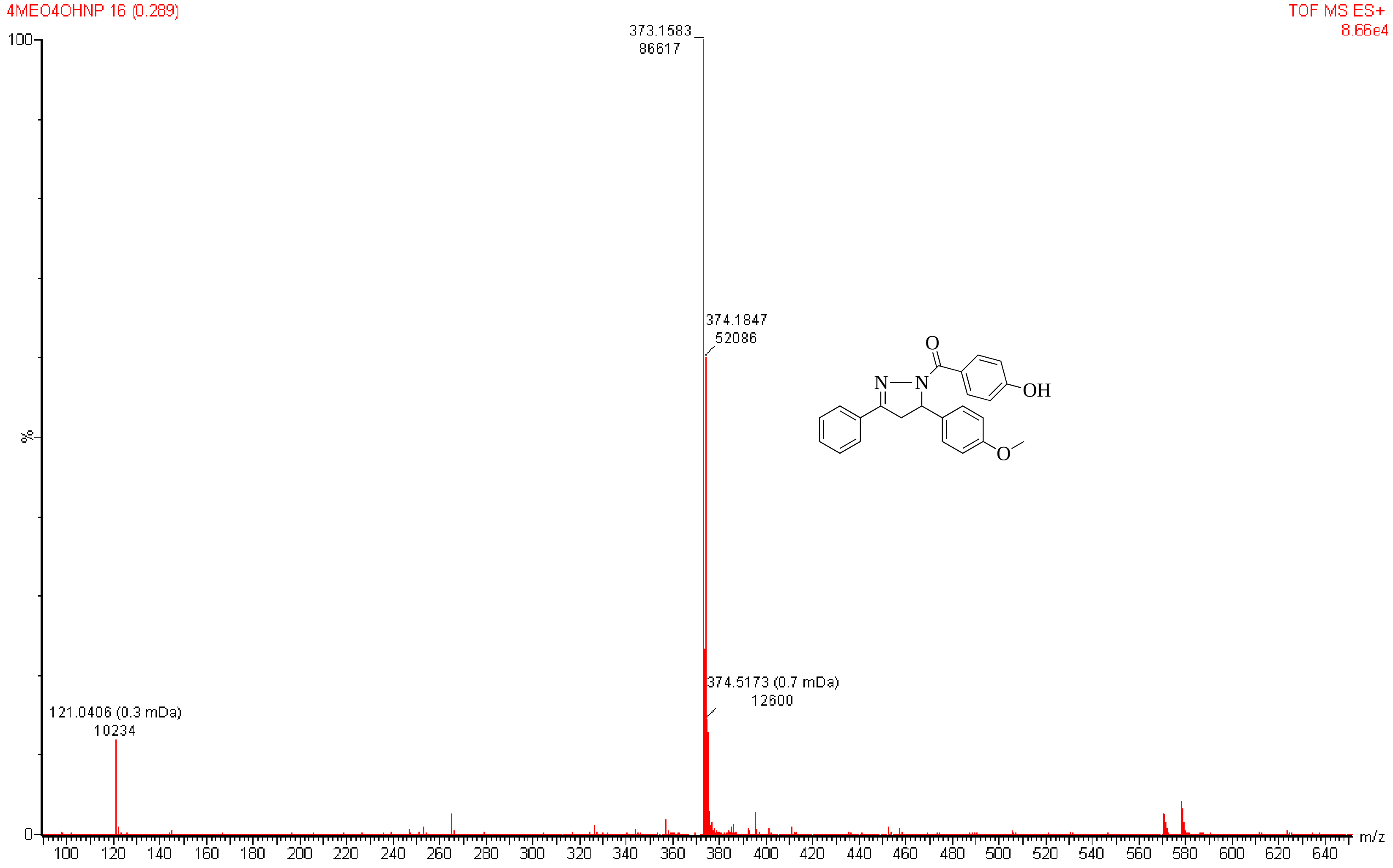

HRMS spectrum of compound **5b**

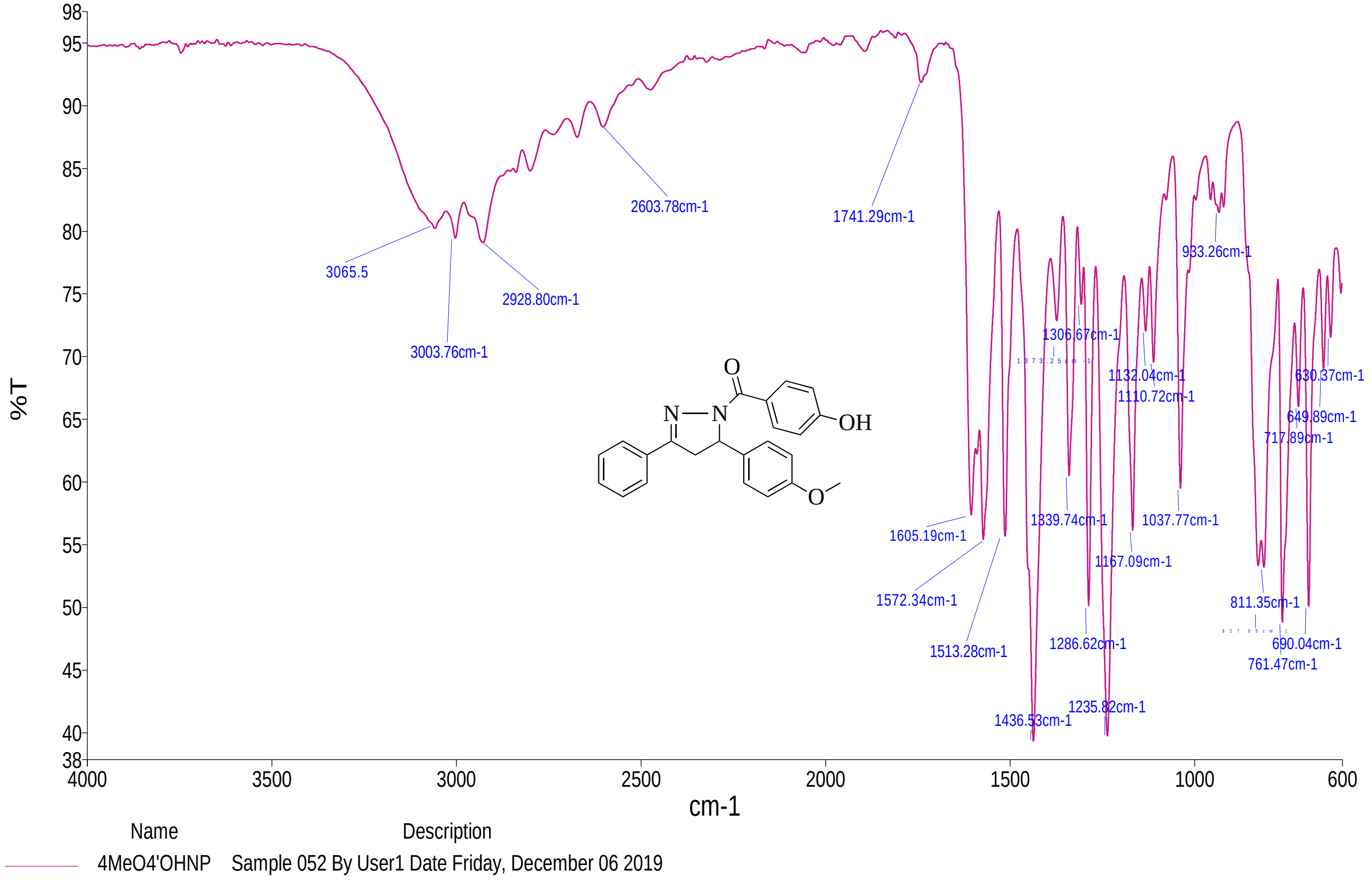

IR spectrum of compound **5b**

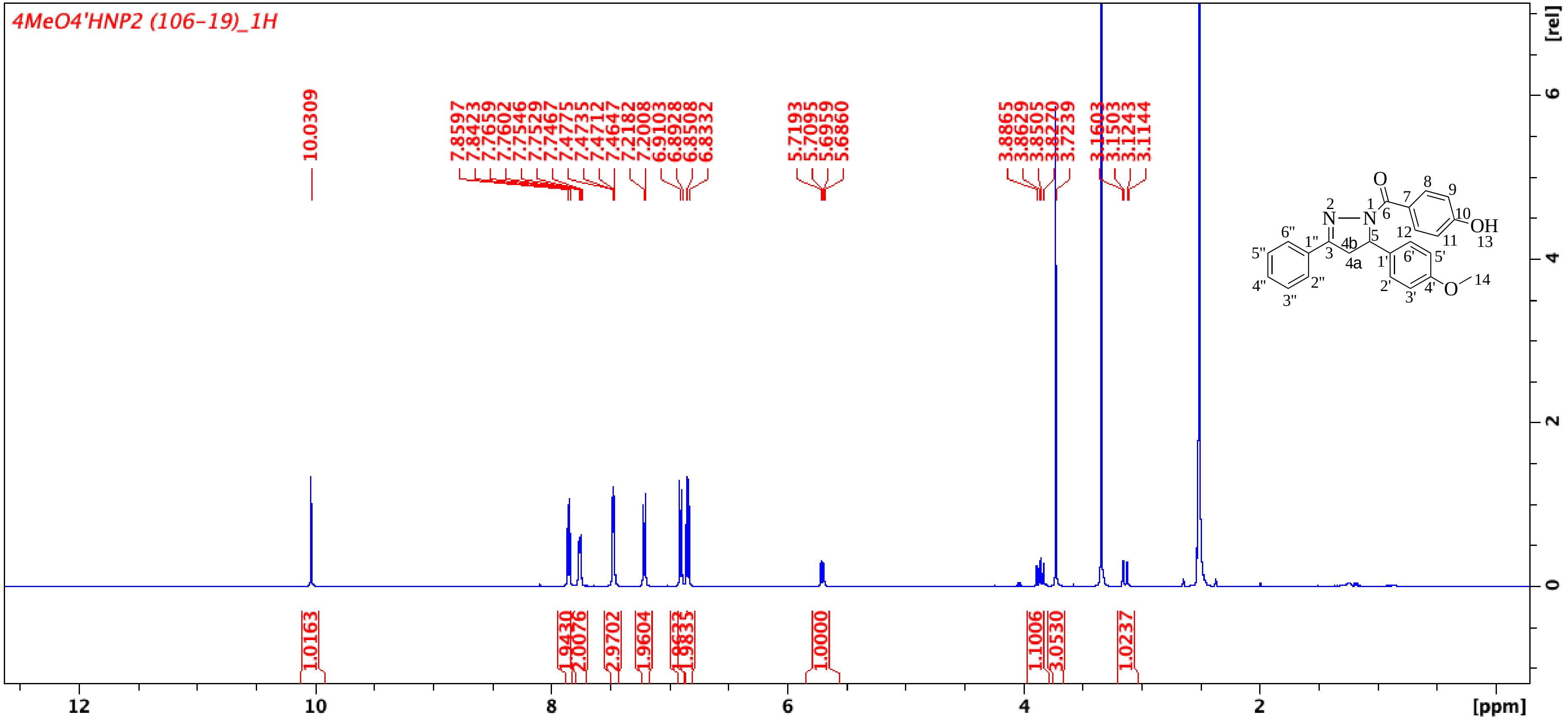

^{1}H NMR spectrum of compound **5b**

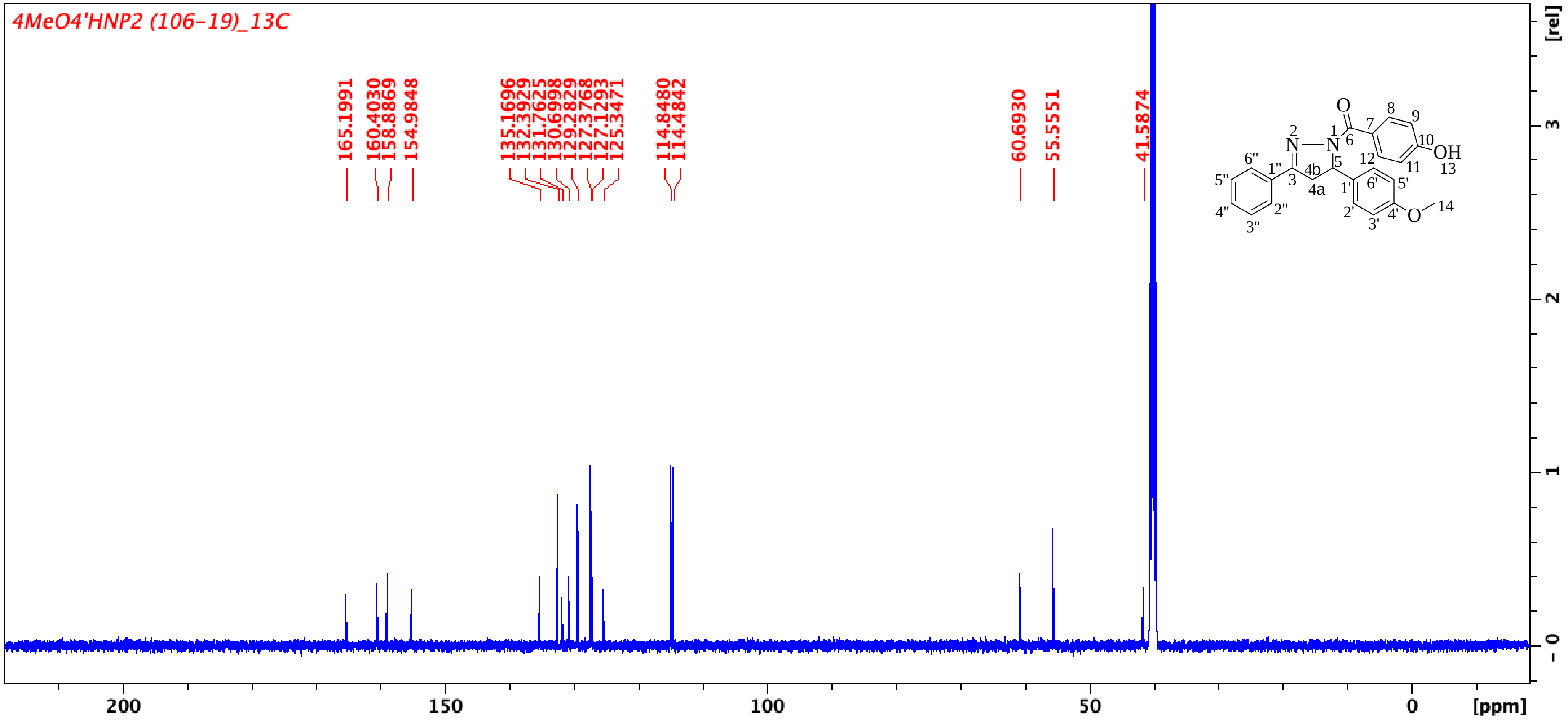

^{13}C NMR spectrum of compound **5b**

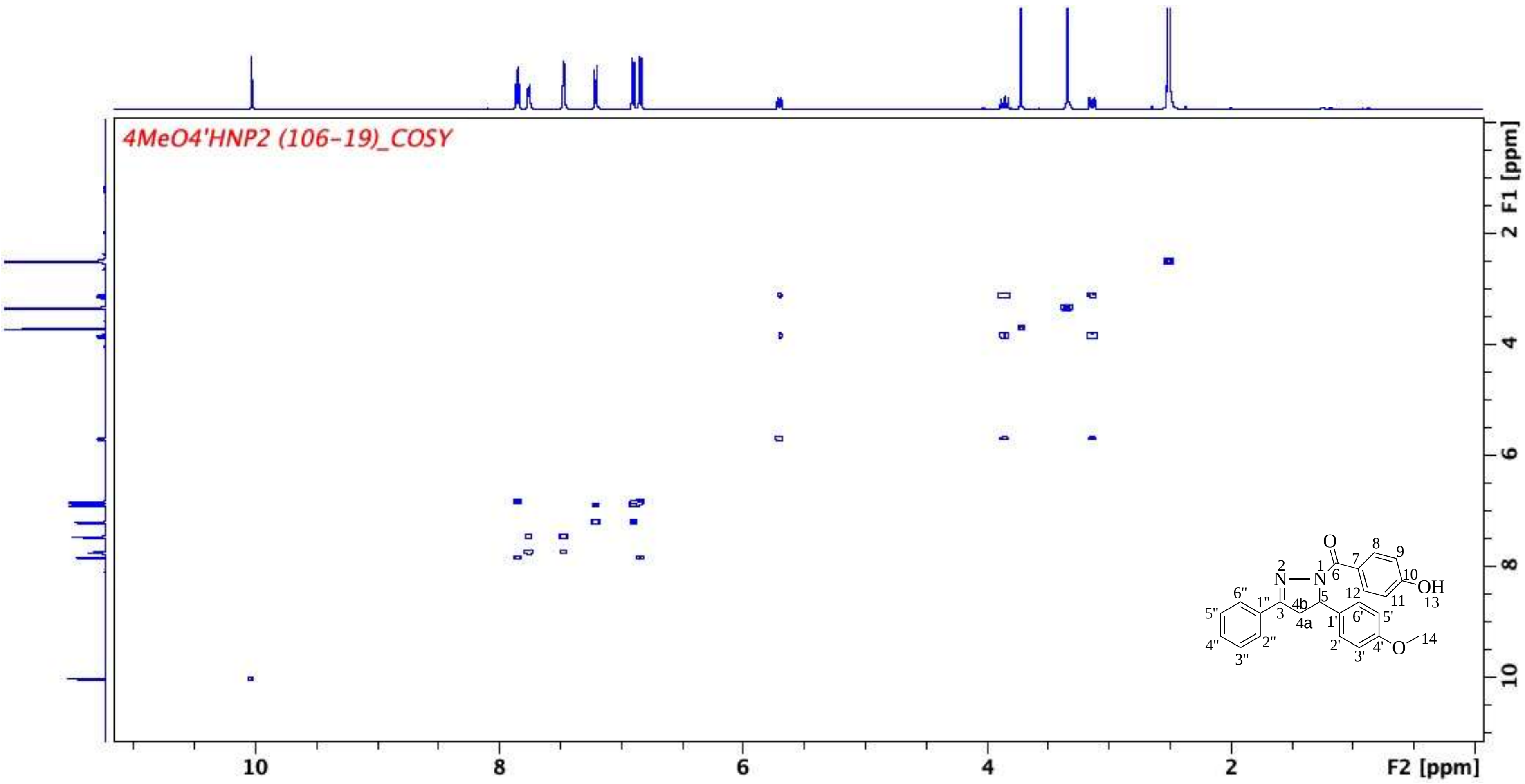

^{1}H-^{1}H COSY NMR spectrum of compound 5b

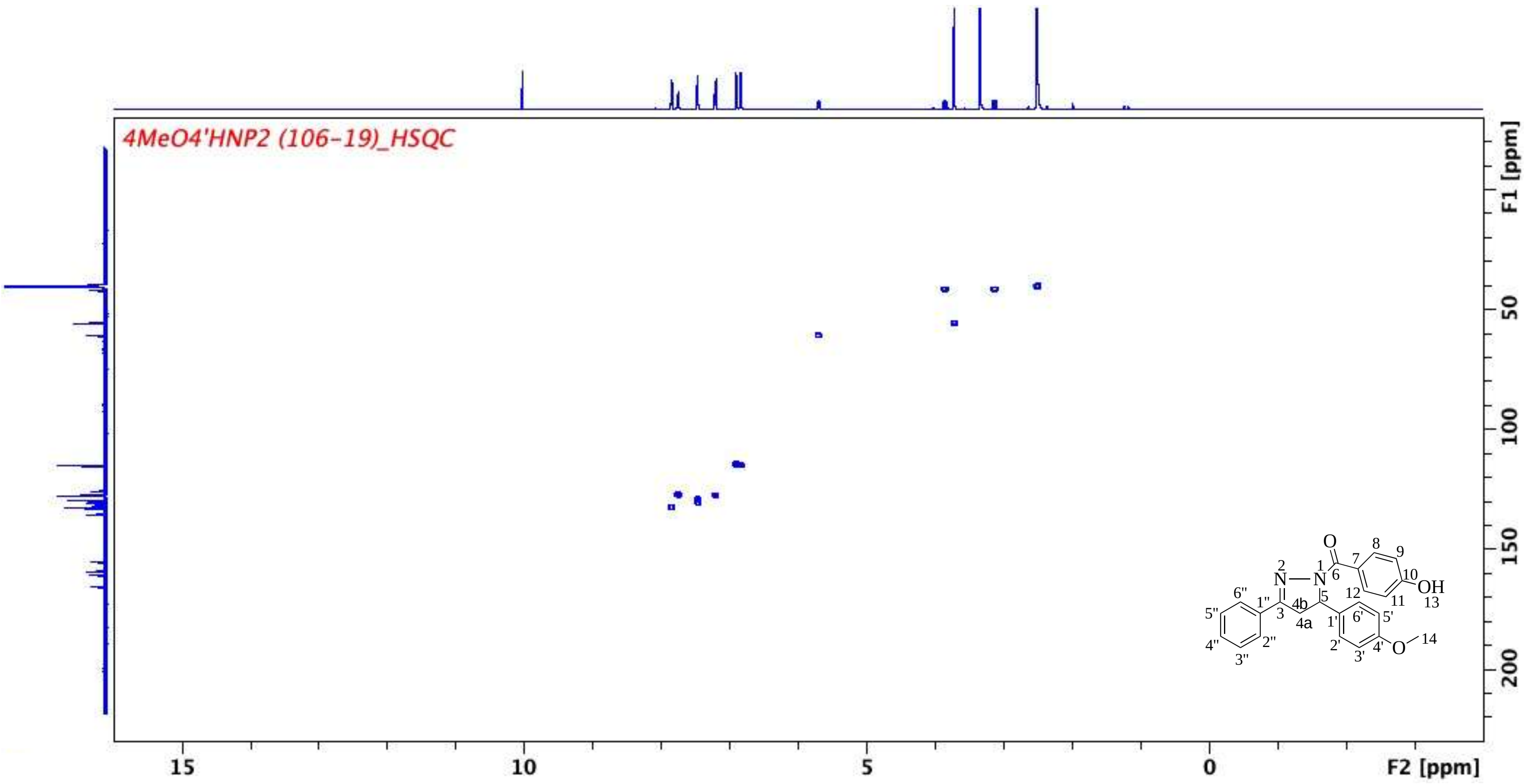

^{1}H-^{13}C HSQC NMR spectrum of compound 5b

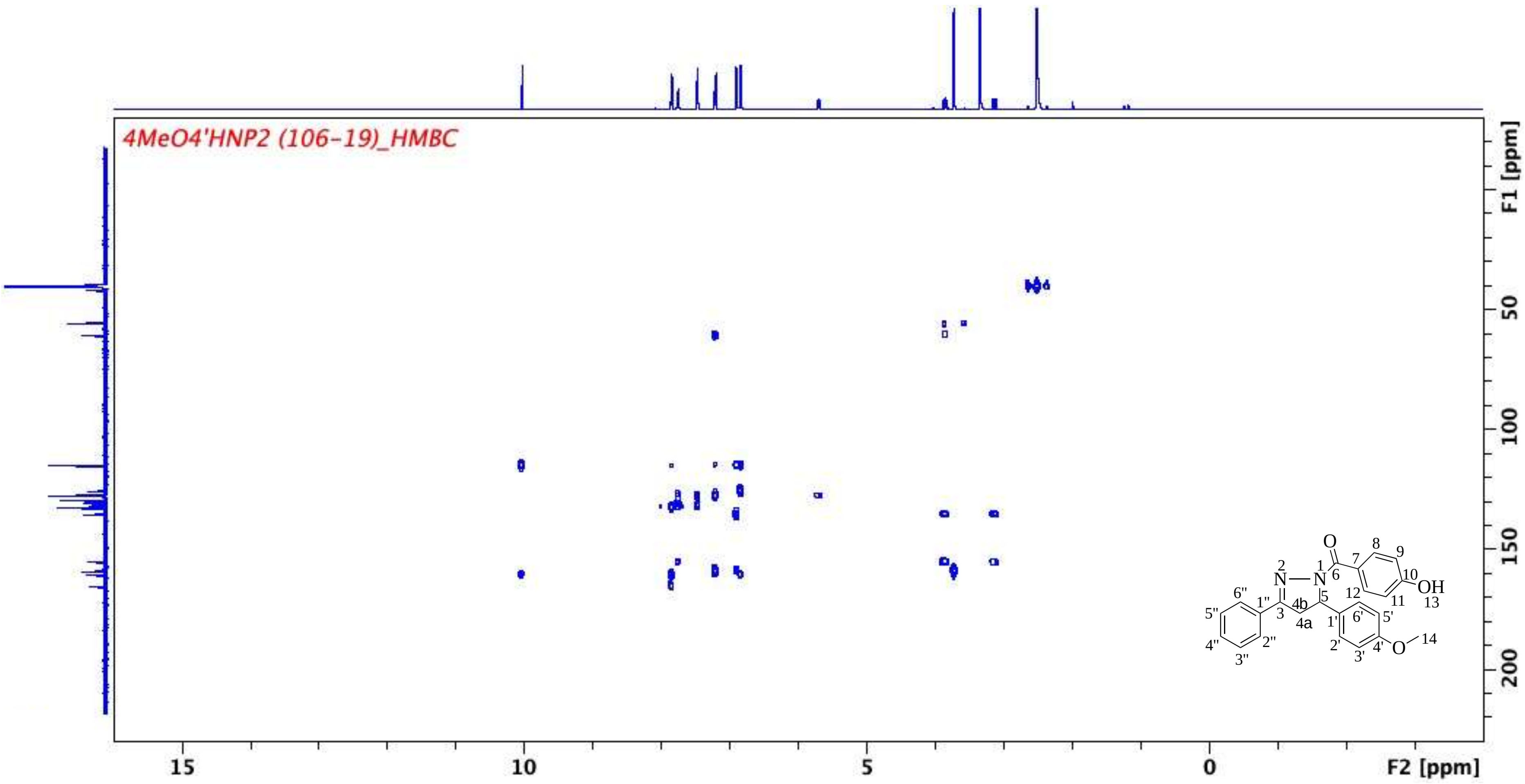

^{1}H-^{13}C HMBC NMR spectrum of compound 5b

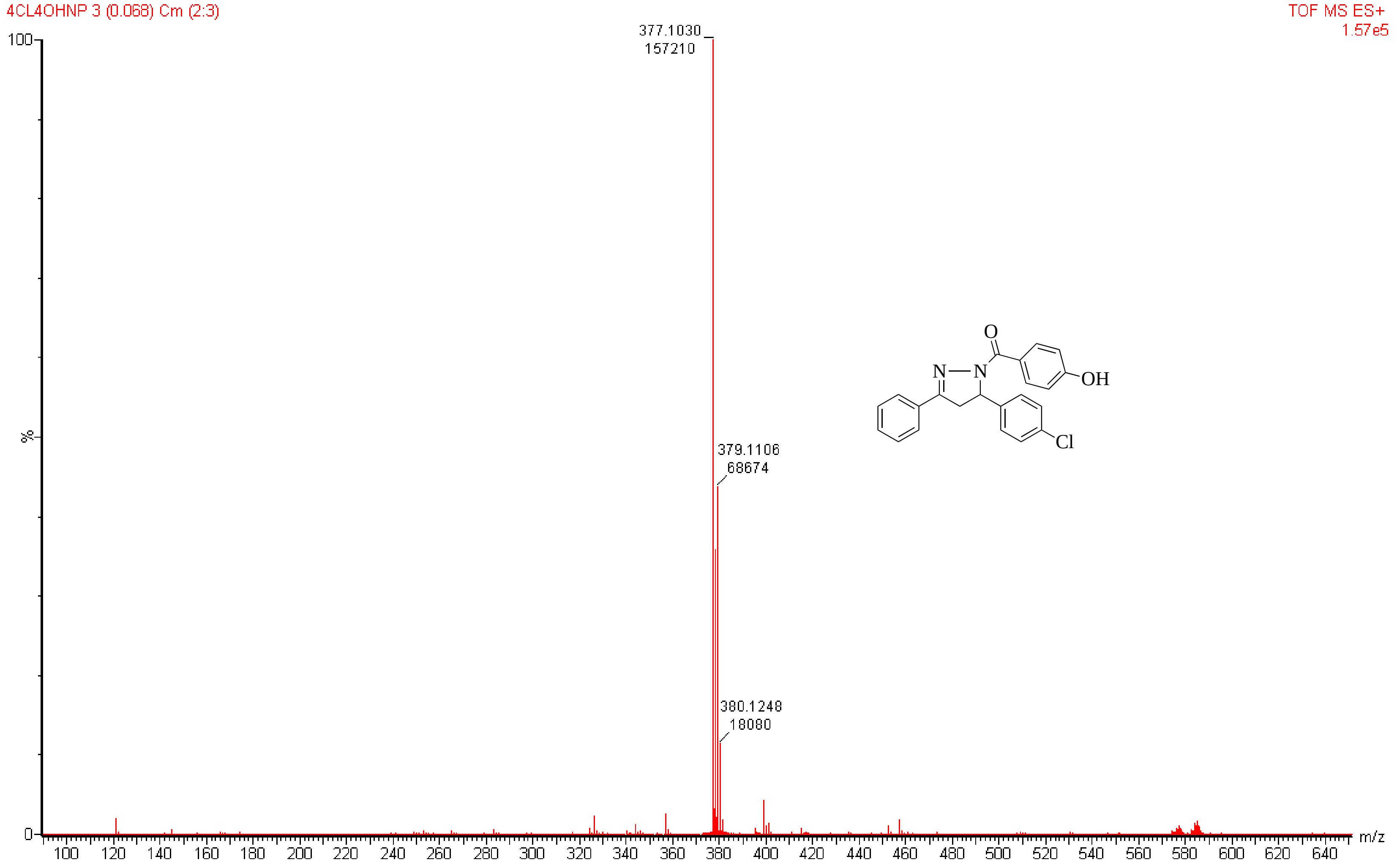

HRMS spectrum of compound **5c**

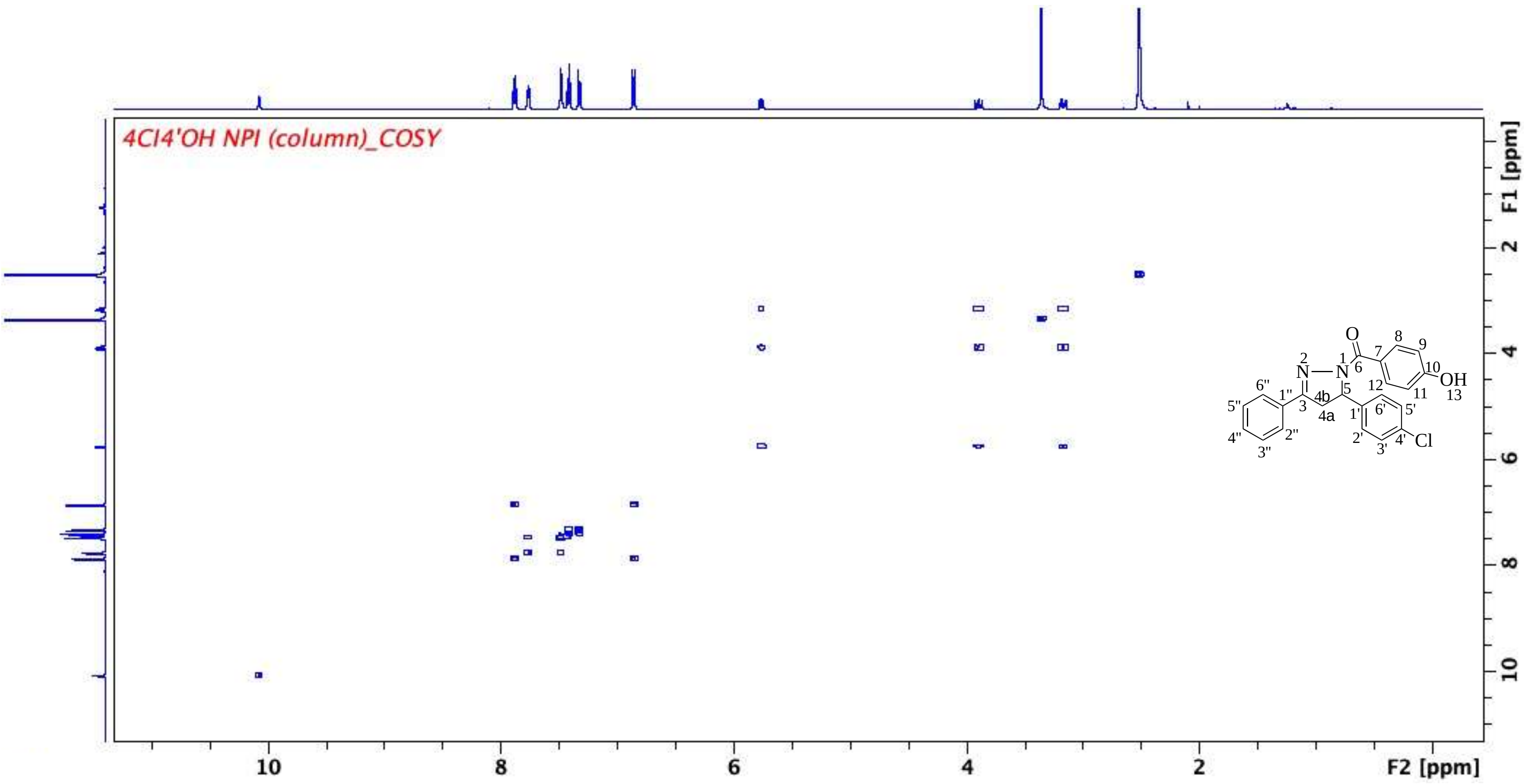

^{1}H-^{1}H COSY NMR spectrum of compound 5c

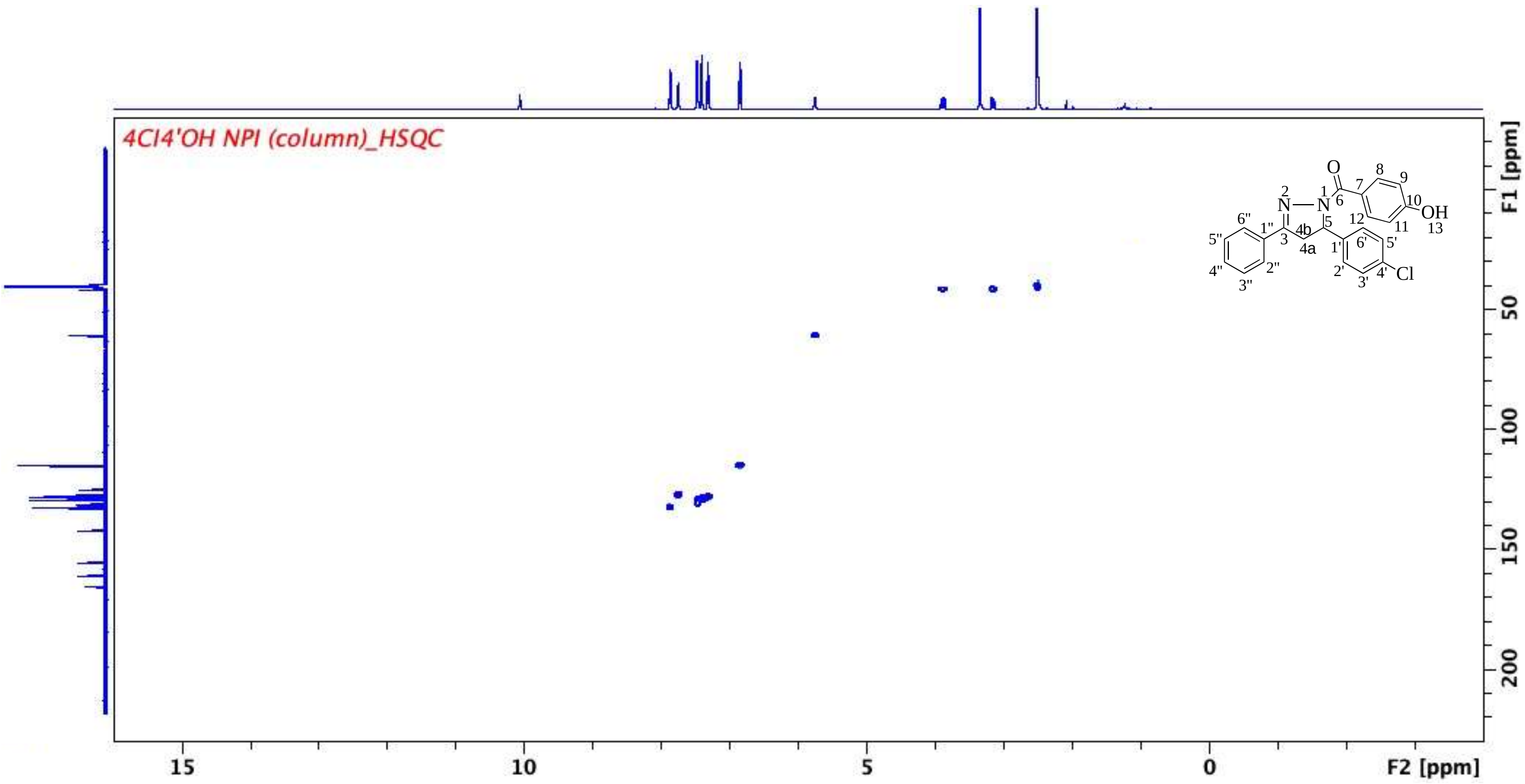

^{1}H-^{13}C HSQC NMR spectrum of compound 5c

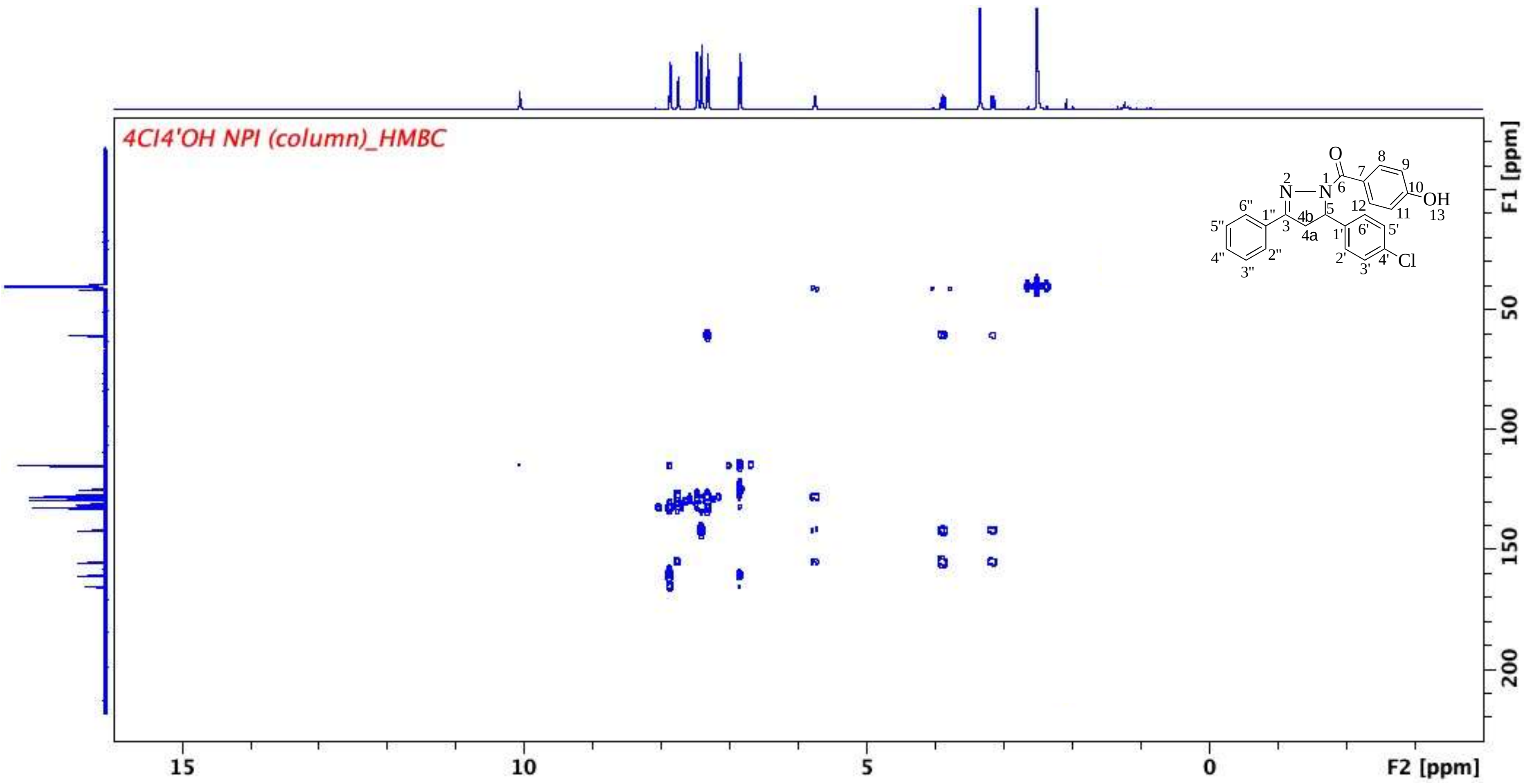

^{1}H-^{13}C HMBC NMR spectrum of compound 5c

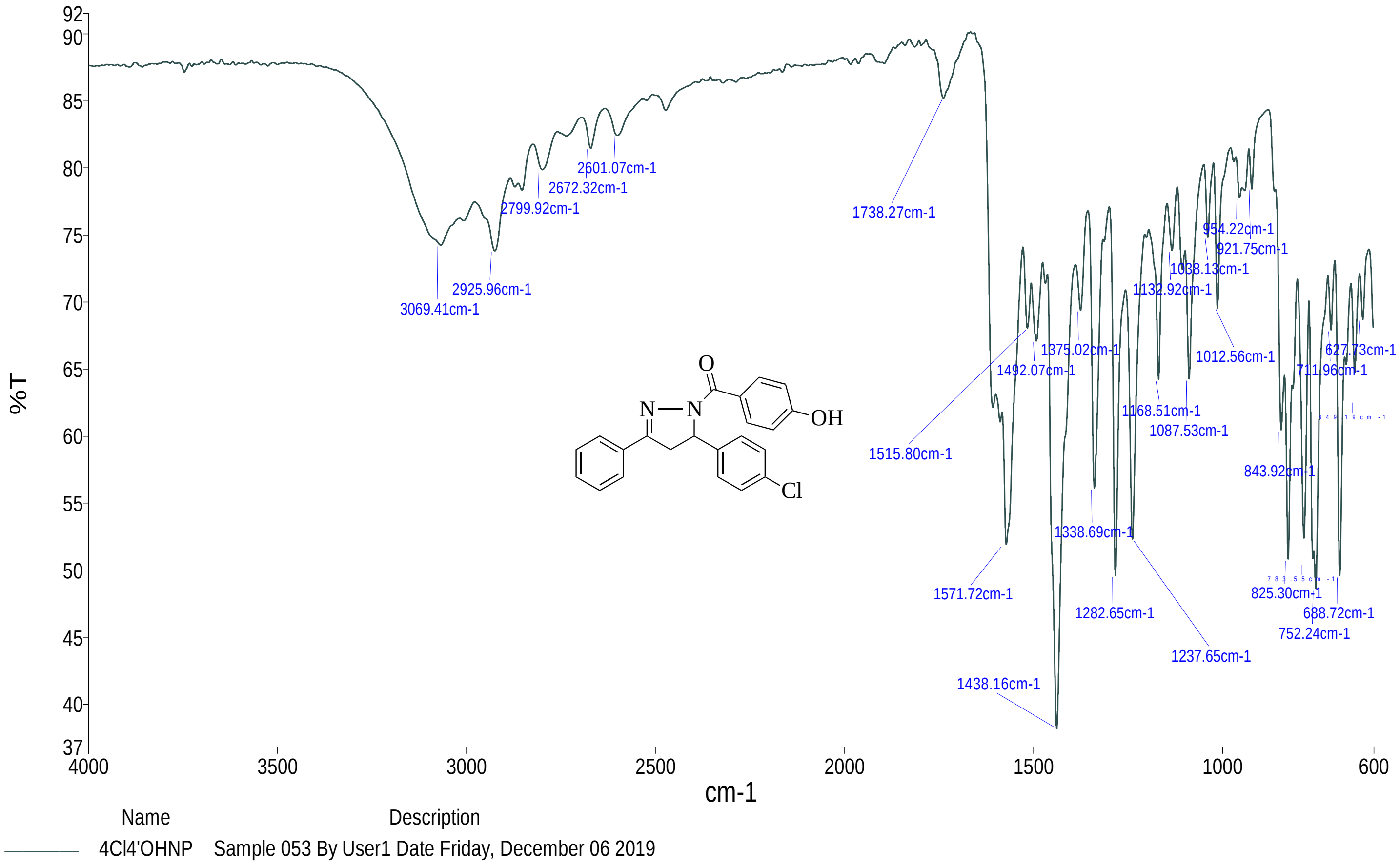

IR spectrum of compound **5c**

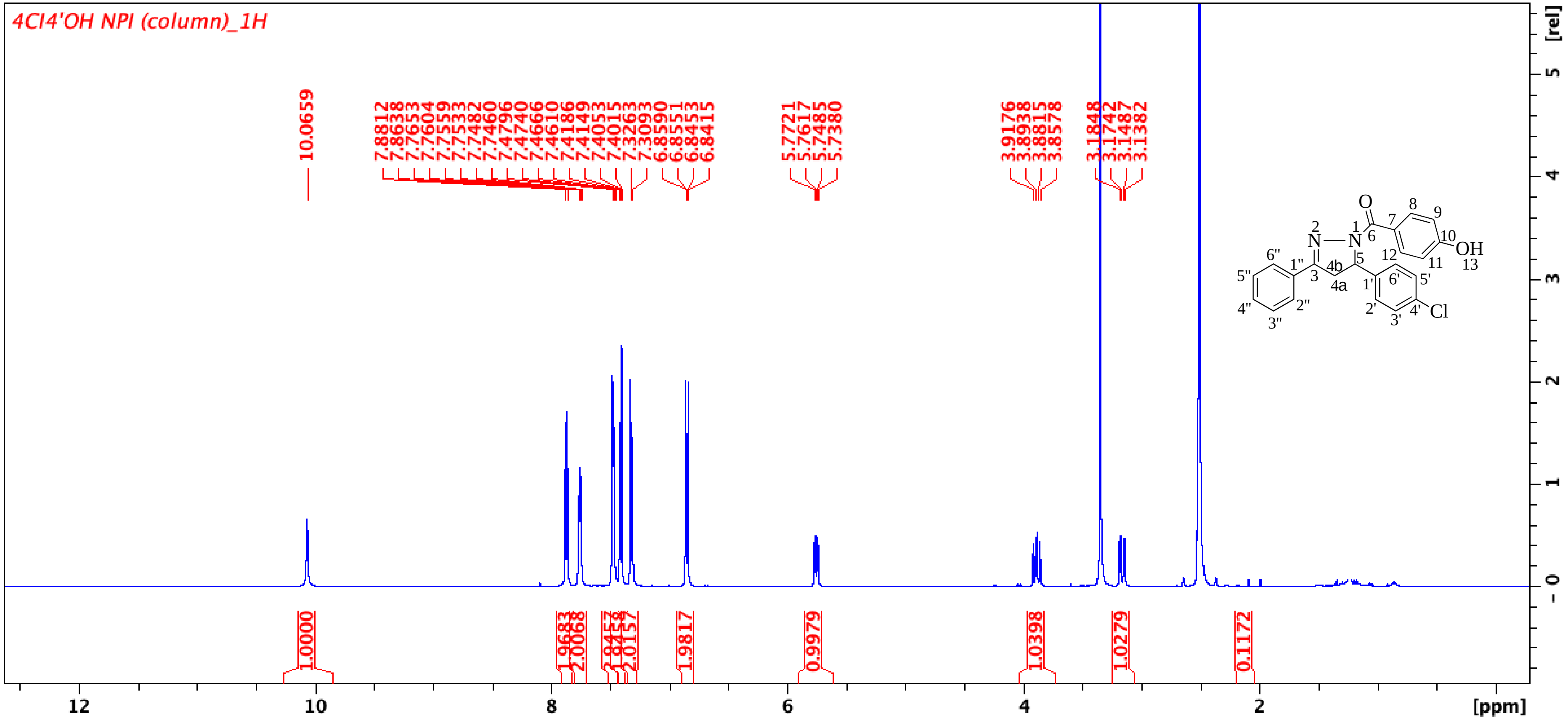

^{1}H NMR spectrum of compound **5c**

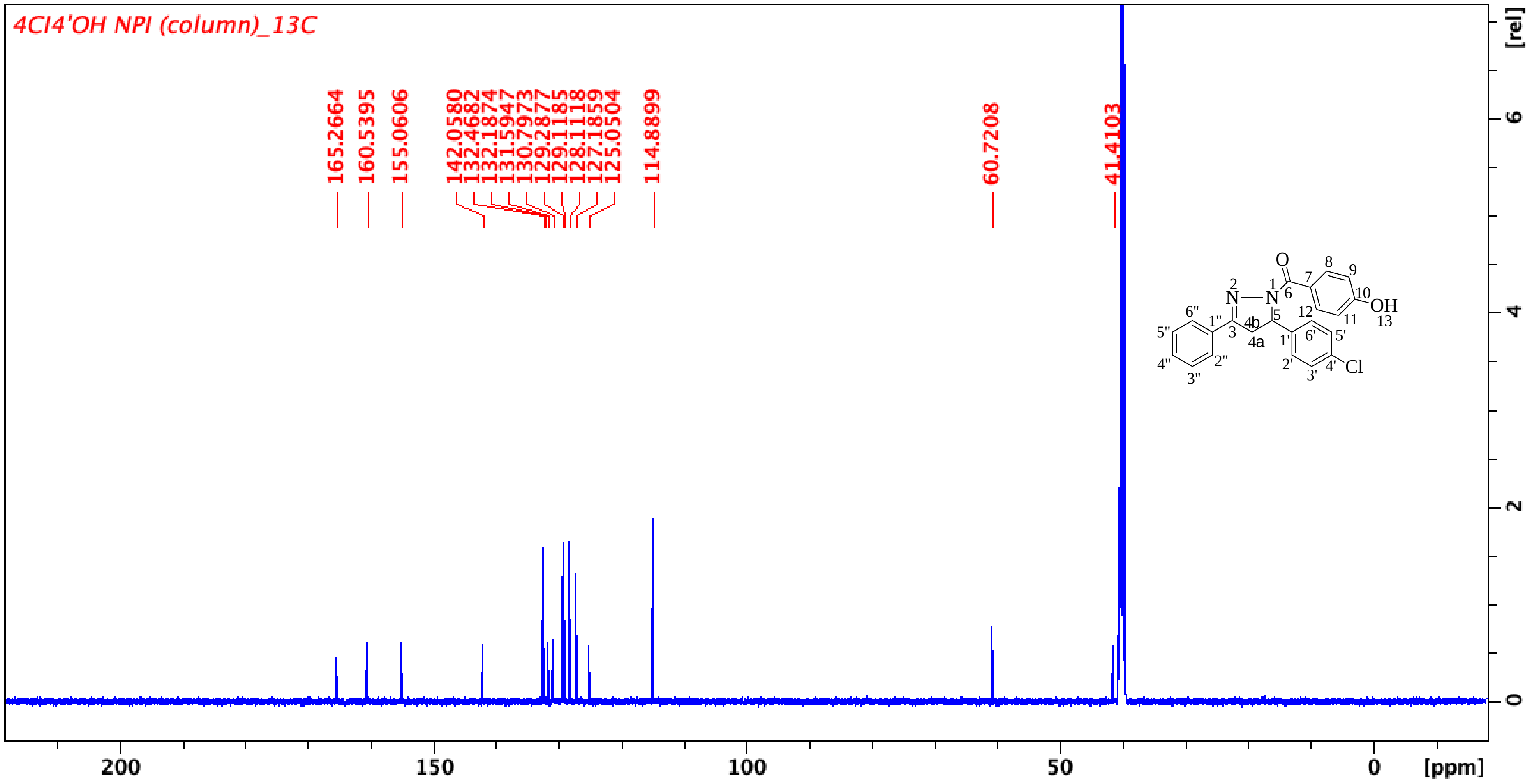

^{13}C NMR spectrum of compound **5c**

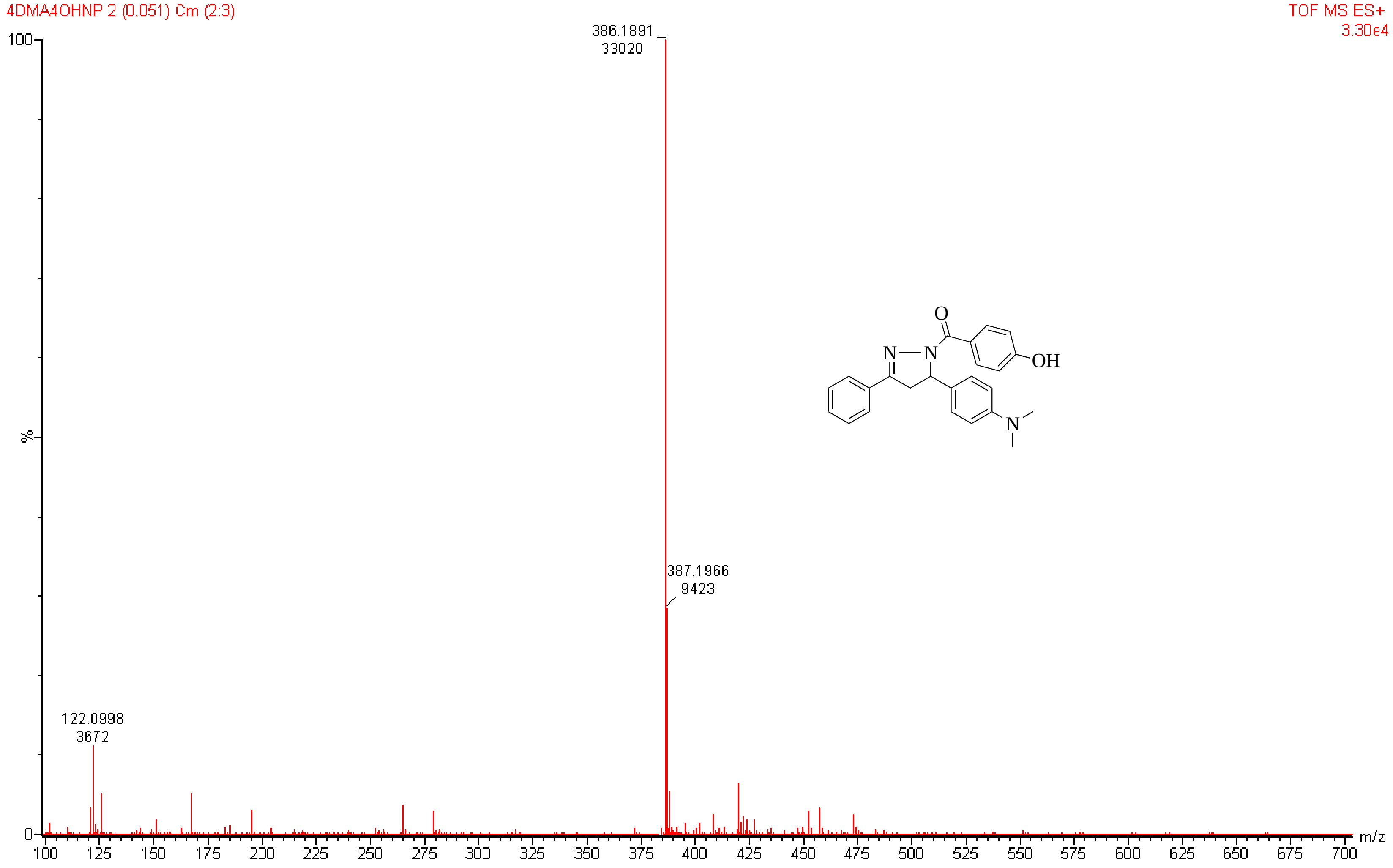

HRMS spectrum of compound **5d**

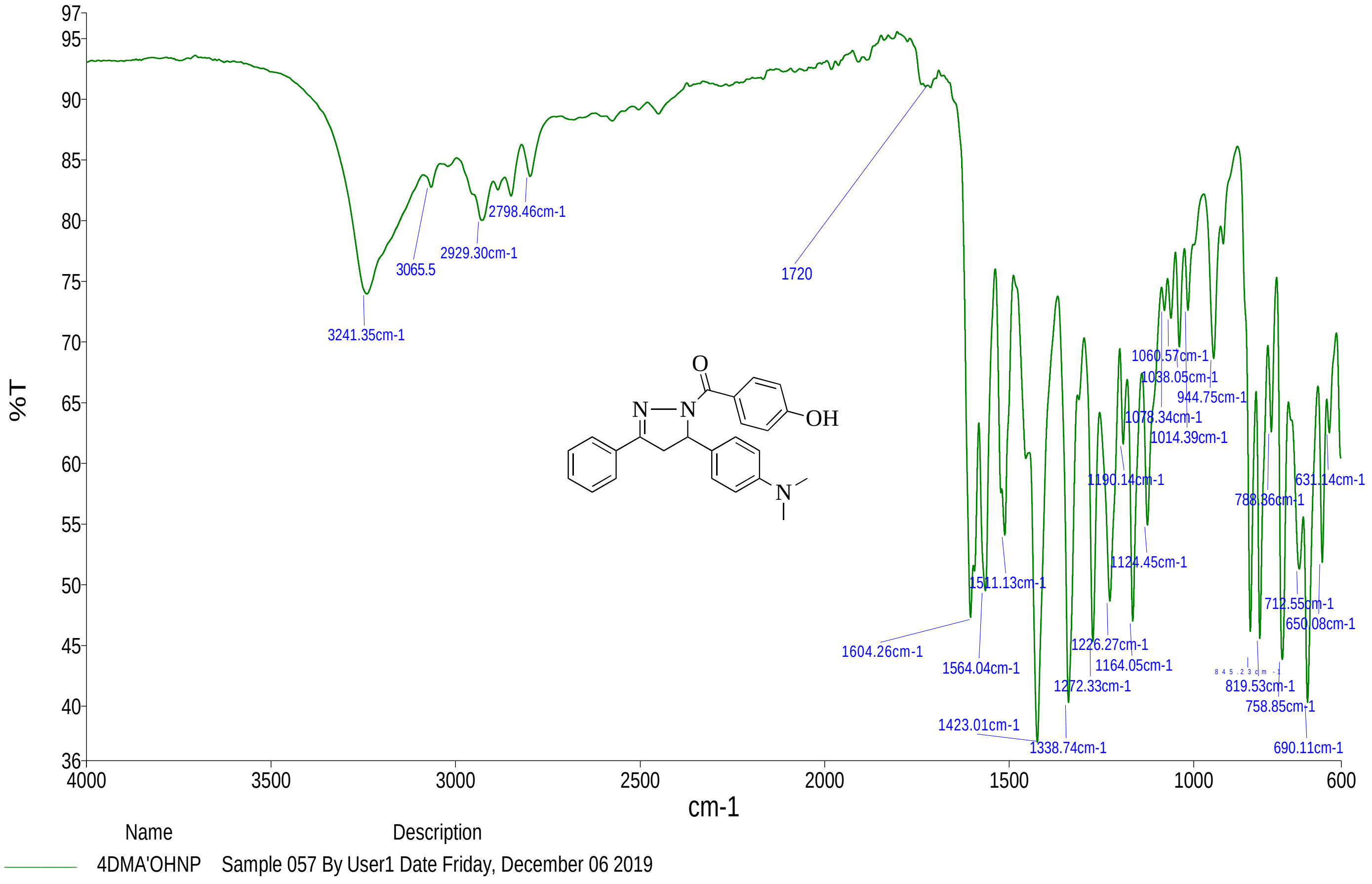

IR spectrum of compound **5d**

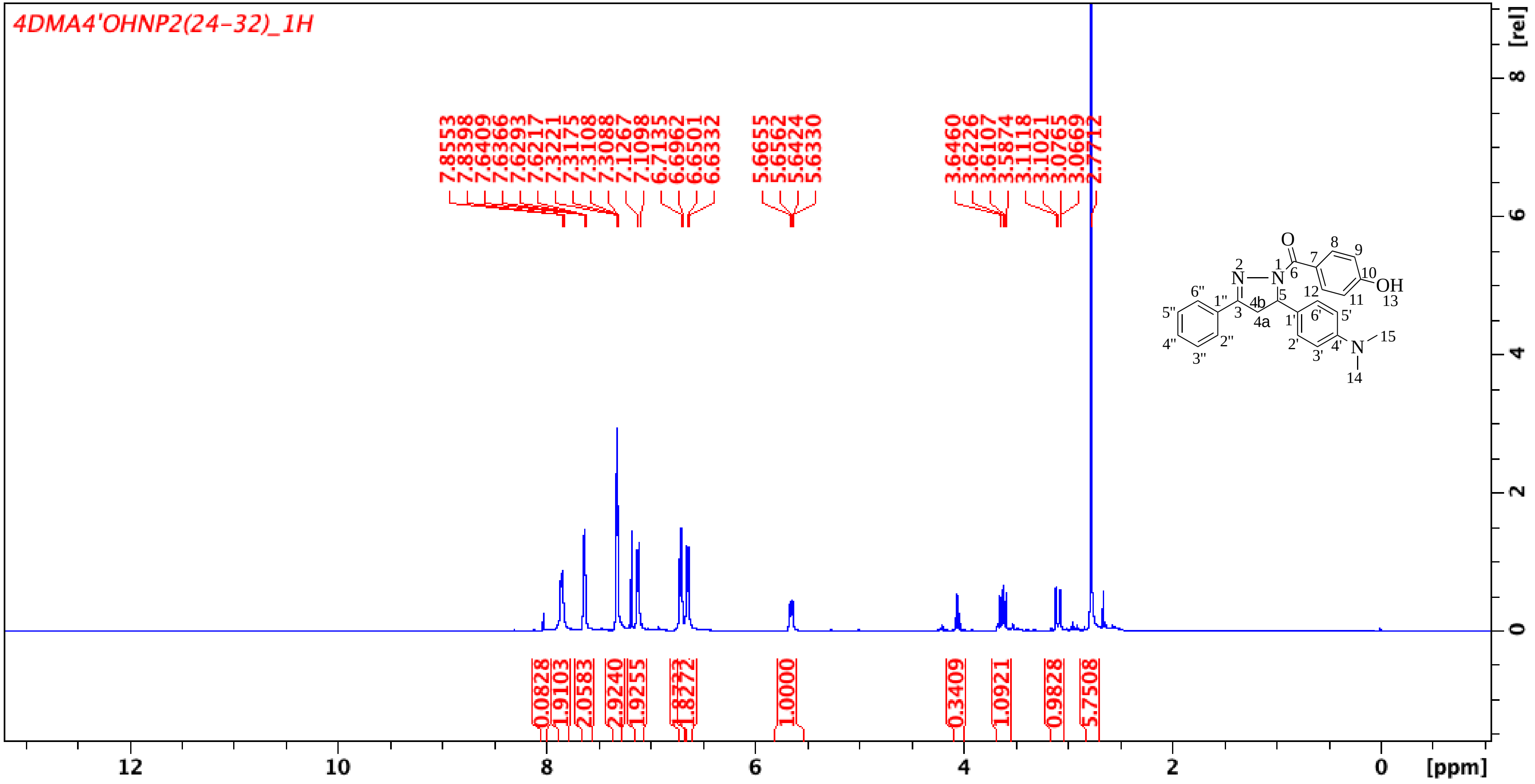

^{1}H NMR spectrum of compound **5d**

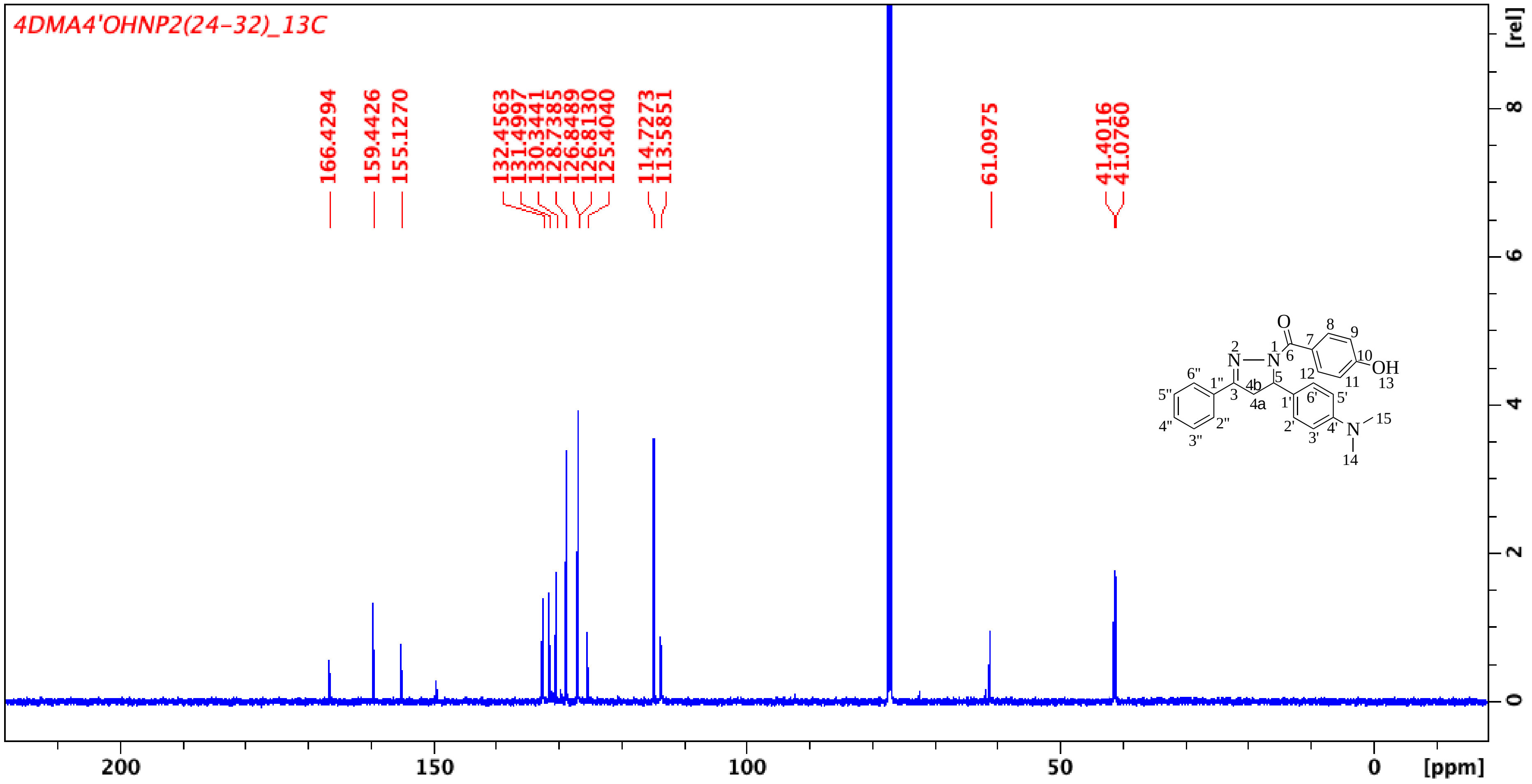

^{13}C NMR spectrum of compound **5d**

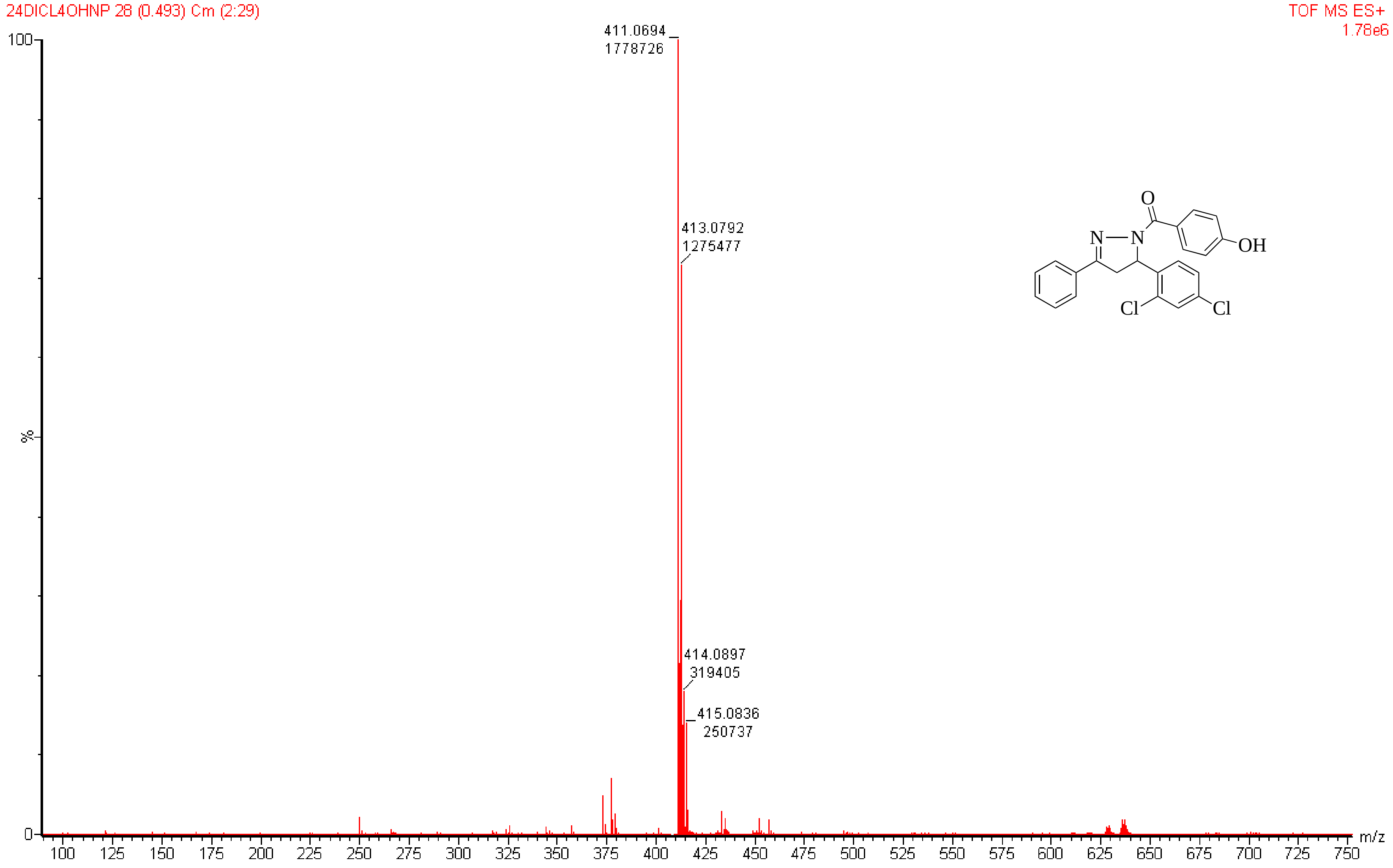

HRMS spectrum of compound **5e**

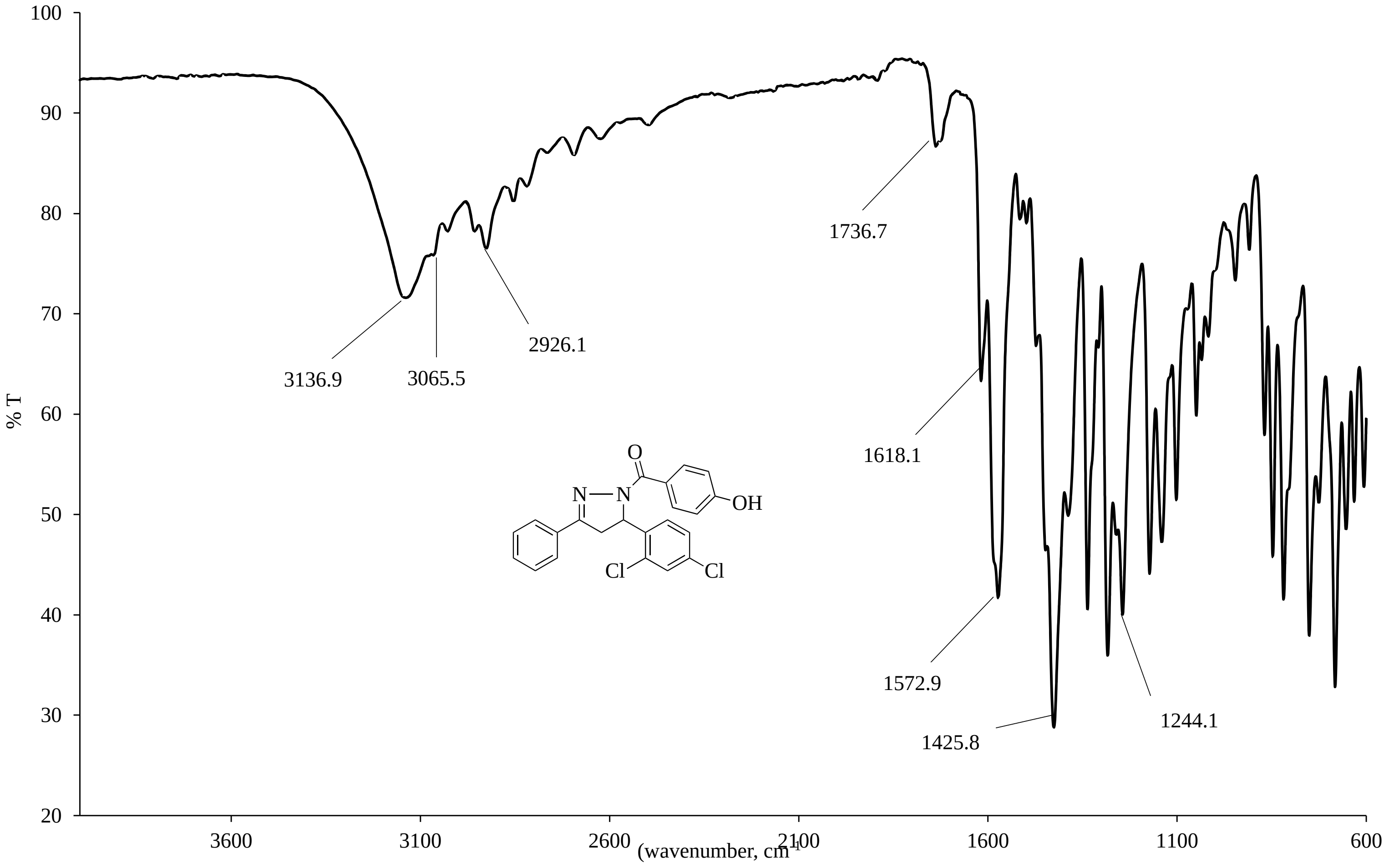

IR spectrum of compound **5e**

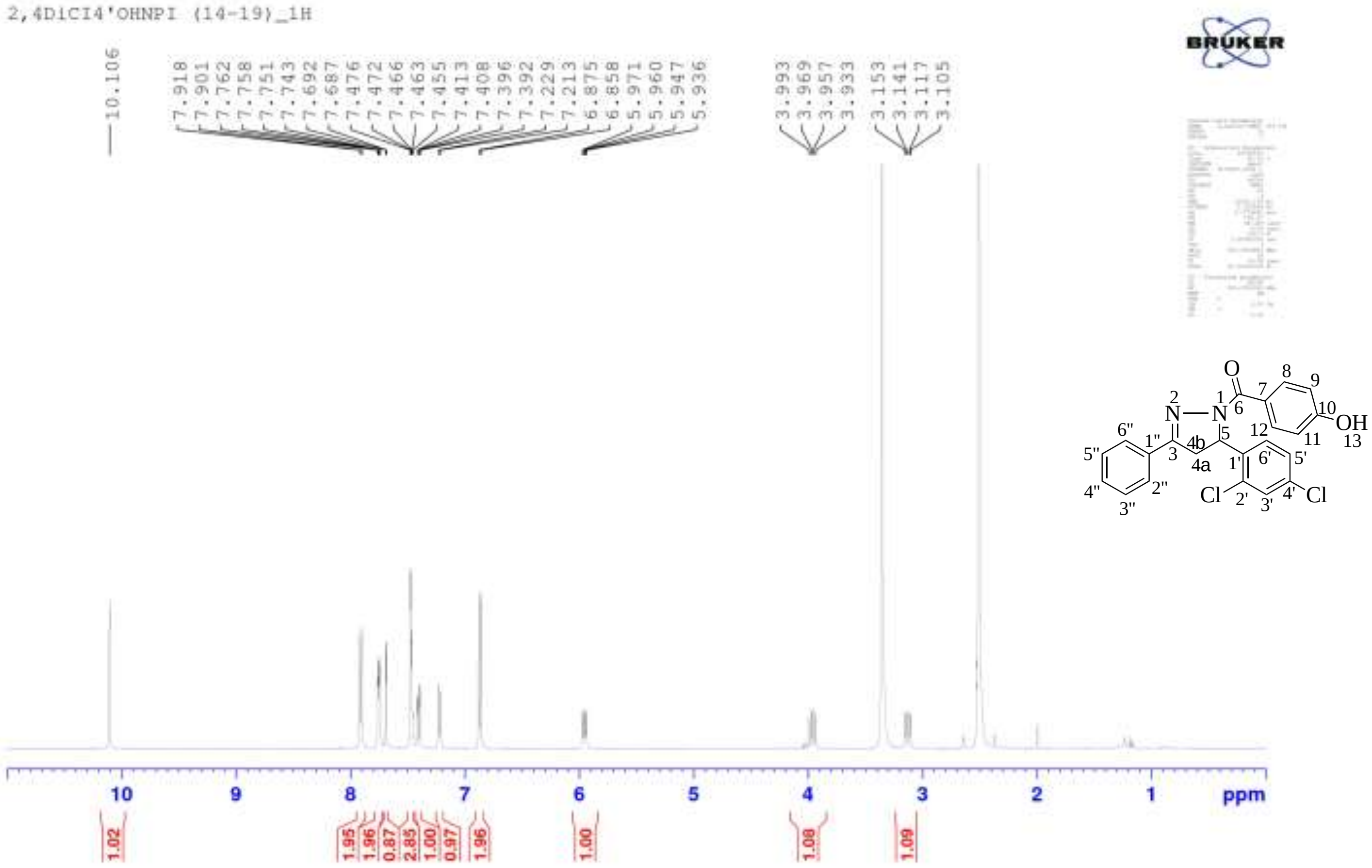

^{1}H NMR spectrum of compound **5e**

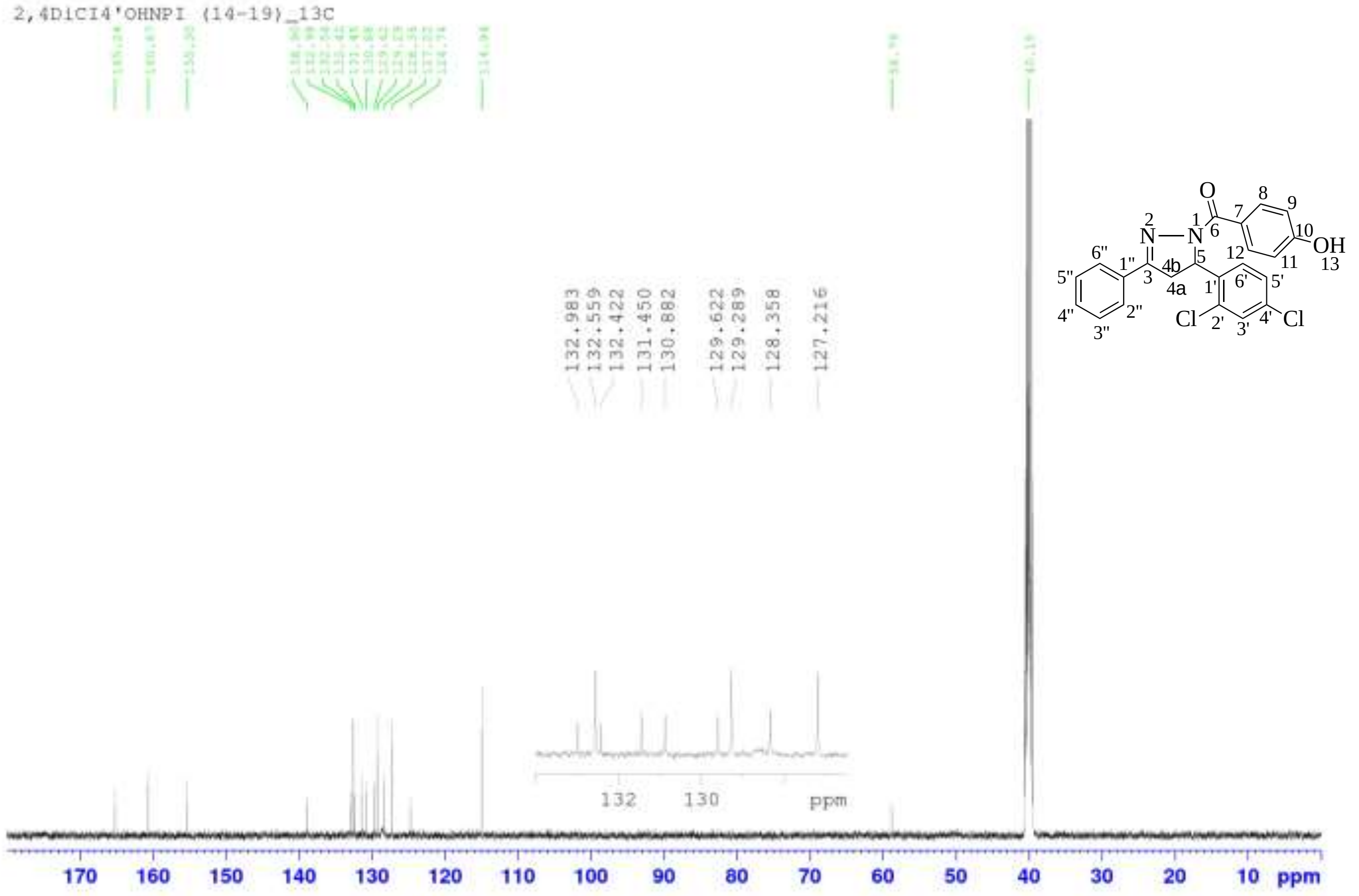

^{13}C NMR spectrum of compound **5e**

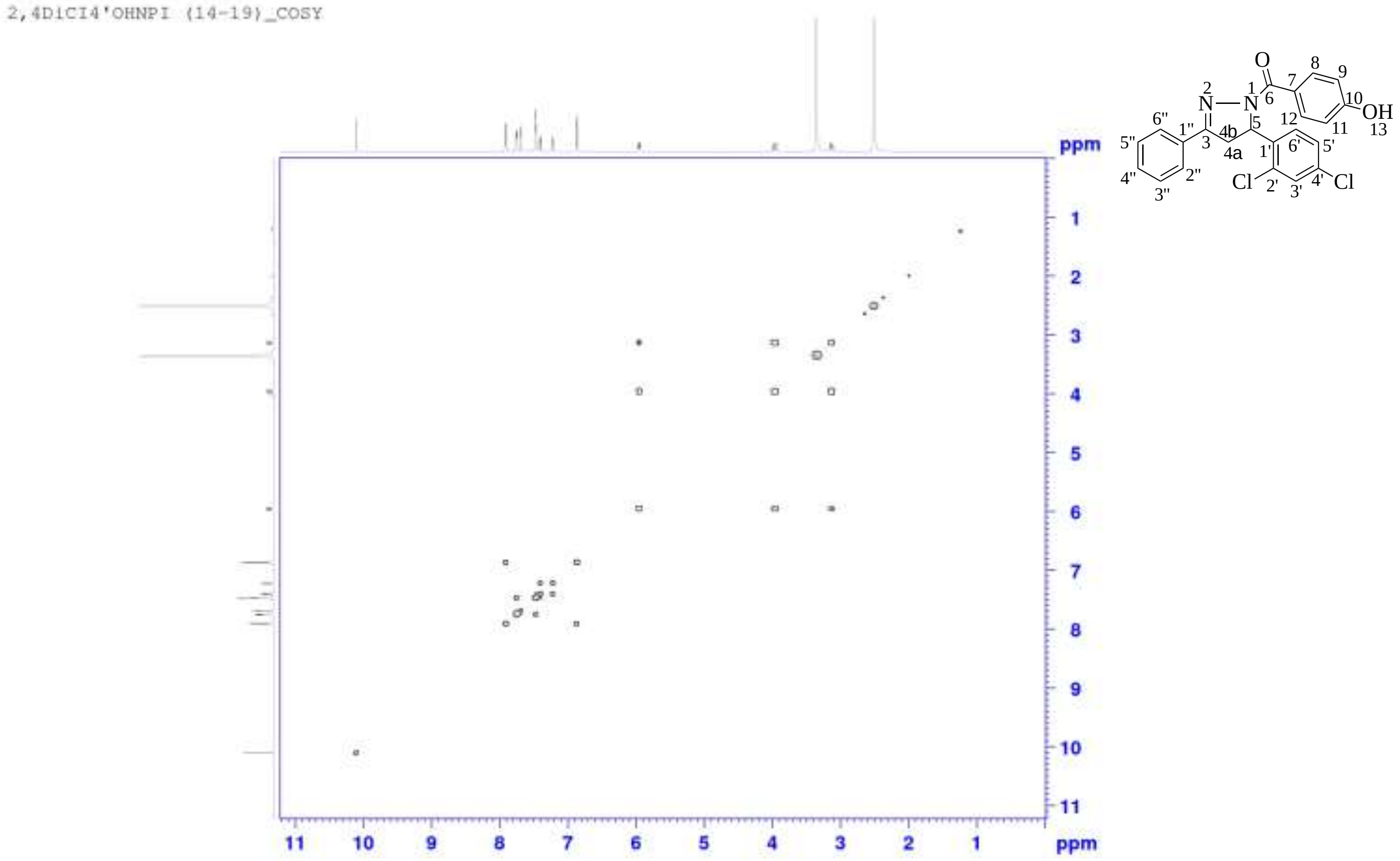

^{1}H-^{1}H COSY NMR spectrum of compound 5e

2,4DiCI4'OHNPI (14-19)_HSQC

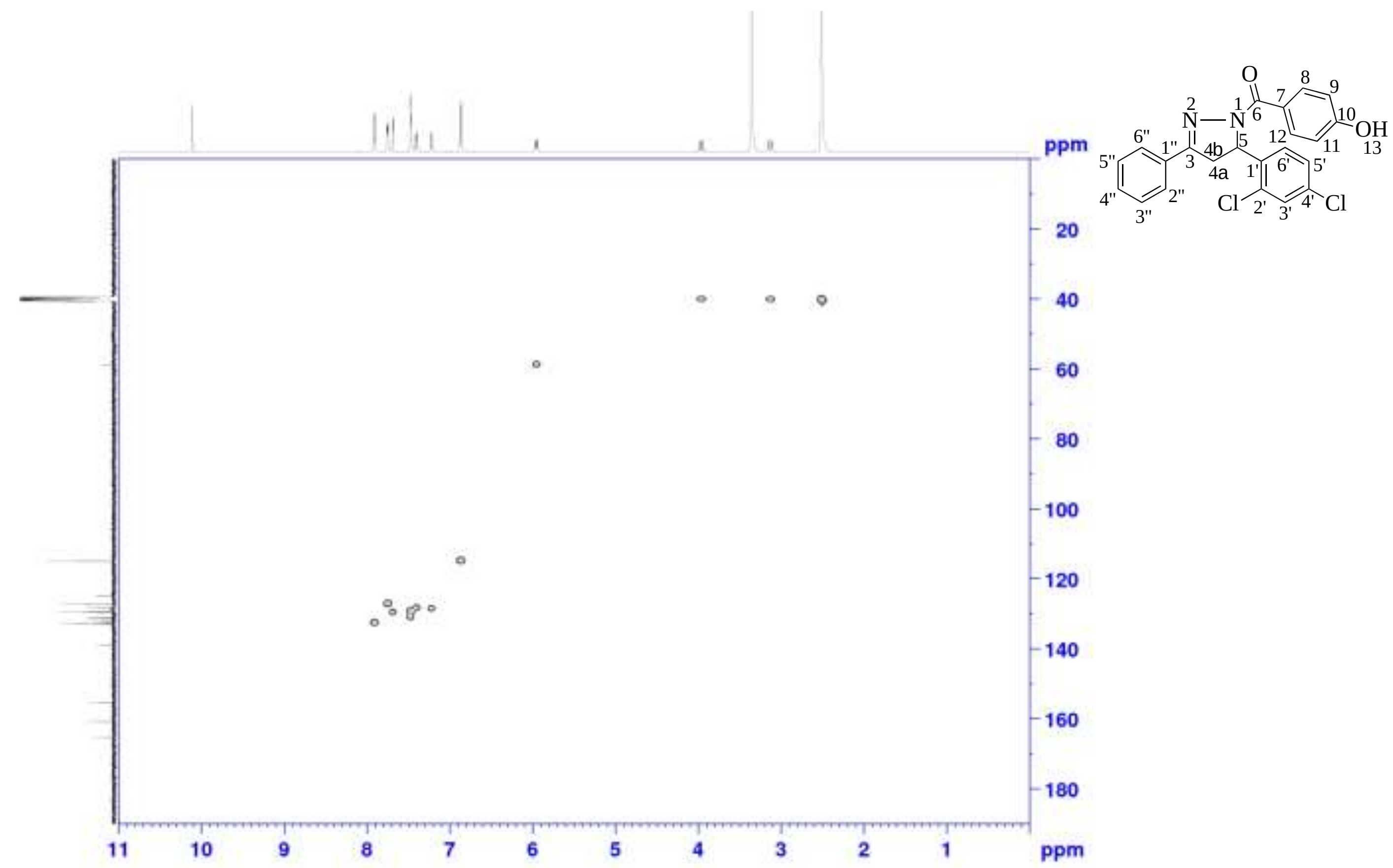

^{1}H-^{13}C HSQC NMR spectrum of compound 5e

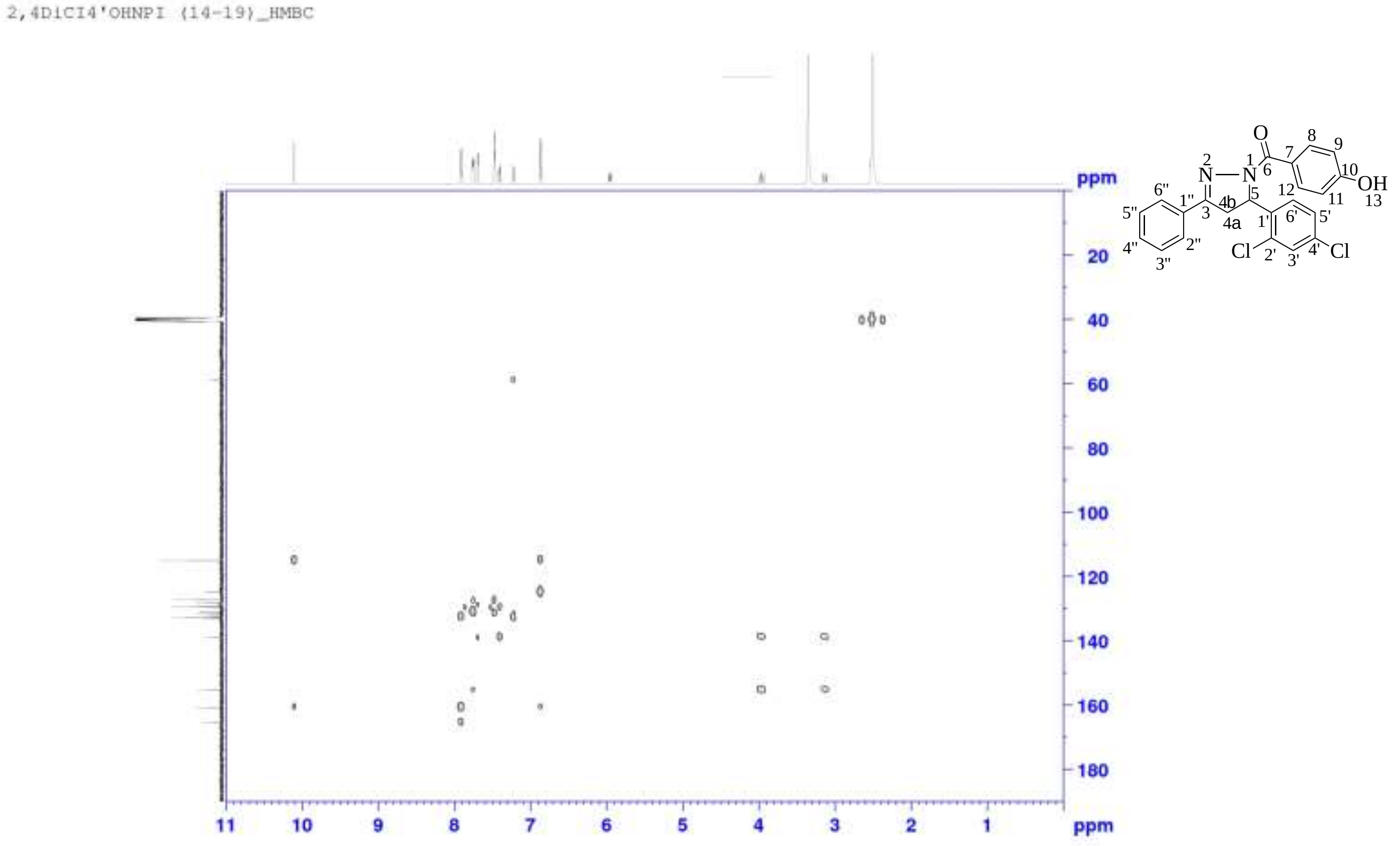

^{1}H-^{13}C HMBC NMR spectrum of compound 5e

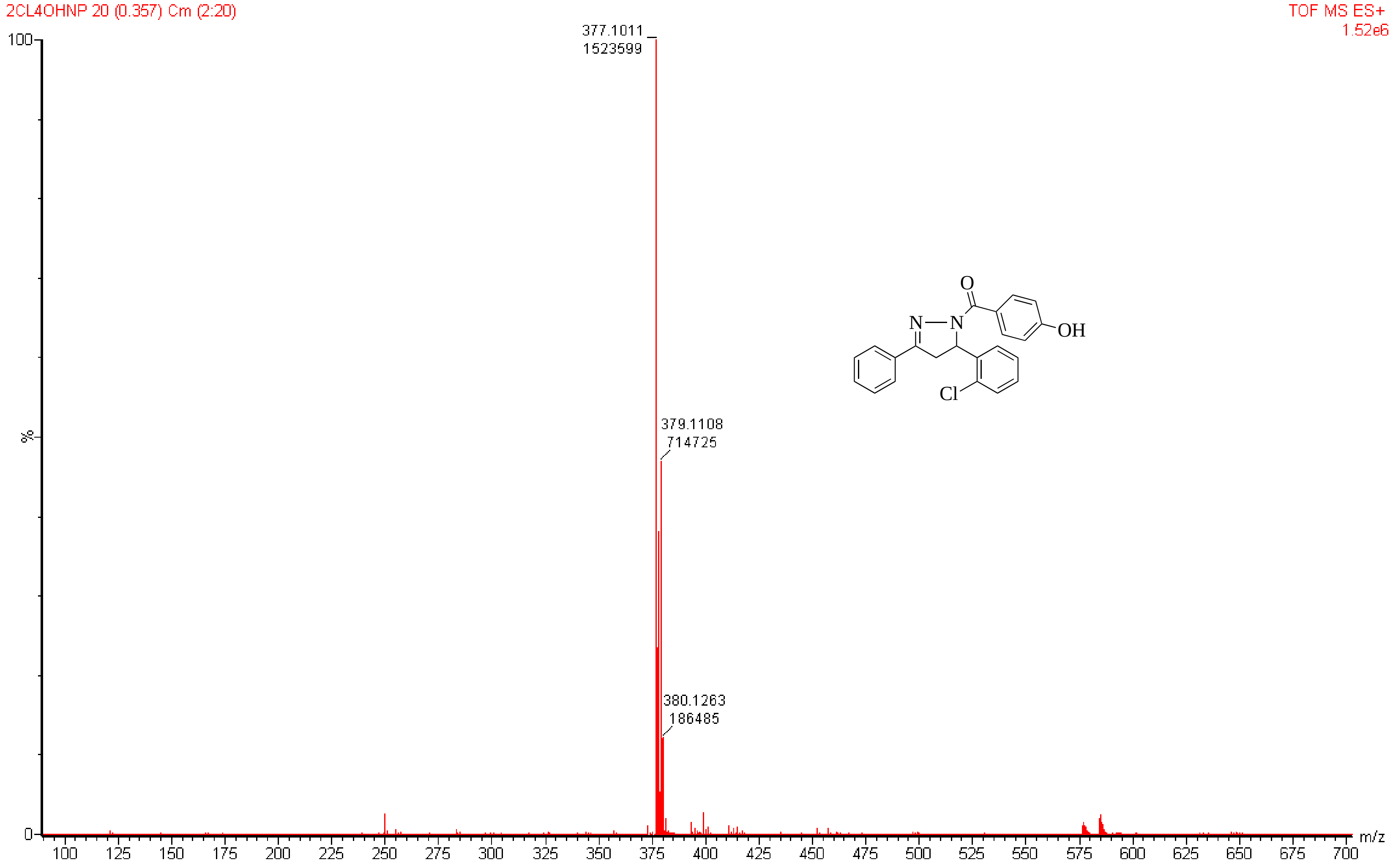

HRMS spectrum of compound **5f**

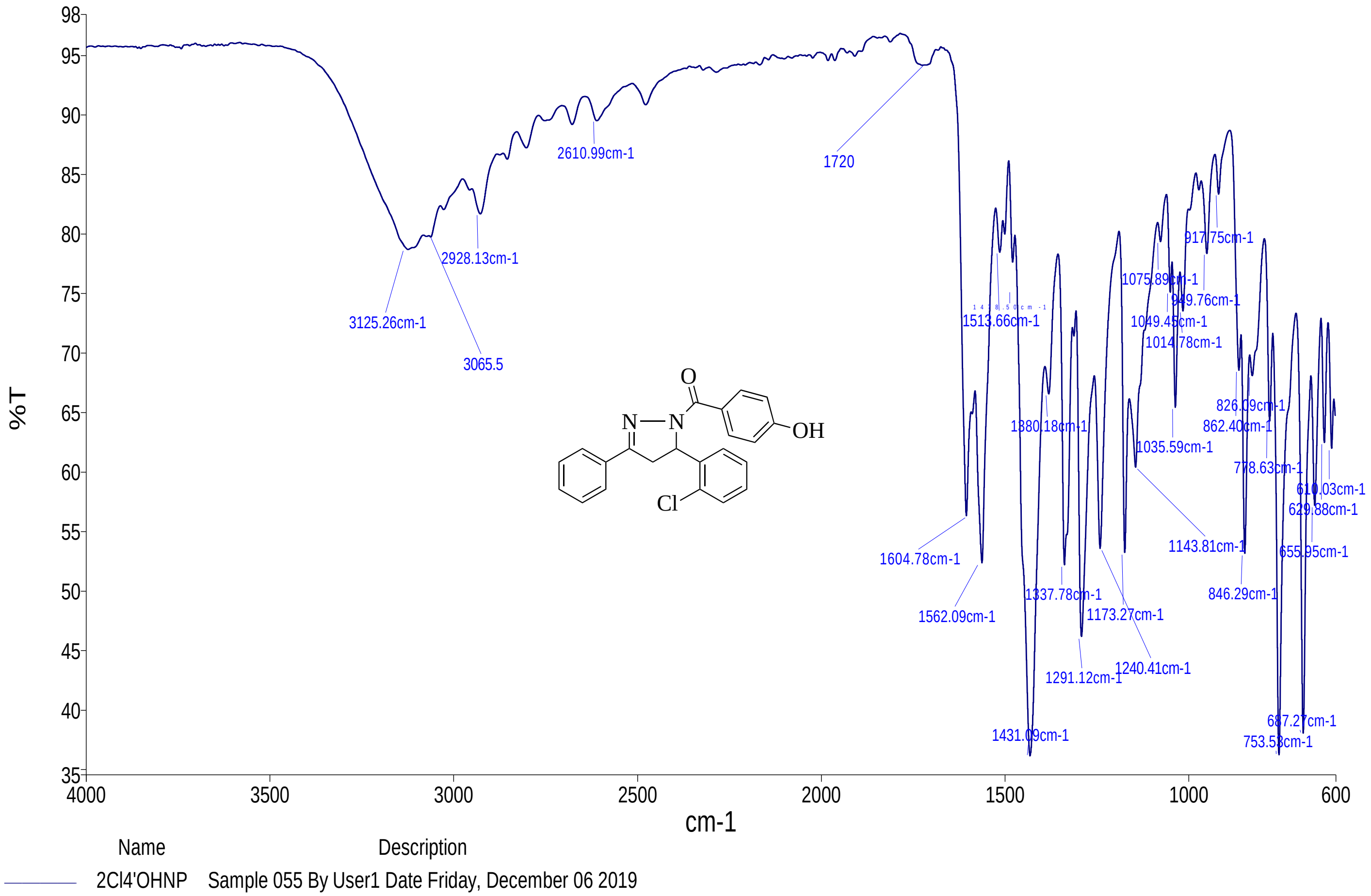

IR spectrum of compound **5f**

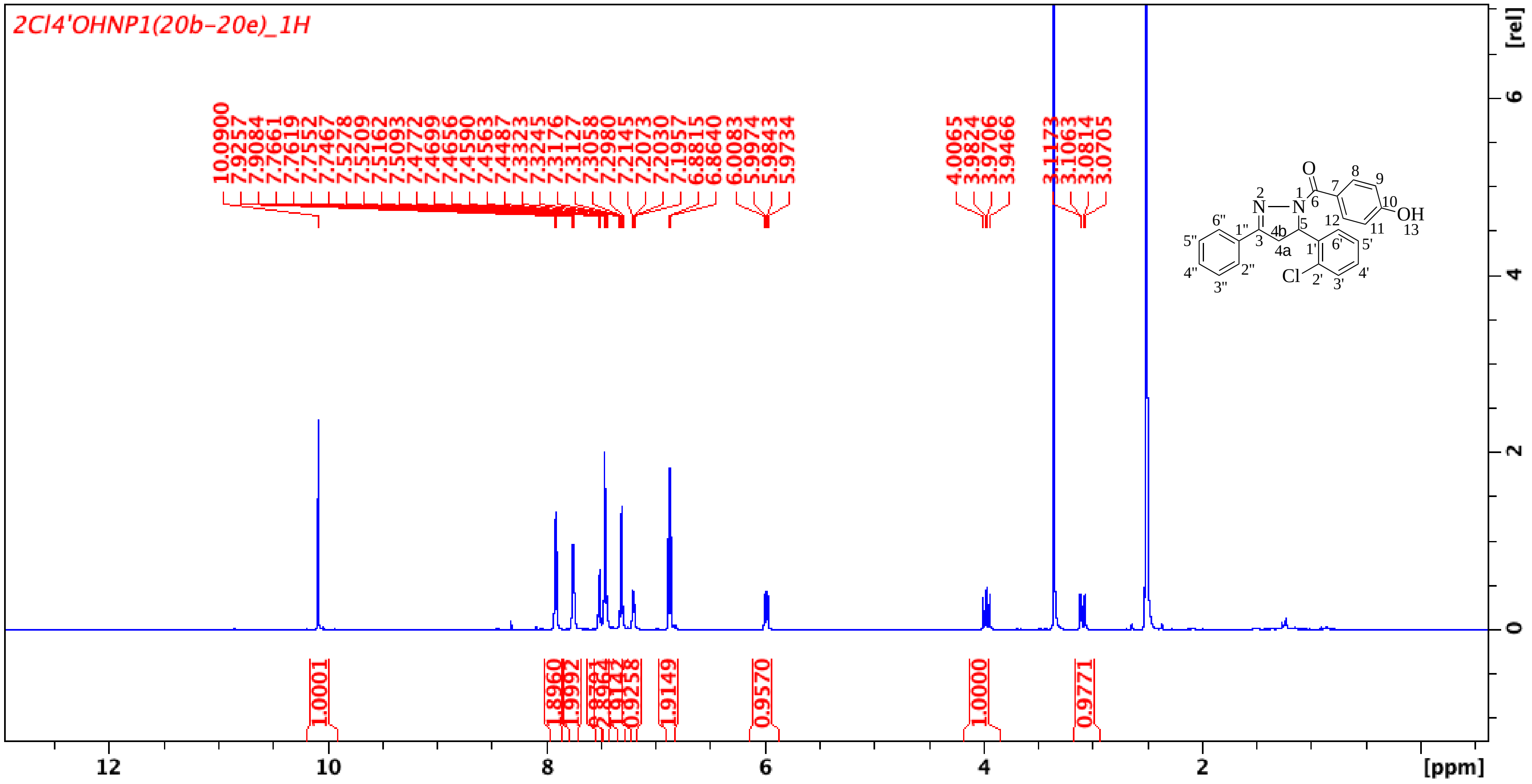

^{1}H NMR spectrum of compound **5f**

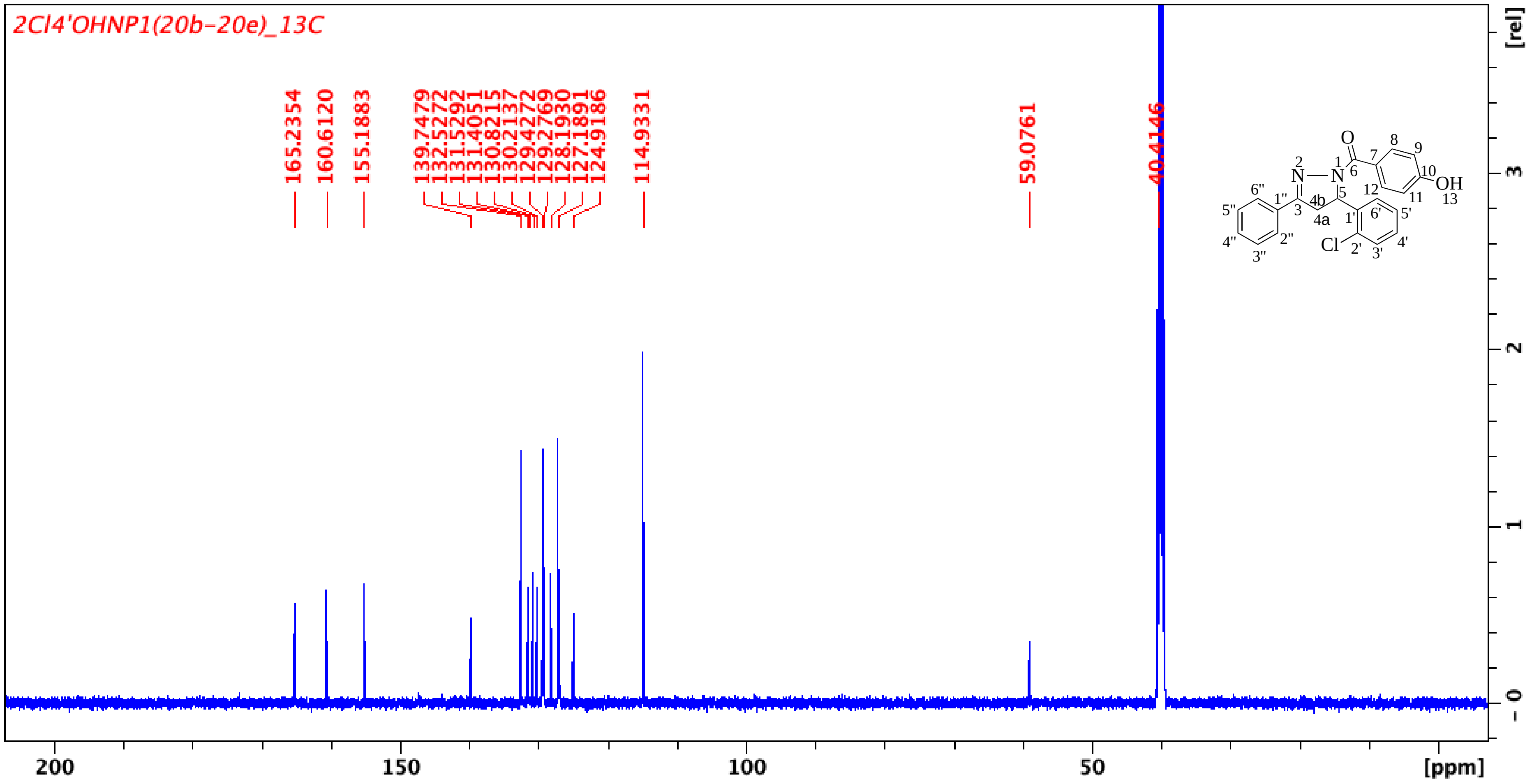

^{13}C NMR spectrum of compound **5f**

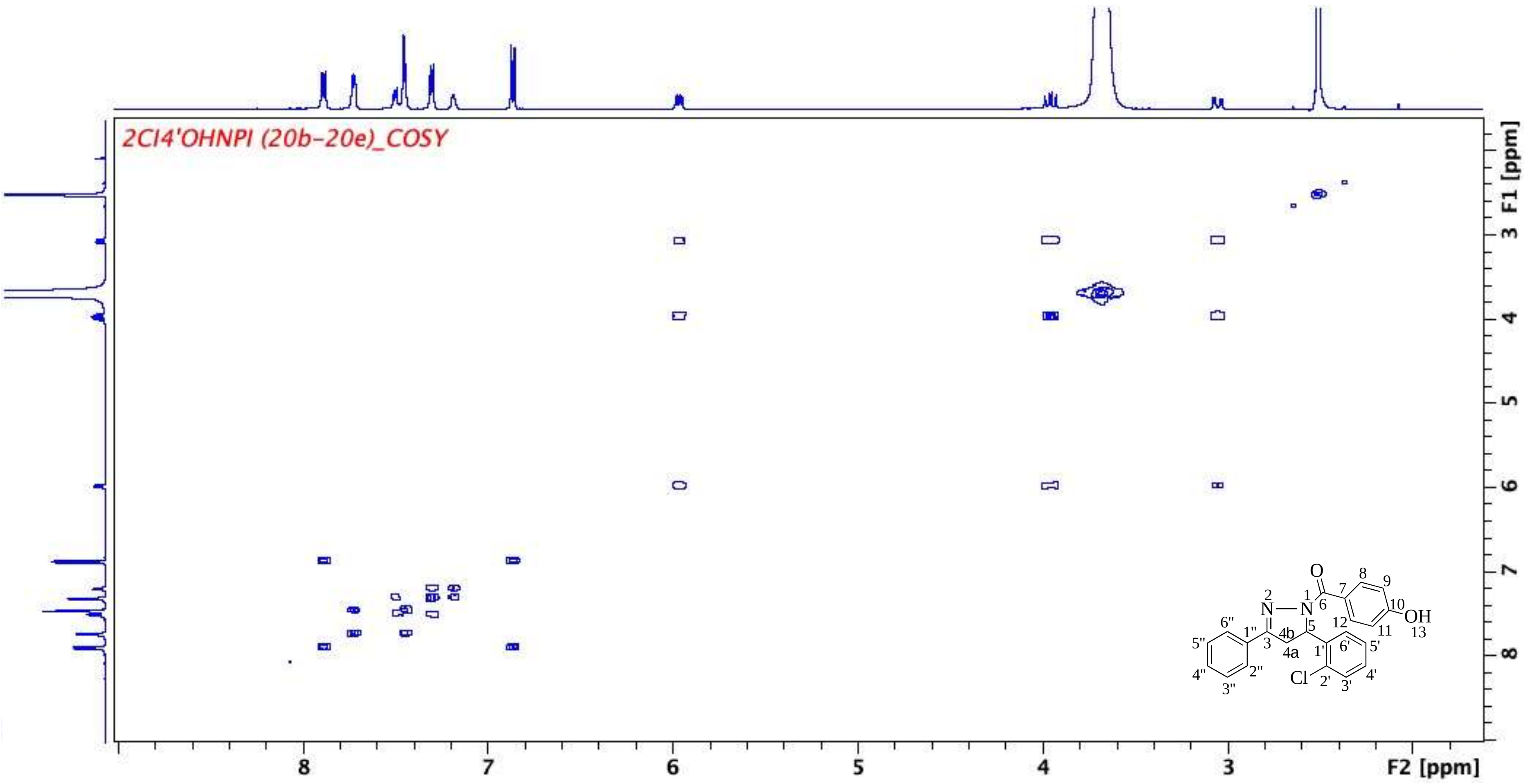

^{1}H-^{1}H COSY NMR spectrum of compound 5f

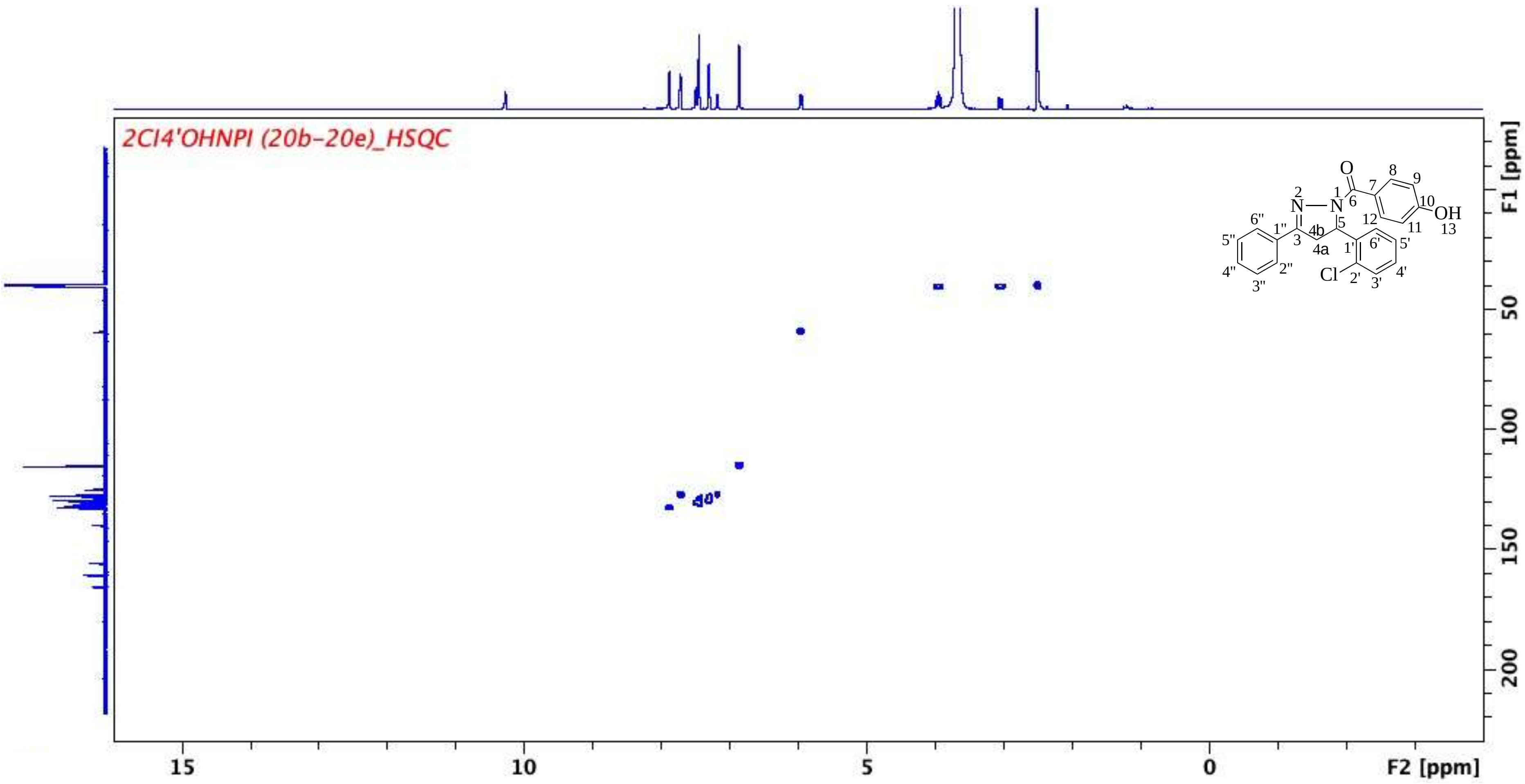

^{1}H-^{13}C HSQC NMR spectrum of compound 5f

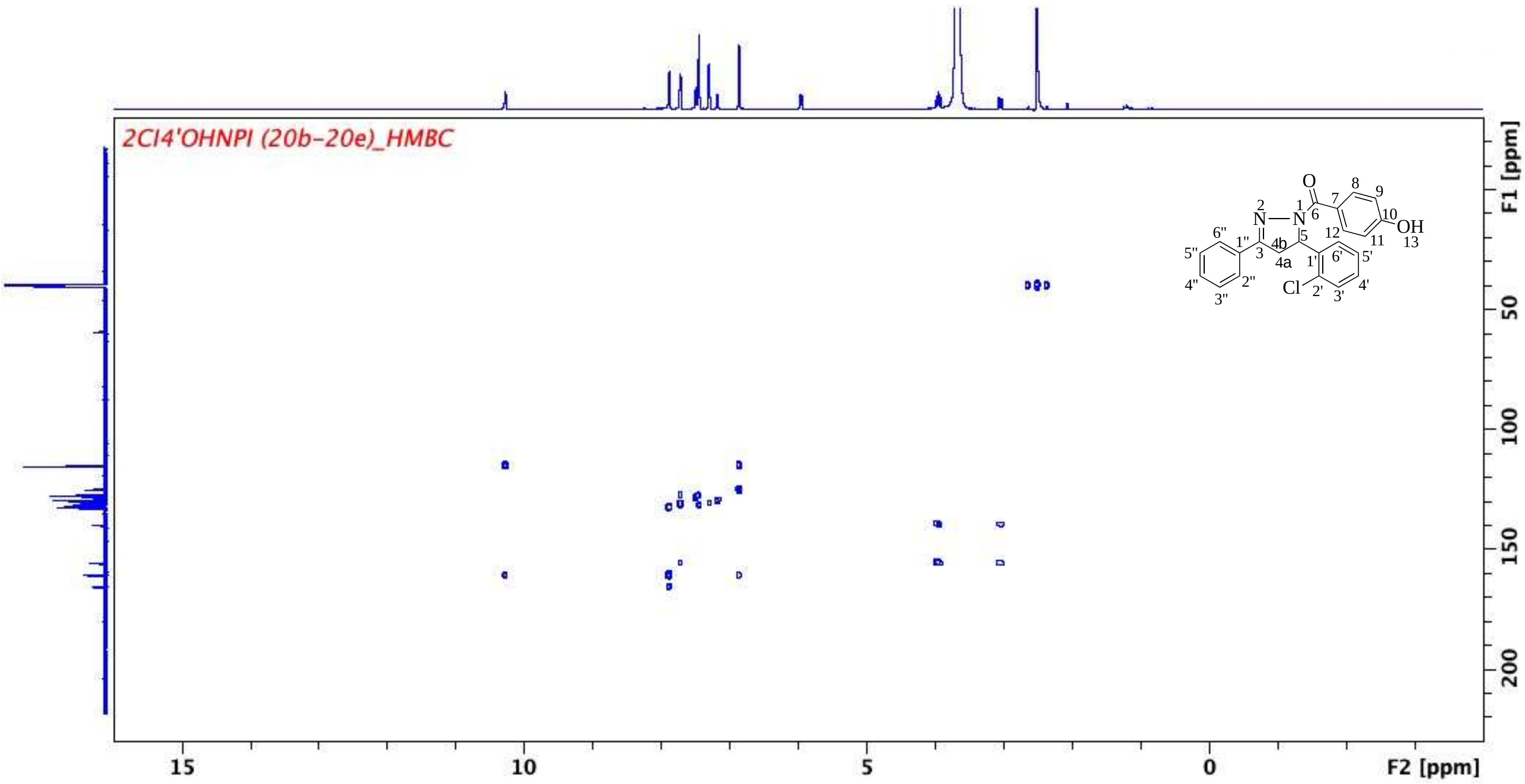

^{1}H-^{13}C HMBC NMR spectrum of compound 5f

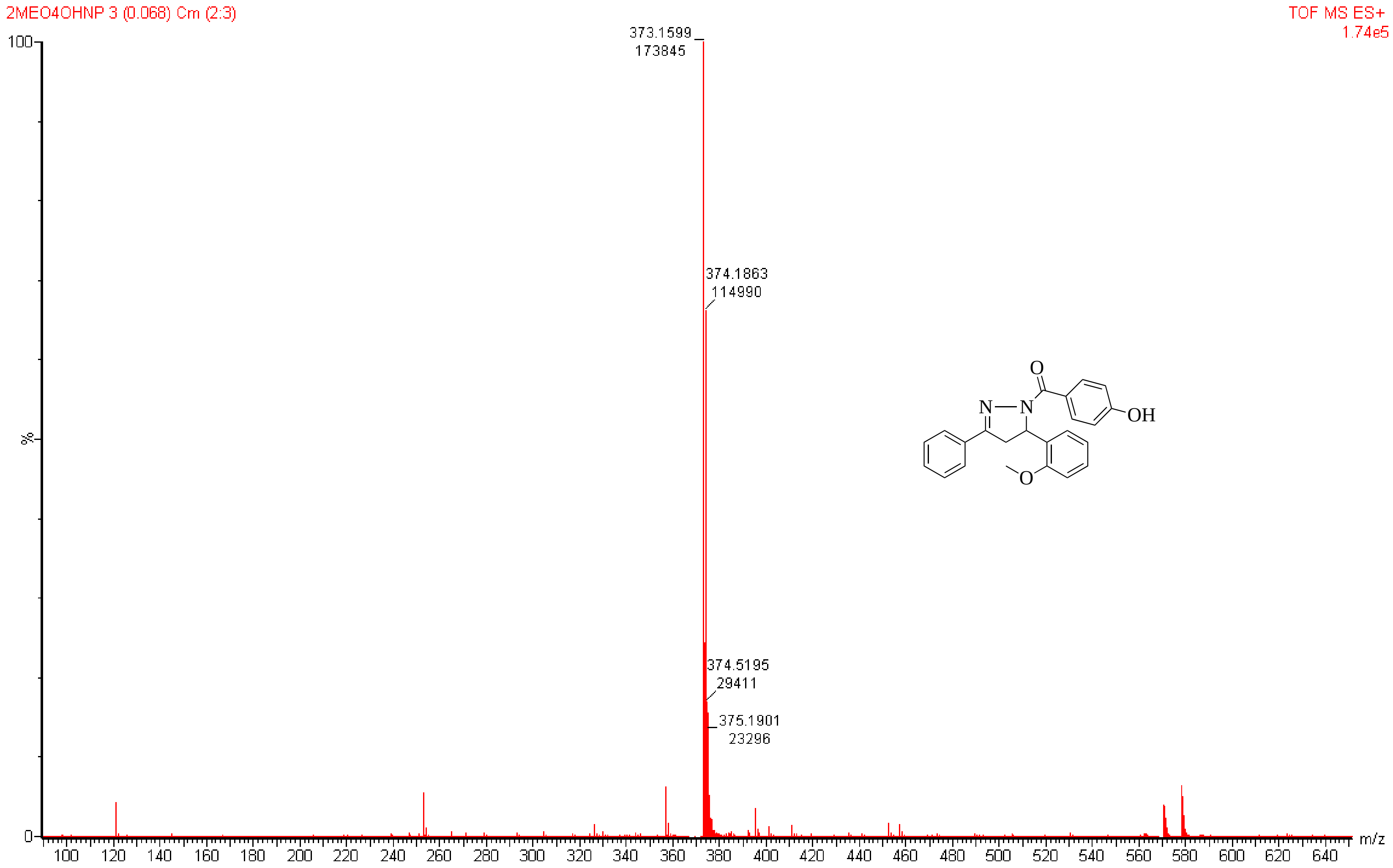

HRMS spectrum of compound **5g**

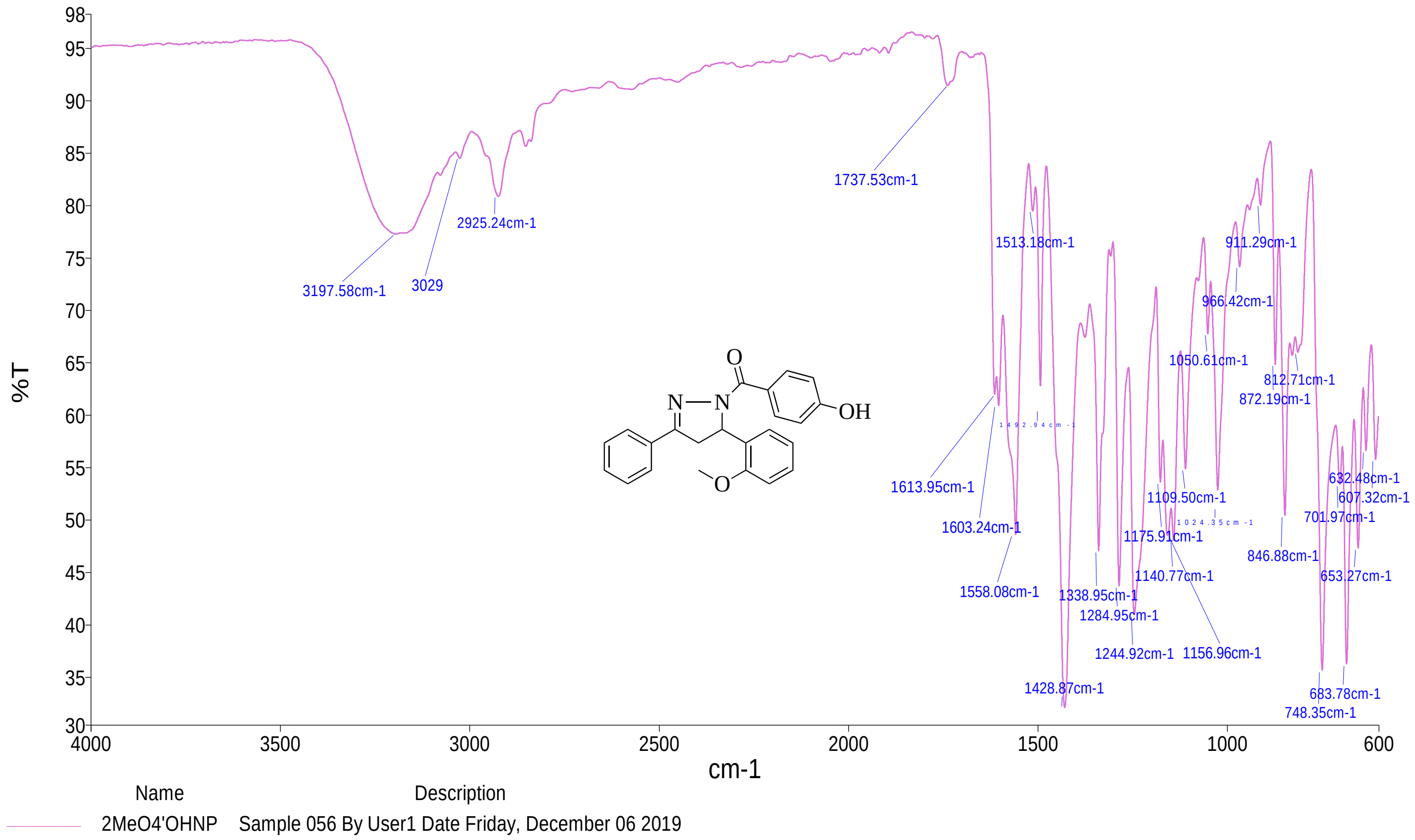

IR spectrum of compound **5g**

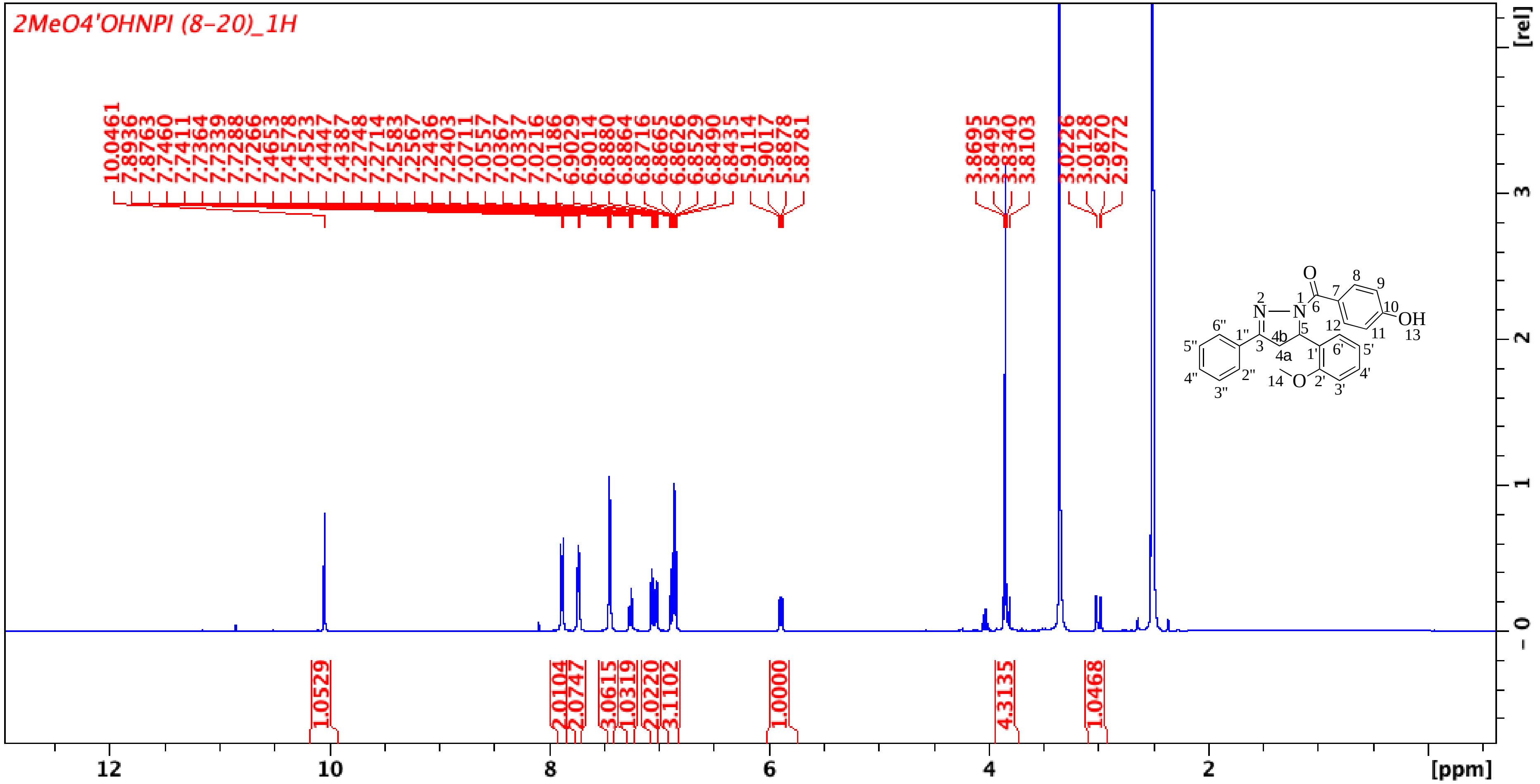

^{1}H NMR spectrum of compound **5g**

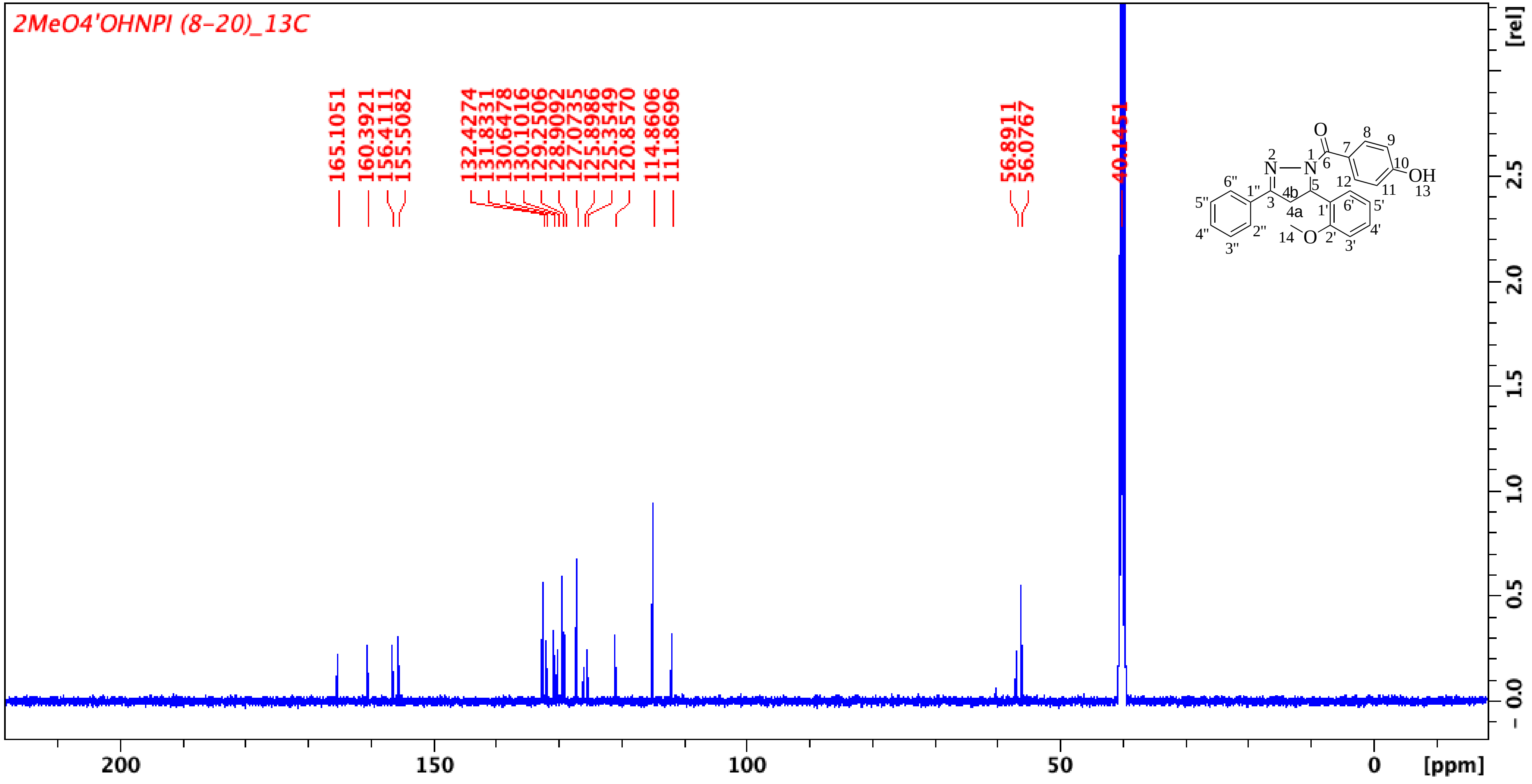

^{13}C NMR spectrum of compound **5g**

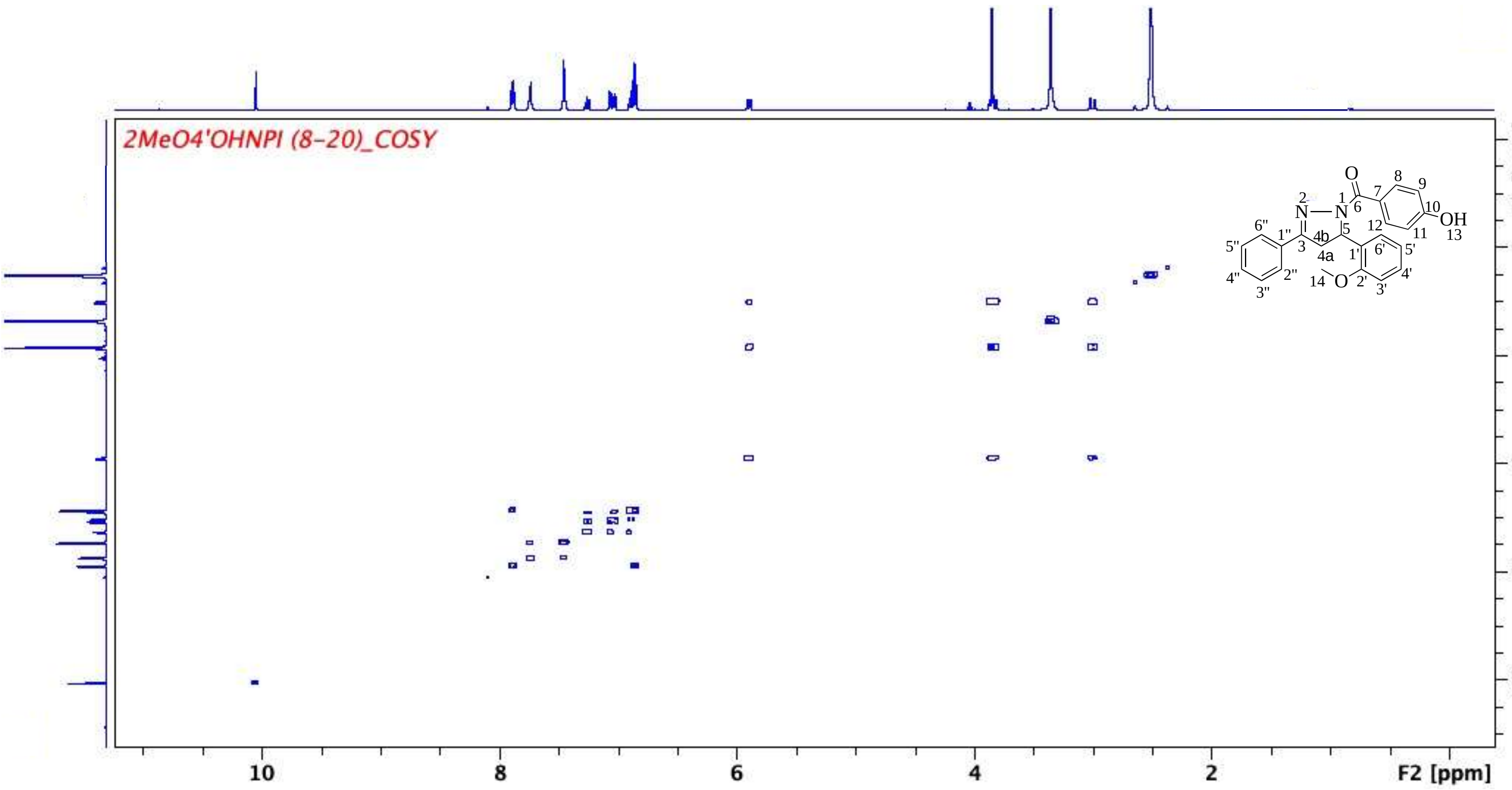

^{1}H-^{1}H COSY NMR spectrum of compound 5g

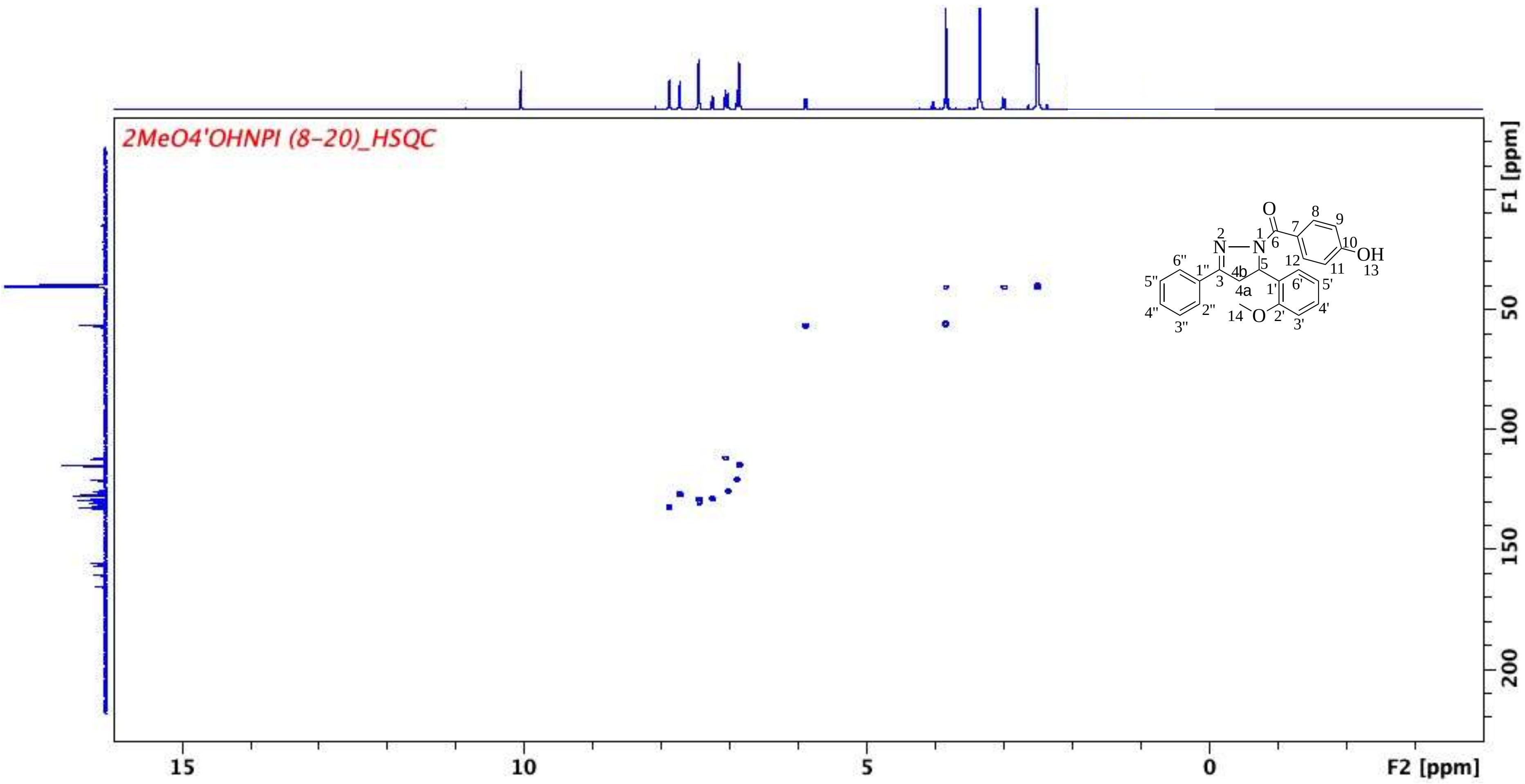

^{1}H-^{13}C HSQC NMR spectrum of compound 5g

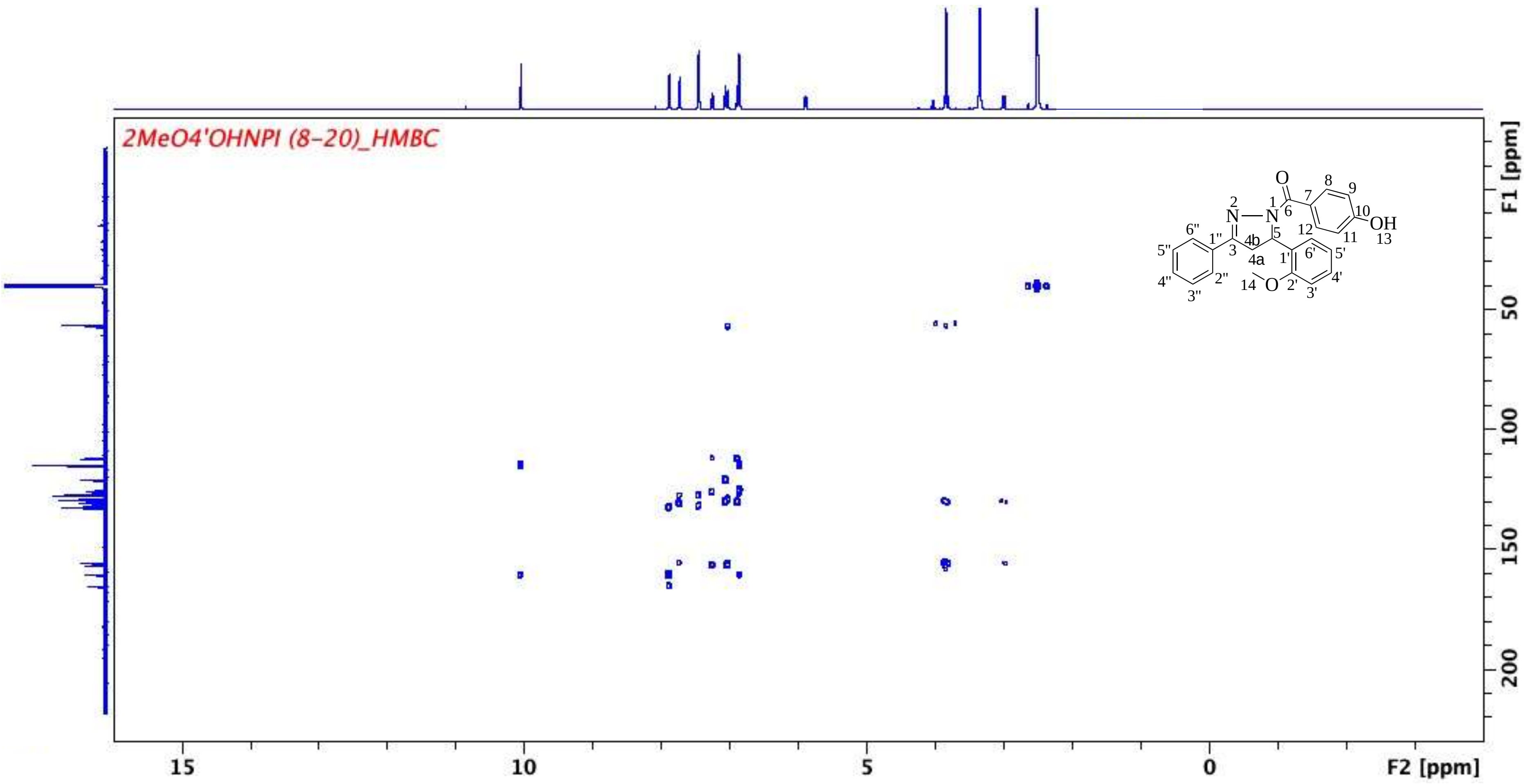

^{1}H-^{13}C HMBC NMR spectrum of compound 5g
